# Stimulating Chiral Selective Expression of Room Temperature Phosphorescence for Chirality Recognition

**DOI:** 10.1002/advs.202410671

**Published:** 2024-10-08

**Authors:** Zhisheng Gao, Xin Yan, Qi Jia, Jingru Zhang, Guangyao Guo, Huanhuan Li, Hui Li, Gaozhan Xie, Ye Tao, Runfeng Chen

**Affiliations:** ^1^ State Key Laboratory of Organic Electronics and Information Displays & Institute of Advanced Materials (IAM) Nanjing University of Posts & Telecommunications Nanjing 210023 China; ^2^ Songshan Lake Materials Laboratory Dongguan Guangdong 523808 China

**Keywords:** chiral recognition, circularly polarized luminescence, energy transfer, host–guest doping, room temperature phosphorescence

## Abstract

Chiral recognition is crucial for applications in chiral purity assessment and biomedical fields. However, achieving chiral recognition through visible room temperature phosphorescence remains challenging. Here, two chiral molecules, designated as host and guest are synthesized, which possess similar structural configurations. A viable strategy involving a chiral configuration‐dependent energy transfer process to enable selective phosphorescence expression is proposed, thereby enabling chiral recognition in a host‐guest doping system. The chiral and structural similarity between host and guest facilitates efficient Dexter energy transfer due to the reduced spatial distance between the molecules. This mechanism significantly enhances the intensity of red phosphorescence from the guest molecule, characterized by an emission peak at 612 nm and a prolonged lifetime of 32.7 ms. This work elucidates the mechanism of chiral‐dependent energy transfer, demonstrating its potential for selectively expressing phosphorescence in chiral recognition.

## Graphical abstract

A facile strategy that leverages the energy transfer process dependent on chiral configuration is proposed. This approach allows for selective activation of phosphorescence from the doped chiral guest, thereby facilitating efficient chiral recognition within a host‐guest doping system.

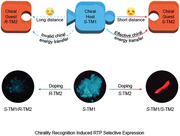

## 1 Introduction

Chiral molecules exhibit enantiomeric symmetry, yet their biological activities can differ markedly.^[^ ^1^ ^]^ The identification and analysis of chiral molecules are critically important across various scientific domains, including biology, pharmaceutical design, and chemistry.^[^ ^2^ ^]^ In life sciences,^[^ ^3^ ^]^ systematically identifying chiral selectivity within organisms is significant for unraveling fundamental biological processes. In medicine,^[^ ^4^ ^]^ precise recognition of chiral pharmaceuticals is essential to mitigate side effects and enhance therapeutic outcomes. In catalysis chemistry,^[^ ^5^ ^]^ chiral recognition plays a crucial role in promoting efficient reactions. For optoelectronic applications,^[^ ^6^ ^]^ chiral luminescence represents a transformative advancement, offering a revolutionary pathway for enhancing light‐emitting technologies. To achieve efficient recognition of chiral molecules, various strategies have been proposed, including chiral chromatography,^[^ ^7^ ^]^ enzymatic resolution,^[^ ^8^ ^]^ and circular dichroism spectroscopy,^[^ ^9^ ^]^ as well as chiral liquid crystals,^[^ ^10^ ^]^ fluorescent probes,^[^ ^11^ ^]^ and chiral nanoparticles.^[^ ^12^ ^]^ Fluorescent probes, in particular, are noted for their specific fluorophores and binding sites, which respond distinctly to different enantiomers of chiral substrates. This allows for intuitive chiral recognition and monitoring of optical purity through observable changes in luminescent color and intensity. However, fluorescence interference from solvents and other substances may compromise detection accuracy, underscoring the necessity for advanced techniques and materials that can selectively reduce or eliminate such background interference.^[^ ^13^ ^]^

Room temperature phosphorescence (RTP) materials have attracted widespread interest due to their long triplet‐state lifetimes and large Stokes shifts, which allow their luminescence to be distinctly recognized after the removal of the excitation light source.^[^ ^14^ ^]^ The ultralong lifetime of RTP emission effectively suppresses background fluorescence, thereby enhancing the signal‐to‐noise ratio and positioning RTP materials as promising analytical tools for detecting analytes. The host‐guest energy transfer strategy has been proven to be an efficient strategy for developing RTP materials,^[^ ^15^ ^]^ in which the RTP properties of the guest molecules can be rationally regulated through tuning the interaction between the host and guest. Therefore, a significant enhancement in RTP emission can be achieved under ambient conditions by controlling the configuration of the chiral host and guest in the doping systems.

**Figure 1:**
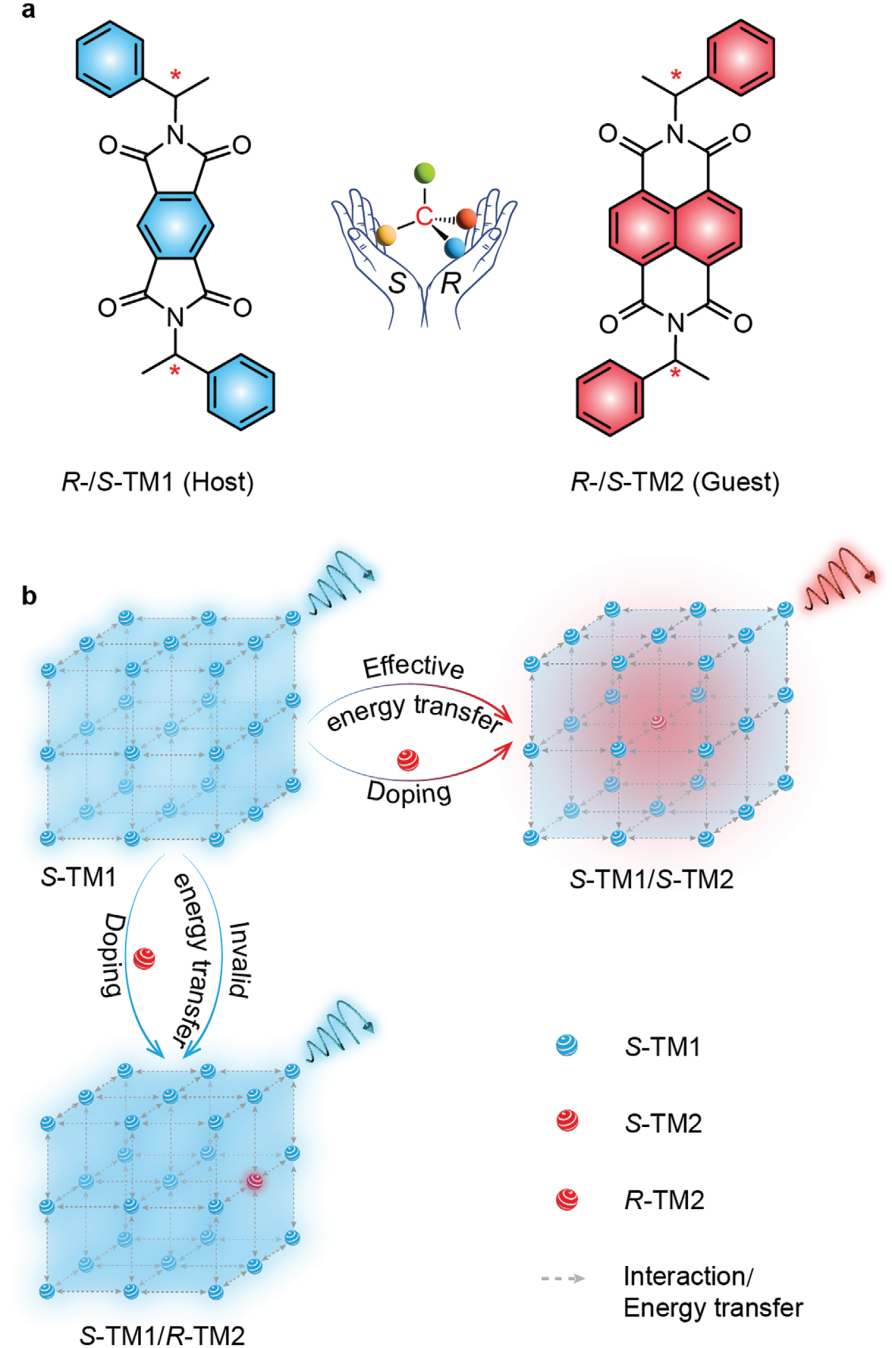
Schematic drawing of chiral selective room temperature phosphorescence (RTP) expression through host and guest doping. a) Molecular structure of chiral host and guest. b) Schematic diagram of the regulation of chiral‐dependent energy transfer for constructing a chiral recognition doping system with color‐tunable RTP emission. The identical chiral configurations of the host and guest molecules could empower efficient energy transfer due to the minimal spatial separation distance between host and guest molecules.

Inspired by the host‐guest doping strategy, we design a pair of chiral blue hosts, *R*‐/*S*‐TM1, and red guest, *R*‐/*S*‐TM2, with similar structures to manipulate chiral‐dependent energy transfer for constructing a chiral recognition doping system with color‐tunable RTP emission (**Figure** **1**a). In this design, because of the presence of carbonyl groups and heteroatoms in the TM1 molecule, the intersystem crossing (ISC) process is largely enhanced, which can effectively promote the generation of triplet excitons.^[^ ^16^ ^]^ Consequently, the *R*‐/*S*‐TM1 is employed as the host to serve as a triplet exciton tank for boosting RTP emission of *R*‐/*S*‐TM2 guest through modulating the interaction between TM1 and TM2 to trigger the chiral‐dependent energy transfer process (Figure 1b). By adjusting the chiral configuration of TM1 and TM2, the chiral‐dependent energy transfer efficiency can be modulated, which stimulates chiral selective expression of RTP emission.^[^ ^17^ ^]^ When TM1 and TM2 have identical chirality, red RTP emission from the guest, showing a lifetime of 32.7 ms and an emission peak at 612 nm, is achieved (Figure 1b, top). In contrast, when TM1 and TM2 have different chirality, blue RTP emission from TM1 host is retained due to ineffective energy transfer induced by an invalid host‐guest interaction (Figure 1b, bottom). This work not only provides a feasible way to recognize the configuration of chiral molecules through chiral‐dependent energy transfer but also expands the domains of RTP applications.

## 2 Results and Discussion

### 2.1 Synthesis and Characterization

Through a one‐step nucleophilic acylation reaction, chiral α‐methylbenzylamine (*R*‐/*S*‐MBA) is separately introduced into pyromellitic dianhydride (PMDI) and 1,4,5,8‐naphthalenetetracarboxylic dianhydride (NTDI) to prepare the chiral host (*R*‐/*S*‐TM1) and guest (*R*‐/*S*‐TM2) (Schemes S1 and S2, Supporting Information). Their structures were determined by ^1^H and ^13^C nuclear magnetic resonance (NMR) spectra, high‐resolution mass spectra (Figures S1–S12, Supporting Information), and single‐crystal X‐ray diffraction (SC‐XRD) analyses (Table S1, Supporting Information). The chiral purities are confirmed by the high‐performance liquid chromatography (HPLC) spectra. The enantiomeric excess (*ee*) values are calculated to be 100% and 100% for *S*‐TM1 and *R*‐TM1 as well as 99.972% and 99.992% for *S*‐TM2 and *R*‐TM2 (Figures S13 and S14, Supporting Information). The peaks at 7.5 min for *rac*‐TM1 and 7.1 min for *rac*‐TM2 are attributed to their corresponding meso‐compound.^[^ ^18^ ^]^ The same monoclinic, space group (*P‐21*), and similar dihedral angles between the phenyl and imide planes (111.19° and 113.79°) are found in SC‐XRD of TM1 and TM2 with the same chirality, revealing quite similar geometric structure in the crystalline state (Figure S15, Supporting Information), which could facilitate intense molecular packing and a short distance between the chiral host of TM1 and guest of TM2.

### 2.2 Photophysical Properties

Both TM1 and TM2 exhibit n‐π^*^ transition absorption at 320 nm (TM1) and 380 nm (TM2), as well as photoluminescence at 345 nm (TM1) and 398 nm (TM2) in THF solution (Figure S16, Supporting Information). TM1 and TM2 crystals exhibit fluorescence emission at 420 and 556 nm, with lifetimes of 0.28 and 8.63 ns, respectively (Figures S17–S20 and Table S4, Supporting Information). Interestingly, the steady‐state photoluminescence (SSPL) and delayed PL spectra of TM1 display a main peak at 484 nm with a lifetime of 17.50 ms (Figures S19 and S21, Supporting Information), suggesting its RTP‐dominated emission behavior. The RTP property of TM1 is further confirmed by the enhanced luminescent intensities at cryogenic temperature (Figure S22, Supporting Information). This result indicates that TM1 could be a promising candidate as a triplet exciton sensitizer for energy transfer.^[^ ^19^ ^]^ It can be observed that the absorption and excitation spectra of TM2 overlap significantly with the SSPL spectrum in THF solution and delayed PL in crystal of TM1 (Figure S23, Supporting Information), indicating that the host (TM1) and guest (TM2) doping system is expected to achieve energy transfer.^[^ ^20^ ^]^

**Figure 2:**
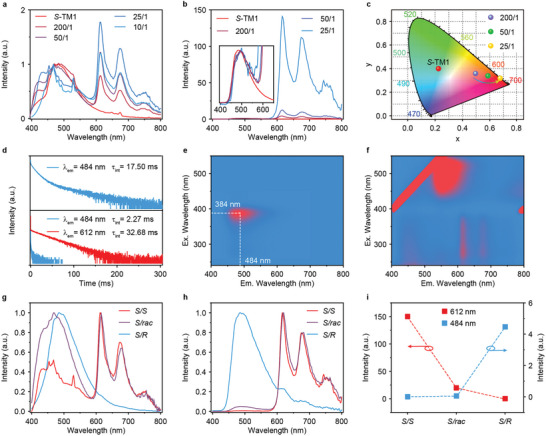
Photophysical properties investigation. a–c) Steady‐state photoluminescence (SSPL, a), delayed PL b) spectra and Commission Internationale de l'Eclairage (CIE) 1931 coordinates of delayed PL spectra c) of *S*‐TM2 (guest) doped *S*‐TM1 (host) (*S*‐TM1/*S*‐TM2) crystals with varied doping ratios. d) RTP decay profiles of *S*‐TM1 (top panel) and *S*‐TM1/*S*‐TM2 (bottom panel) crystals. e) Excitation‐delayed PL emission mapping of *S*‐TM1 crystal with a delayed time of 25 ms. f) Excitation‐SSPL emission mapping of *S*‐TM1/*S*‐TM2 crystals. g–i) SSPL g) and delayed PL h) spectra and RTP intensities at emission peaks of 484 and 612 nm i) of *S*‐TM1/*S*‐TM2 (*S*/*S*), *S*‐TM1/*rac*‐TM2 (*S*/*rac*) and *S*‐TM1/*R*‐TM2 (*S*/*R*) with a doping ratio of 25/1 under ambient conditions.

Experimentally, compared to *S*‐TM1 crystal showing blue emission at 484 nm (Figure S19, Supporting Information), the SSPL and delayed PL spectra of *S*‐TM2 (guest) doped *S*‐TM1 (host) (*S*‐TM1/*S*‐TM2) crystals with varied doping ratios exhibit obvious pink and red emissions with newly emerged peaks at 612 nm (**Figures** **2**a,b and S24–S26, Supporting Information), respectively. Additionally, with increasing doping ratios of *S*‐TM2, the luminescent intensities and lifetimes of emission peak at 484 nm gradually decrease, while the luminescent intensity at 612 nm gradually increases, leading to tunable emission colors (Figures 2a–d and S27, Supporting Information). However, with a further increase in the doping ratio of *S*‐TM2, the luminescent intensity from *S*‐TM2 (612 nm) decreases in the 10/1 doping system (Figure 2a). The low‐temperature delayed PL spectrum of *S*‐TM2 demonstrates an emission peak at 612 nm (Figure S28, Supporting Information), suggesting that the luminescent sources of peaks at 550 and 612 nm in *S*‐TM1/*S*‐TM2 crystals correspond to the fluorescence and phosphorescence of *S*‐TM2, respectively (Figure S20, Supporting Information). These results verify the possible occurrence of the nonradiative energy transfer from *S*‐TM1 to *S*‐TM2, achieving an optimized doping ratio of 25/1. The maximum energy transfer efficiency is calculated to be 79.55% based on the amplitude lifetime (Table S4, Supporting Information). Additionally, the observed red RTP emission of *S*‐TM1/*S*‐TM2 crystals can only be excited within the excitation range of TM1 (Figures 2e,f and S29 and S30, Supporting Information), while no RTP emission (612 nm) is observed when the excitation exceeds the absorption band of TM1 crystal (Figures 2f and S29, Supporting Information). Similar photophysical properties are also achieved in *R*‐TM1/*R*‐TM2 crystals (Figures S31–S33, Supporting Information). These profiles further confirm the energy transfer process in *S*‐TM1/*S*‐TM2 and *R*‐TM1/*R*‐TM2 crystals. Notably, the RTP intensities (Figures 2g–i and S34–S37, Supporting Information) and lifetimes (Figures S38–S40, Supporting Information) from TM2 decrease significantly when the TM2 guest and TM1 host have different chiral configurations. These results reveal the chiral configuration‐dependent energy transfer to achieve an alternative platform for enabling chirality recognition.

**Figure 3:**
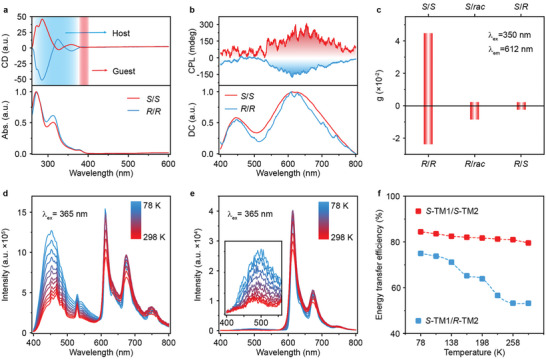
Chiral properties and temperature‐dependent properties of TM1/TM2 systems with a doping ratio of 25/1. a) Circular dichroic (CD) spectra of *S*‐TM1/*S*‐TM2 (*S*/*S*) and *R*‐TM1/*R*‐TM2 (*R*/*R*) in THF (≈10^−3^ m) solution. b) Circularly polarized luminescence (CPL) spectra of the *S*‐TM1/*S*‐TM2 and *R*‐TM1/*R*‐TM2 crystals. c) dissymmetry factor (*g*_lum_) values of TM2 guest doped *S‐* and *R*‐TM1 crystal. d–f) Temperature‐dependent SSPL d) and delayed PL e) spectra and energy transfer efficiencies f) of *S*‐TM1/*S*‐TM2 and *S*‐TM1/*R*‐TM2 crystals.

The chiral luminescence properties were systematically investigated to figure out the influence of energy transfer on chirality in the doped system. Circular dichroism (CD) spectra of TM1 and TM2 exhibit mirrored curves with a prominent Cotton effect in tetrahydrofuran (THF) solution (Figure S41, Supporting Information); and the doped *S*‐TM1/*S*‐TM2 and *R*‐TM1/*R*‐TM2 in THF solutions show almost identical CD curves to their corresponding chiral chromophore solutions (**Figures** **3**a and S42, Supporting Information). These results suggest that energy transfer has a limited influence on the CD properties. Interestingly, compared to *R*‐/*S*‐TM2 crystals showing limited circularly polarized luminescence (CPL) signals (Figure S44, Supporting Information), remarkable CPL signals consistent with those of *R*‐/*S*‐TM2 crystals have been observed in the doped *S*‐TM1/*S*‐TM2 and *R*‐TM1/*R*‐TM2 crystals (Figure 3b; Table S4, Supporting Information), respectively; and their dissymmetry factor (*g*_lum_) are 4.47 × 10^−2^ (612 nm) for *S*‐TM1/*S*‐TM2 and −2.38 × 10^−2^ (612 nm) for *R*‐TM1/*R*‐TM2 crystals. The larger *g*_lum_ of *S*‐TM1/*S*‐TM2 and *R*‐TM1/*R*‐TM2 may be attributed to their corresponding higher CPL intensity (Figure 3b). Notably, reduced CPL intensities from TM2 are achieved when the chiral TM1 crystal is doped with TM2 guest with opposite chiral configuration (*S*‐TM1/*R*‐TM2) (Figures 3c and S43–S47 and Table S4, Supporting Information), suggesting again the chiral configuration play an important role in stimulating chiral selective energy transfer from TM1 to TM2.

Interestingly, compared to the lifetime of TM1 host (17.5 ms, 484 nm), an elongated lifetime of 32.7 ms at 612 nm is observed in *S*‐TM1/*S*‐TM2 (25/1) crystals (Figures S21 and S38 and Table S4, Supporting Information). This suggests that TM1 host not only acts as triplet excitons tank for energy transfer but also constructs a rigid surrounding environment to suppress the non‐radiative decay of triplet excitons of *S*‐TM2 guest. Almost identical SSPL and delayed PL spectra are found in *S*‐TM1/*S*‐TM2 crystals under both ambient and vacuum atmospheres. In contrast, the intensity of SSPL and delayed PL spectra of *S*‐TM1/*R*‐TM2 crystals is stronger in vacuum than these in ambient atmospheres (Figures S48 and S49, Supporting Information). Moreover, as revealed by the time dependent density functional theoretical (TD‐DFT) calculations, both *S*‐TM1/*S*‐TM2 and *R*‐TM1/*R*‐TM2 doped crystals exhibit much higher binding energy and shorter hydrogen bonds compared to *S*‐TM1/*R*‐TM2 and *R*‐TM1/*S*‐TM2 doped crystals (Figures S58 and S59, Supporting Information). These results verify that the intermolecular interaction in *S*‐TM1/*S*‐TM2 crystals is much stronger than those in *S*‐TM1/*R*‐TM2 crystals, which should play a key point in boosting the RTP emission of *S*‐TM2. Meanwhile, these strong intermolecular interaction between *S*‐TM1 and *S*‐TM2 also confer an enhanced energy transfer process compared to the doped crystals system with the opposite chiral configuration (*S*‐TM1/*R*‐TM2). To further investigate the influence of temperature on the energy transfer from TM1 to TM2 with identical (*S*‐TM1/*S*‐TM2) or opposite (*S*‐TM1/*R*‐TM2) configuration, the temperature‐dependent SSPL and delayed PL spectra of *S*‐TM1/*S*‐TM2 and *S*‐TM1/*R*‐TM2 crystals were conducted. As shown in Figures 3d,e, both the intensities of SSPL and delayed PL spectra are largely enhanced with the decrease of temperature. This suggests the suppression of non‐radiative decay of triplet excitons. The energy transfer efficiencies calculated from the amplitude lifetime (Figures S51 and S52, Supporting Information) remain relatively stable at varied temperatures, ranging from 79.55% to 84.41% (Figure 3f; Table S5, Supporting Information). This demonstrates again the more rigid environment in *S*‐TM1/*S*‐TM2 crystals to suppress the molecule thermal vibration. Similarly, *S*‐TM1/*R*‐TM2 crystals also reveals the improved SSPL and delayed PL intensities (Figure S50, Supporting Information). In contrast, according to the amplitude lifetimes (Figures S51 and S53, Supporting Information), the energy transfer efficiencies of *S*‐TM1/*R*‐TM2 are lower than those of *S*‐TM1/*S*‐TM2 crystals (Figure 3f). There results indicate that the distance‐dependent triplet‐triplet Dexter energy transfer should be responsible for this interesting chiral dependent RTP emission.

### 2.3 Transient Absorption Analysis

**Figure 4:**
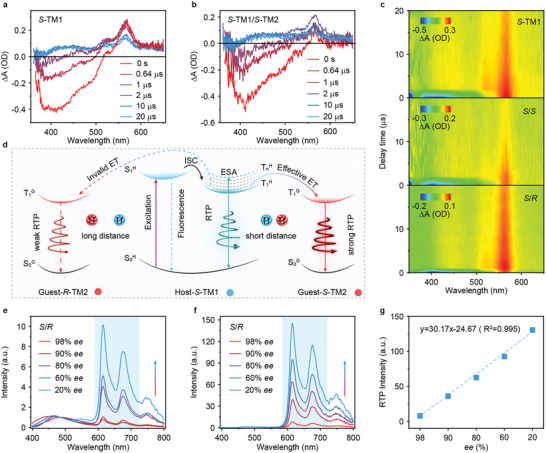
Possible mechanism and application of Chiral recognition. a,b) Time dependent transient absorption (TA) spectra of *S*‐TM1 a) and *S*‐TM1/*S*‐TM2 b) crystals (25/1) at the delay time range of 0–20 µs. c) 2D TA color maps of *S*‐TM1, *S*‐TM1*/S*‐TM2 (*S/S*) and *S*‐TM1*/R*‐TM2 (*S/R*) at the delay time range of 0–20 µs. d) Schematic diagram of chirality recognition energy transfer mechanism in the doping systems. e,f) SSPL e) and delayed PL f) spectra of *S*‐TM1/*R*‐TM2 (25/1) crystals by doping *S*‐TM2 with varied *ee* values. g) Variations of RTP intensities at 612 nm of *S*‐TM1/*R*‐TM2 (25/1) crystals by doping *S‐*TM2 with varied *ee* values.

To gain a deep understanding of the Dexter energy transfer mechanism, nanosecond transient absorption (TA) spectra were conducted on *S*‐TM1/*S*‐TM2 (25/1), *S*‐TM1/*R*‐TM2 (25/1) and *S*‐TM1 crystals. Compared to the SSPL spectra of TM1 crystal, the negative TA peaks observed at 420 and 484 nm are attributed to stimulated emission (STE) of fluorescence and RTP emission from TM1 (**Figure** **4**a). Moreover, a long‐lived positive decay with a lifetime of 77.7 µs is found at 567 nm; the signal intensity basically remains unchanged at the time range from 0 to 2 µs and gradually decreases from 2 to 20 µs (Figure 4a). Therefore, this ultralong lifetime excited state absorption (ESA) peak should be assigned to the triplet excited state.^[^ ^21^ ^]^ Furthermore, a positive peak at 526 nm gradually increases at the time ranging from 0 to 2 µs and the signal intensity also keeps unchanged at the time ranging from 2 to 20 µs. Combined with the decreased ESA peak at 567 nm and luminescence attenuation in 350–500 nm, this peak at 526 nm can be attributed to ISC process of TM1,^[^ ^22^ ^]^ which explains well the RTP dominant emission of TM1, conferring an efficient triplet sensitizer. After doping with *S*‐TM2, both the TA signal at 526 and 567 nm show a significant decrease, showing a lifetime of 30.0 and 20.0 µs, respectively (Figure S54, Supporting Information). This may be due to the short distance for effective Dexter energy transfer to enable the triplet excitons transition from *S*‐TM1 to *S*‐TM2, which accelerates the ISC process and triplet excitons dissipation in *S*‐TM1 (Figures 4a,b). Notably, the opposite chiral configuration in the doping system may generate longer molecular distance than the same chiral configuration, resulting in invalid Dexter energy transfer and quite poor RTP emission of TM2 as confirmed by the limited variations in 2D TA color maps between *S*‐TM1 and *S*‐TM1/*R*‐TM2 (Figures 4c and S55, Supporting Information).

Based on experimental and theoretical calculations, a possible chiral‐dependent energy transfer mechanism (Figure 4d) is proposed. Because of the fast and effective ISC process in TM1 host, the triplet excitons are generated in TM1 and then stabilized by the molecular crystal (Figures S56 and S60, Supporting Information), which serves as the exciton tanks. With the aid of the Dexter energy transfer process, triplet excitons can be effectively transferred to the guest with appreciative host‐guest distance. When the TM1 host and TM2 guest have identical chirality, a short distance is achieved in the doped system, enabling effective Dexter energy transfer, i.e., *S*‐TM1 to *S*‐TM2 and *R*‐TM1 to *R*‐TM2. The dominant RTP properties from chiral TM2 guest are achieved. When the TM1 host and TM2 guest have different chirality, a relatively large distance is introduced in the doped system, resulting in invalid Dexter energy transfer efficiency, i.e., *R*‐TM1 to *S*‐TM2 and *S*‐TM1 to *R*‐TM2. This leads to the much decreased RTP emission from chiral TM2 guest.

### 2.4 Chiral Recognition

Benefiting from the chiral configuration‐dependent energy transfer process, the potential application of chirality recognition was investigated to determine the chiral enantiomeric excess (*ee*) compositions. The RTP properties of *S*‐TM1/*R*‐TM2 (25/1) crystals system were evaluated by doping *R*‐TM2 with varied *ee* values. The *ee* values of *R*‐TM2 guests could be modulated from 98% to 20% by mixing the *R*‐TM2 and *S*‐TM2 in varied weight concentrations from 0.5% to 40%. As the *ee* value of *R*‐TM2 decreases from 98% to 20%, an efficient energy transfer process is enabled, resulting in gradually enhanced fluorescence and RTP emission from *R*‐TM2 (Figures 4e,f). The RTP intensities peaking at 612 nm in *S*‐TM1/*R*‐TM2 crystals increase linearly (Figures 4g and S57, Supporting Information), thereby providing a facile but efficient way for high‐sensitivity chiral recognition through chiral selective energy transfer.

## 3 Conclusion

In summary, we have successfully designed and synthesized structurally similar chiral host (TM1) and guest (TM2) molecules. Chiral selective expression of RTP luminescence is evoked, which leans upon chiral configuration‐dependent Dexter energy transfer. When the chirality of TM1 and TM2 is identical, efficient energy transfer from TM1 to TM2 is achieved due to the short distance between TM1 and TM2, enabling red RTP emission from TM2 with an emission peak at 612 nm and a lifetime of 32.7 ms. Conversely, when the chiral configuration of TM1 and TM2 is opposite, the energy transfer is largely suppressed. Benefiting from this chirality‐dependent energy transfer process, a recognition application for monitoring the optical purity of chiral molecules is conceptually demonstrated. This work not only provides a possible explanation for the chiral‐dependent energy transfer mechanism but also offers a way to realize the chirality recognition through invoking efficient phosphorescence emission.

## 4 Experimental Section

Materials, instrumentation, and detailed experimental procedures can be found in the Supporting Information.

CCDC2223673, 2223674, 2282076, and 2282087 contain the supplementary crystallographic data for this paper. These data can be obtained free of charge from The Cambridge Crystallographic Data Centre via www.ccdc.cam.ac.uk/data_request/cif.

## Conflict of Interest

The authors declare no conflict of interest.

## Author Contributions

Z.G. synthesized and characterized the materials, and processed and plotted the experimental data. X.Y., Q.J., J.Z., and G.G. carried on the partially supplemented data and revised the manuscript. H.L., H.L., and G.X. provided helpful discussions on the synthesis and testing. R.C. and Y. T. supervised this work, wrote the manuscript, and are responsible for funding acquisitions.

## Supporting information



Supporting Information

## Data Availability

The data that support the findings of this study are available from the corresponding author upon reasonable request.
