# Supplementary material for: Stimulating Chiral Selective Expression of Room Temperature Phosphorescence for Chirality Recognition
Source: Adv Sci (Weinh). 2024 Oct 8;11(44):2410671. doi: 10.1002/advs.202410671 (PMC11600253; doi:10.1002/advs.202410671)
Supplement: Supplementary file 1 — Supporting Information [file ADVS-11-2410671-s001.docx]

Supporting Information

**Stimulating Chiral Selective Expression of Room Temperature Phosphorescence for Chirality Recognition**

*Zhisheng Gao, Xin Yan, Qi Jia, Jingru Zhang, Guangyao Guo, Huanhuan Li, Hui Li, Gaozhan Xie, Ye Tao*, Runfeng Chen*, and Wei Huang**

# 1. Synthesis and characterization

**Materials**

Chemical reagents, unless otherwise specified, were purchased from Energy Chemical, Acros, or Alfa Aesar, and used without further purification. Manipulations involving air-sensitive reagents were performed under an argon atmosphere.

**Instruments**

^1^H and ^13^C-nuclear magnetic resonance (NMR) spectra were recorded on a Bruker Ultra Shield Plus 400 MHz instrument with DMSO-*d_6_* as the solvent and tetramethyl silane (TMS) as the internal standard. A high-resolution mass spectra (HRMS) was obtained on an LCT Premier XE (Waters) HRMS spectrometry. High-performance liquid chromatogram (HPLC) spectra were collected by LC-40D spectrometry monitored with DCM-ethanol as eluent in volumetric ratio of 50: 50 (v/v) for 15 min at 0.5 mL/min.


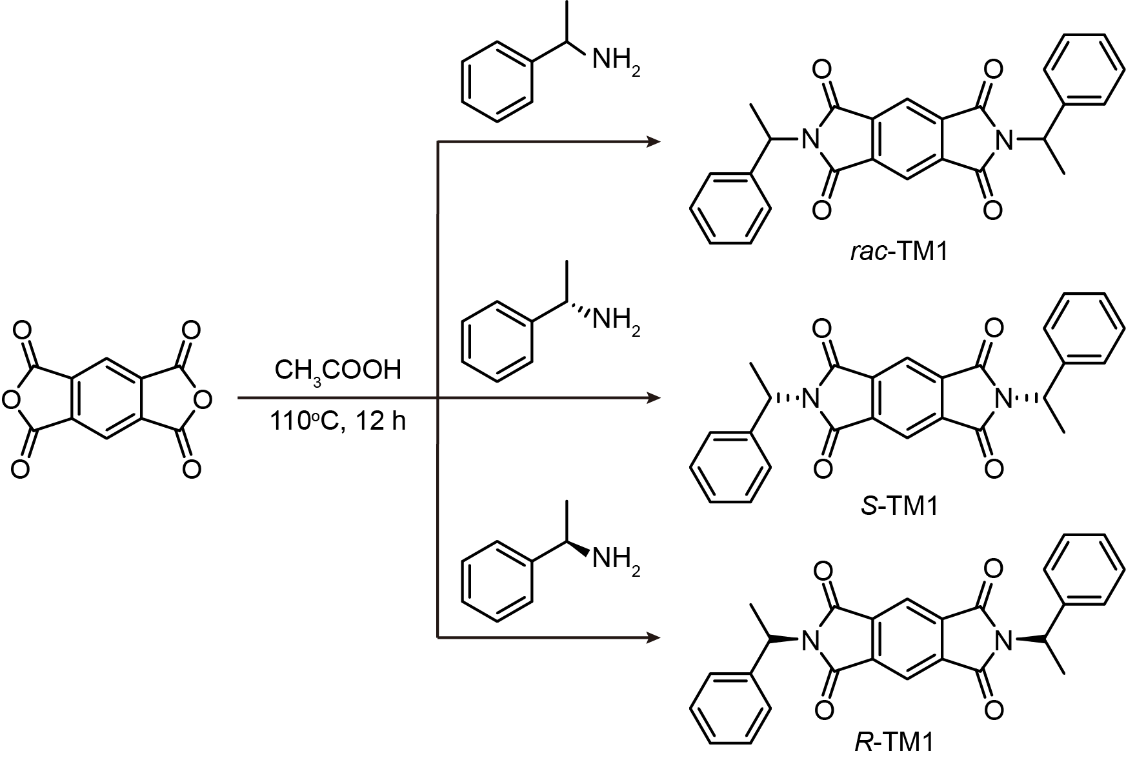


**Scheme S1**. Synthetic routes of the TM1.


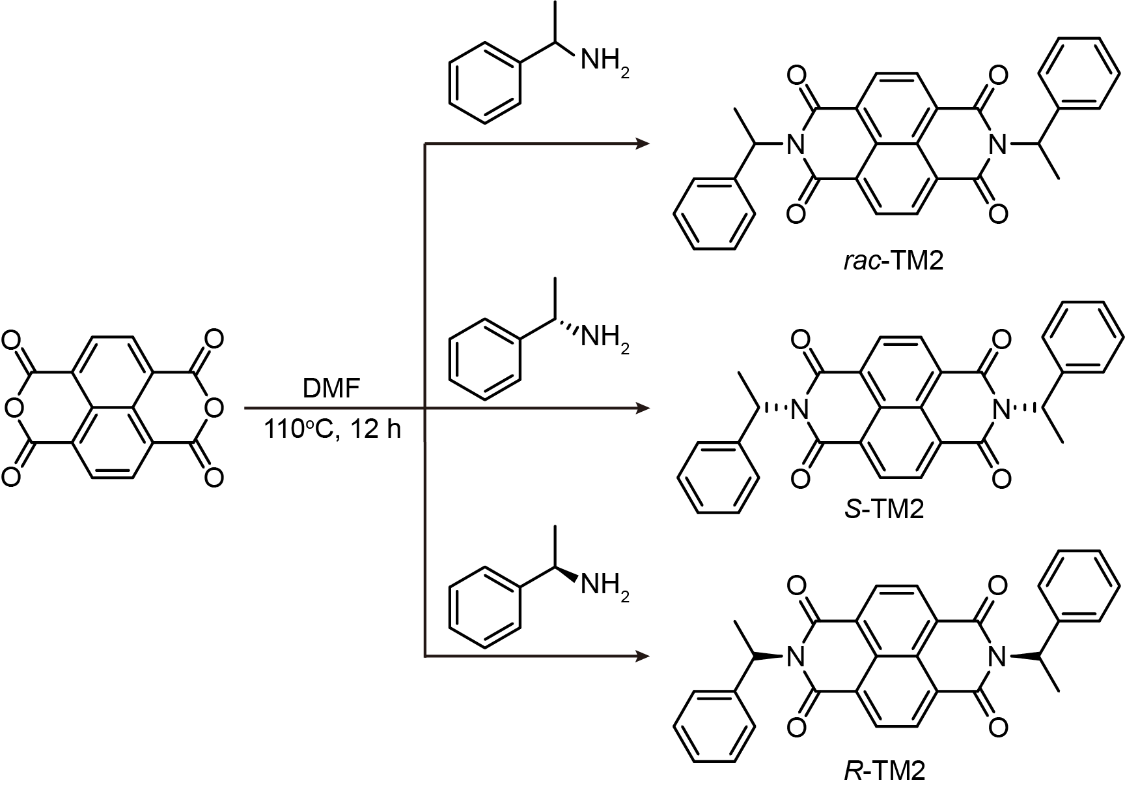


**Scheme S2**. Synthetic routes of the TM2.

**Synthesis of 2,6-bis((*S*)-1-phenylethyl)pyrrolo[3,4-f]isoindole-1,3,5,7(2H,6H)-tetraone (*S*-TM1)**

Pyromellitic dianhydride (PMDI) (1 g, 4.6 mmol) and acetic acid (50 mL) were added into a 100 mL round-bottomed flask, and stirred at room temperature until completely dissolved, and then *S*-α-methylbenzylamine (*S*-MBA) (1.5 mL, 11.5 mmol) was added. The reaction mixture was stirred at 110℃ for 12 hours. The mixture was cooled to room temperature, filtrated, and washed with water. The solid residue was purified by column chromatography (silica gel, dichloromethane (DCM)). Yield: 1.69 g as a white powder (86.3%). ^1^H NMR (400 MHz, DMSO-*d_6_*): δ 8.18 (s, 2H), δ 7.42 (d, *J* = 8 Hz, 4H), δ 7.35 (t, *J* = 8 Hz, 4H), δ 7.28 (t, *J* = 8 Hz, 2H), δ 5.51 (m, *J* = 8 Hz, 2H), δ 1.85 (d, *J* = 8 Hz, 6H). ^13^C NMR (100 MHz, DMSO-*d_6_*, ppm) δ 166.56, 140.56, 137.22, 128.95, 127.93, 127.15, 49.89, 17.90. HRMS (EI): m/z calcd for C_26_H_20_N_2_O_4_ [M+H]^+^: 425.2911; found: 425.1485.

**Synthesis of 2,6-bis((*R*)-1-phenylethyl)pyrrolo[3,4-f]isoindole-1,3,5,7(2H,6H)-tetraone (*R*-TM1)**

*R*-TM1 was prepared under the identical synthetic conditions described in the preparation of *S*-TM1 using PMDI (1 g, 4.6 mmol) and *R*-α-methylbenzylamine (*R*-MBA) (1.5 mL, 11.5 mmol). Yield: 1.66 g as a white powder (85.1%). ^1^H NMR (400 MHz, DMSO-*d_6_*): δ 8.18 (s, 2H), δ 7.41 (d, *J* = 8 Hz, 4H), δ 7.34 (t, *J* = 8 Hz, 4H), δ 7.27 (t, *J* = 8 Hz, 2H), δ 5.51 (m, *J* = 8 Hz, 2H), δ 1.85 (d, *J* = 8 Hz, 6H). ^13^C NMR (100 MHz, DMSO-*d*_6_, ppm) δ 166.56, 140.65, 137.22, 128.95, 127.93, 127.15, 49.89, 17.90. HRMS (EI): m/z calcd for C_26_H_20_N_2_O_4_ [M+H]^+^: 425.2911; found: 425.1486.

**Synthesis of 2,6-bis((*rac*)-1-phenylethyl)pyrrolo[3,4-f]isoindole-1,3,5,7(2H,6H)-tetraone (*rac*-TM1)**

*Rac*-TM1 was prepared under the identical synthetic conditions described in the preparation of *S***-**TM1 using PMDI (1 g, 4.6 mmol) and *rac*-α-methylbenzylamine (*rac*-MBA) (1.5 mL, 11.5 mmol). Yield: 1.75 g white powder (89.5%). ^1^H NMR (400 MHz, DMSO-*d_6_*): δ 8.18 (s, 2H), δ 7.42 (d, *J* = 8 Hz, 4H), δ 7.35 (t, *J* = 8 Hz, 4H), δ 7.28 (t, *J* = 8 Hz, 2H), δ 5.51 (m, *J* = 8 Hz, 2H), δ 1.86 (d, *J* = 8 Hz, 6H). ^13^C NMR (100 MHz, DMSO-*d_6_*, ppm) δ 166.56, 140.65, 137.21, 128.95, 127.93, 127.16, 49.88, 17.90. HRMS (EI): m/z calcd for C_26_H_20_N_2_O_4_ [M+H]^+^: 425.2911; found: 425.1485.

**Synthesis of 2,7-bis((*S*)-1-phenylethyl)benzo[lmn][3,8]phenanthroline-1,3,6,8(2H,7H)-tetraone (*S*-TM2)**

1,4,5,8-Naphthalenetetracarboxylic dianhydride (NTDI) (1 g, 3.7 mmol) and N, N-Dimethylformamide (DMF, 50 mL) was stirred until it was completely dissolved, and *S*-MBA (1.5 mL, 11.5 mmol) was added. The reaction mixture was stirred at 110℃ for 12 h. The reaction was quenched by 1000 mL water and extracted with DCM (3×200 mL). The organic layer was collected and dried with anhydrous sodium sulfate and purified by column chromatography. (silica gel, 1:3 v/v, petroleum ether (PE)/DCM) to obtain a pink powder. Yield: 1.32 g of pink powder (75.3%). ^1^H NMR (400 MHz, DMSO-*d_6_*): δ 8.64 (s, 4H), δ 7.41 (d, *J* = 8 Hz, 4H), δ 7.31 (t, *J* = 8 Hz, 4H), δ 7.22 (t, *J* = 8 Hz, 2H), δ 6.36 (m, *J* = 8 Hz, 2H), δ 1.92 (d, *J* = 8 Hz, 6H). ^13^C NMR (100 MHz, DMSO-*d_6_*, ppm) δ 163.16, 141.03, 131.11, 128.58, 127.11, 127.02, 126.90, 126.77, 50.35, 16.69. HRMS (EI): m/z calcd for C_30_H_22_N_2_O_4_ [M+H]^+^: 475.5077; found: 475.1638.

**Synthesis of 2,7-bis((*R*)-1-phenylethyl)benzo[lmn][3,8]phenanthroline-1,3,6,8(2H,7H)-tetraone (*R*-TM2)**

*R*-TM2 was prepared under the identical synthetic conditions described in the preparation of *S*-TM2 using NTDI (1 g, 3.7 mmol) and *R*-MBA (1.5 mL, 11.5 mmol). Yield: 1.41 g of pink powder (80.1%). ^1^H NMR (400 MHz, DMSO-*d*_6_): δ 8.64 (s, 4H), δ 7.41 (d, *J* = 8 Hz, 4H), δ 7.31 (t, *J* = 8 Hz, 4H), δ 7.23 (t, *J* = 8 Hz, 2H), δ 6.36 (m, *J* = 8 Hz, 2H), δ 1.92 (d, *J* = 8 Hz, 6H). ^13^C NMR (100 MHz, DMSO-*d_6_*, ppm) δ 163.16, 141.03, 131.11, 128.58, 127.11, 127.02, 126.90, 126.77, 50.35, 16.70. HRMS (EI): m/z calcd for C_30_H_22_N_2_O_4_ [M+H]^+^: 475.5077; found: 475.1637.

**Synthesis of 2,7-bis((*rac*)-1-phenylethyl)benzo[lmn][3,8]phenanthroline-1,3,6,8(2H,7H)-tetraone (*rac*-TM2)**

*Rac*-TM2 was prepared under the identical synthetic conditions described in the preparation of *S*-TM2 using NTDI (1 g, 3.7 mmol) and *rac*-MBA (1.5 mL, 11.5 mmol). Yield: 1.38 g of pink powder (78.6%). ^1^H NMR (400 MHz, DMSO-*d_6_*): δ 8.65 (s, 4H), δ 7.42 (d, *J* = 8 Hz, 4H), δ 7.32 (t, *J* = 8 Hz, 4H), δ 7.23 (t, *J* = 8 Hz, 2H), δ 6.37 (m, *J* = 8 Hz, 2H), δ 1.92 (d, *J* = 8 Hz, 6H). ^13^C NMR (100 MHz, DMSO-*d*_6_, ppm) δ 163.02, 140.68, 131.03, 128.36, 127.13, 127.03, 126.94, 126.73, 50.37, 16.41. HRMS (EI): m/z calcd for C_30_H_22_N_2_O_4_ [M+H]^+^: 475.5077; found: 475.1643.

**
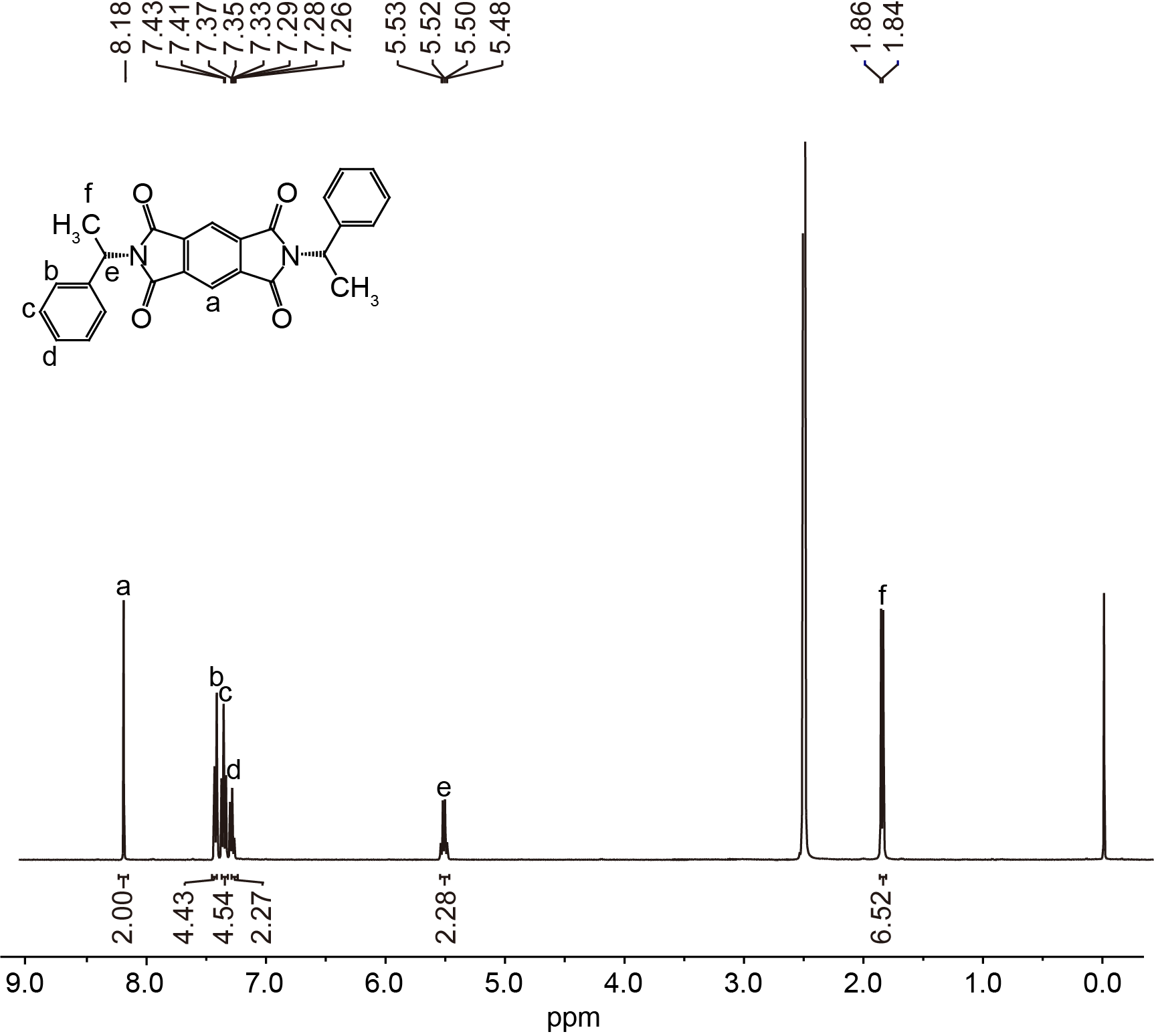
**

**Figure S1.** ^1^H NMR spectrum of *S*-TM1.


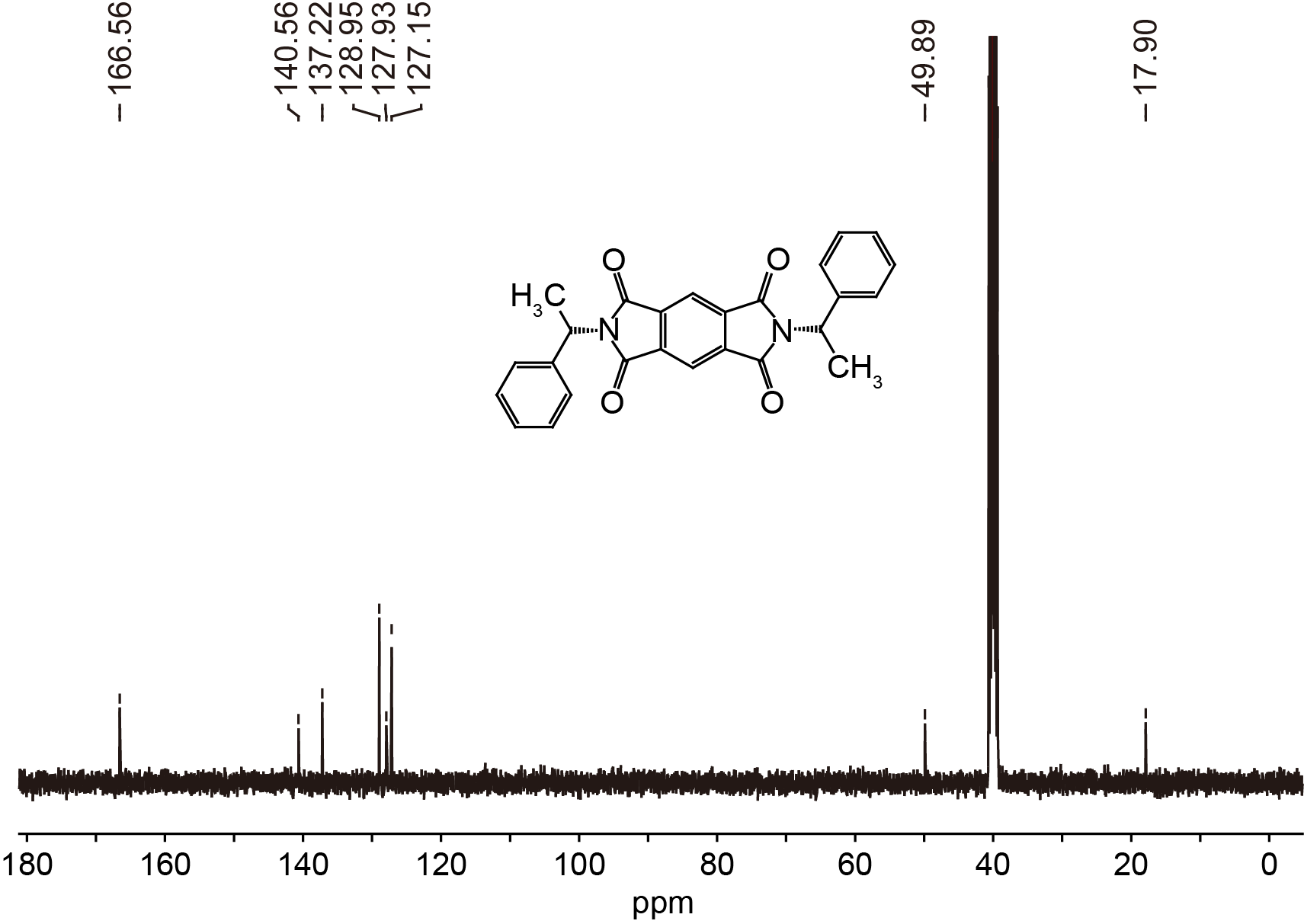


**Figure S2.** ^13^C NMR spectrum of *S*-TM1.

**
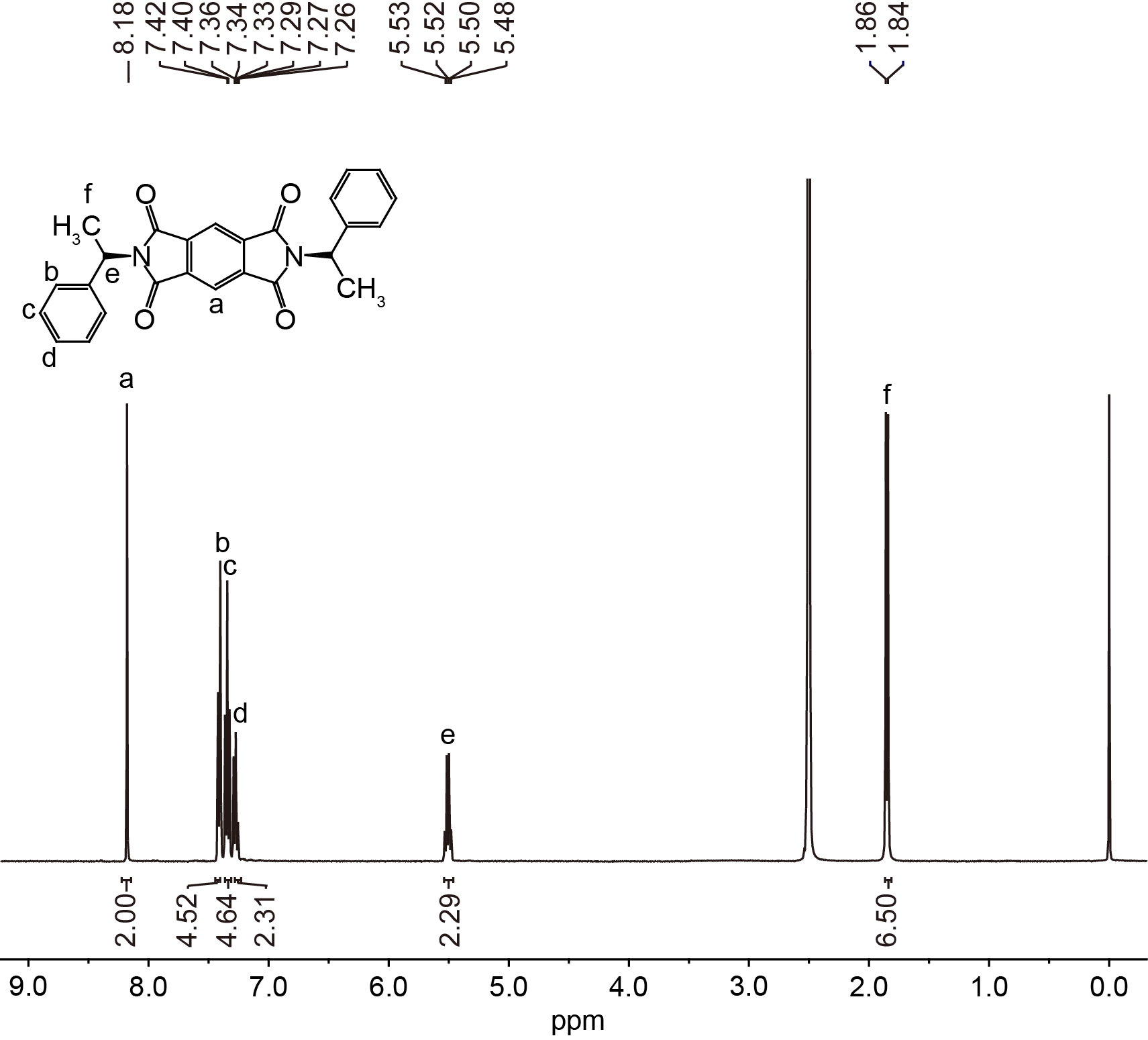
**

**Figure S3.** ^1^H NMR spectrum of *R*-TM1.


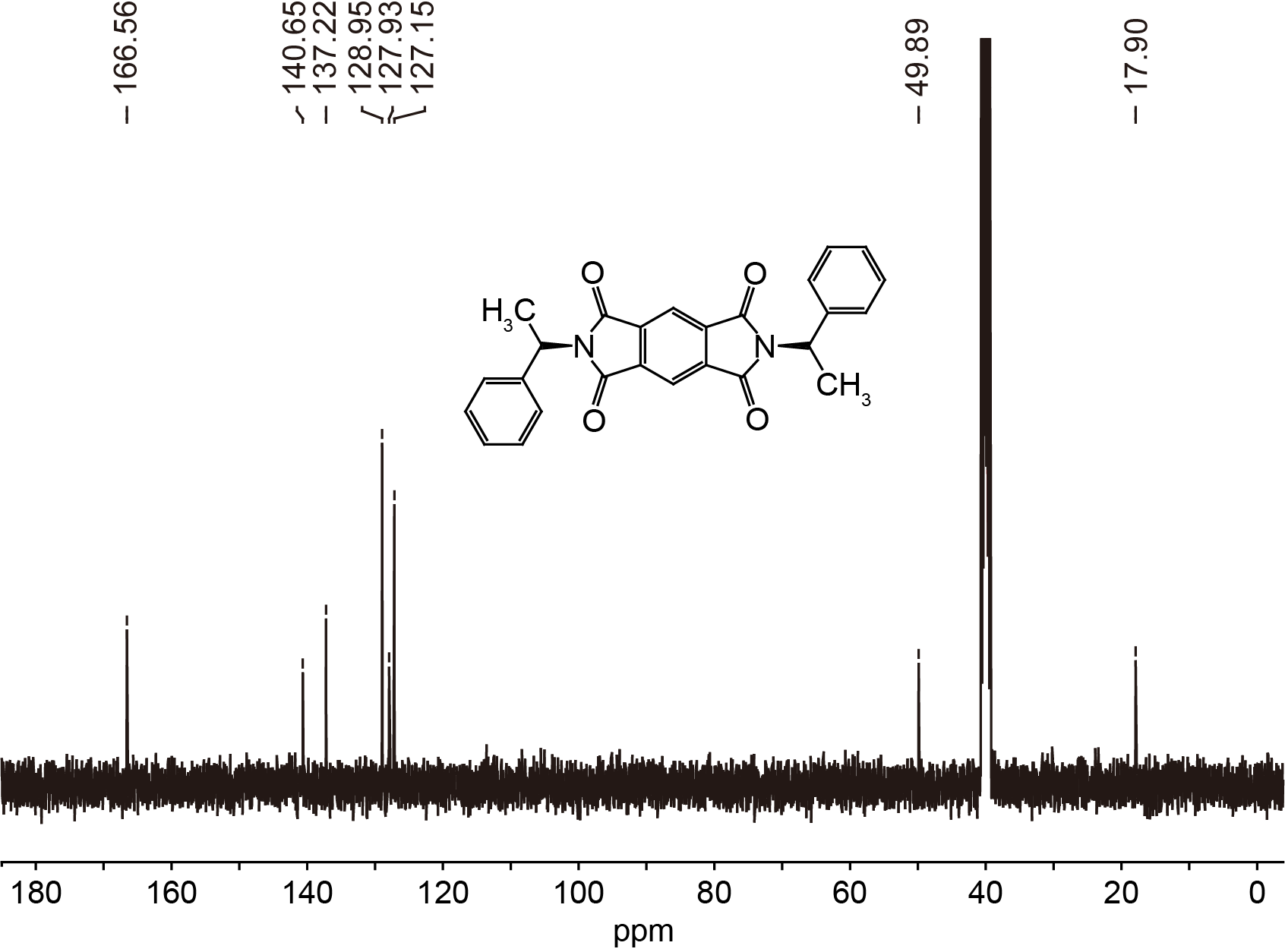


**Figure S4.** ^13^C NMR spectrum of *R*-TM1.


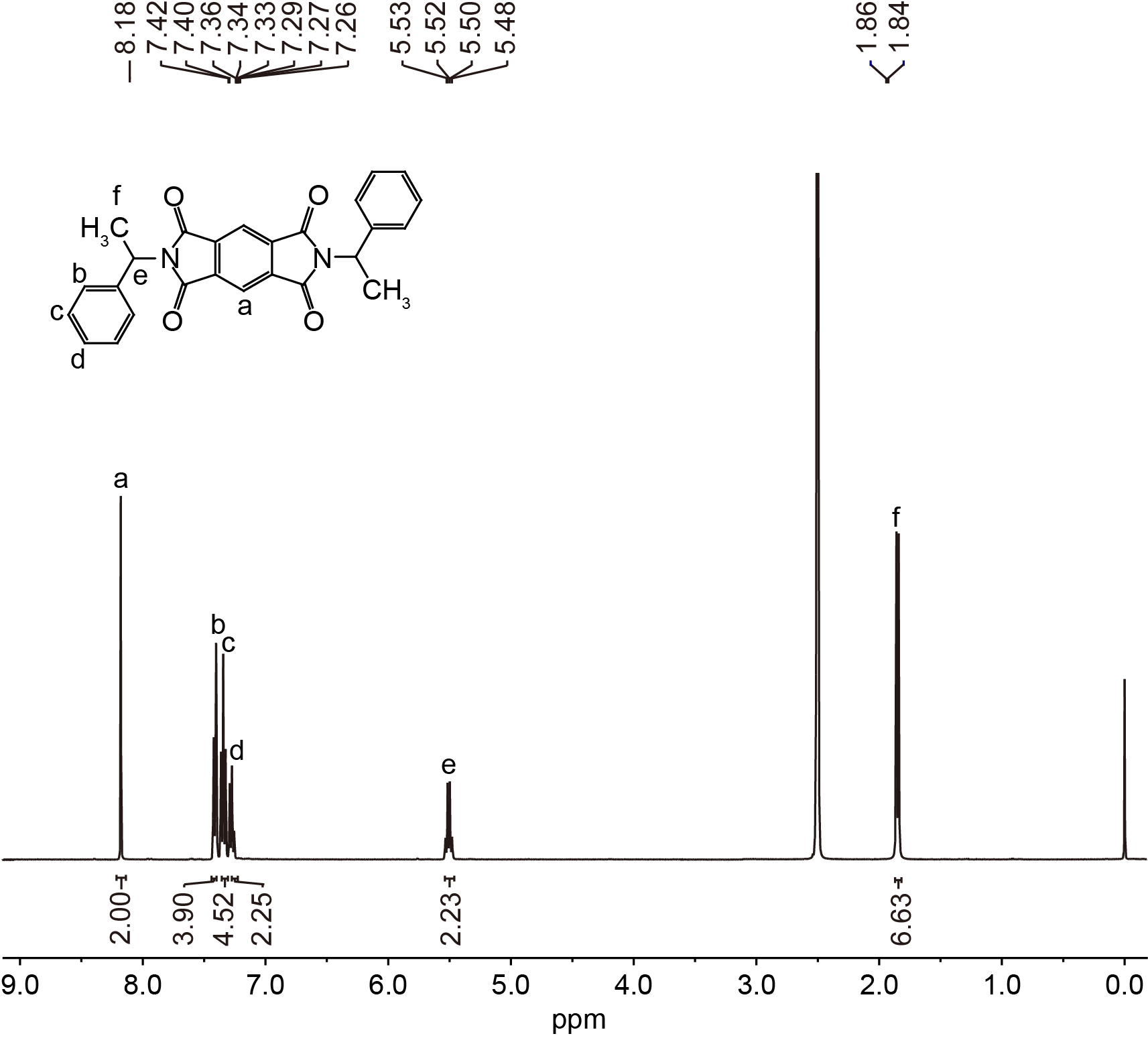


**Figure S5**. ^1^H NMR spectrum of *rac*-TM1.


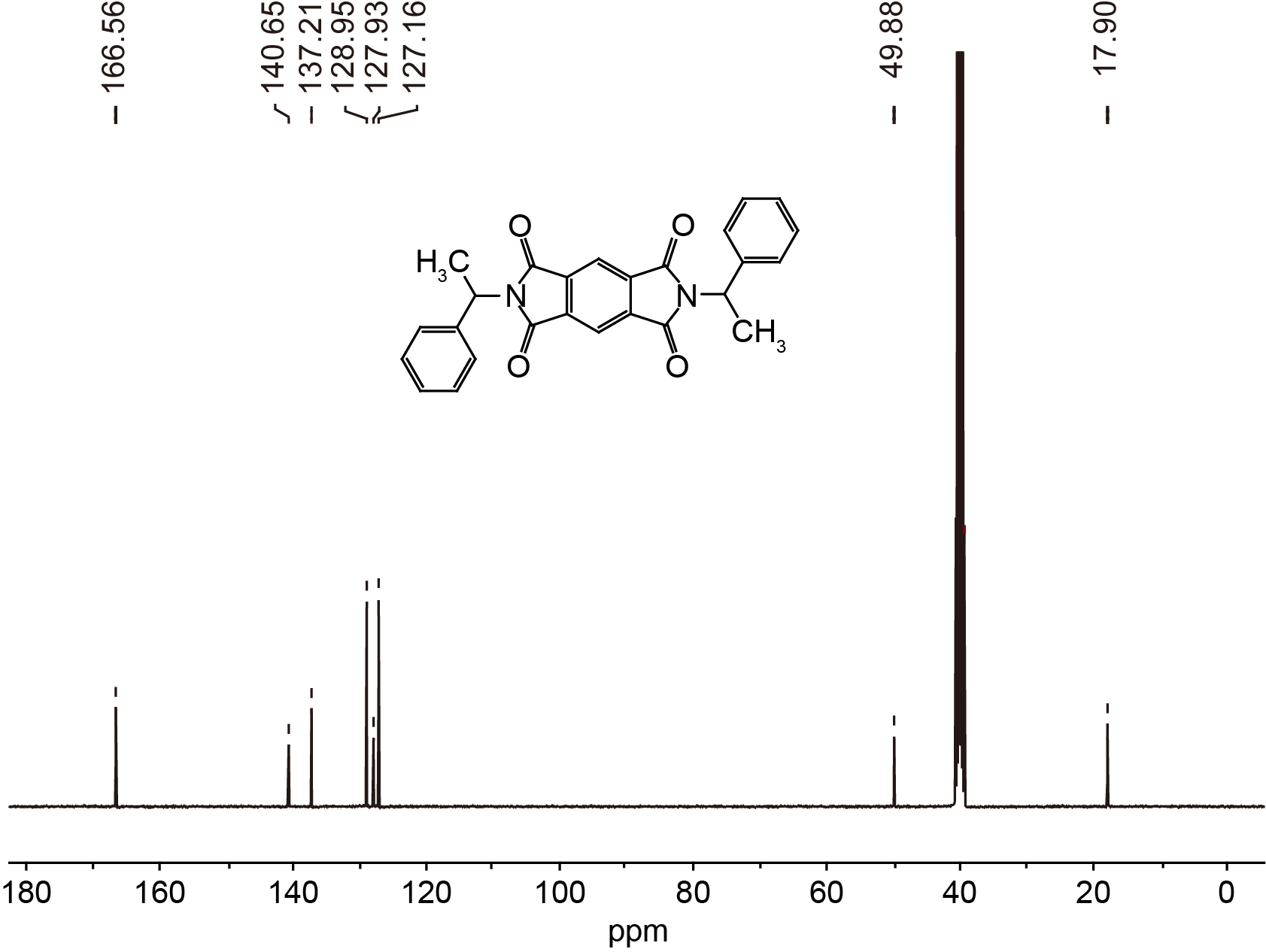


**Figure S6**. ^13^C NMR spectrum of *rac*-TM1.


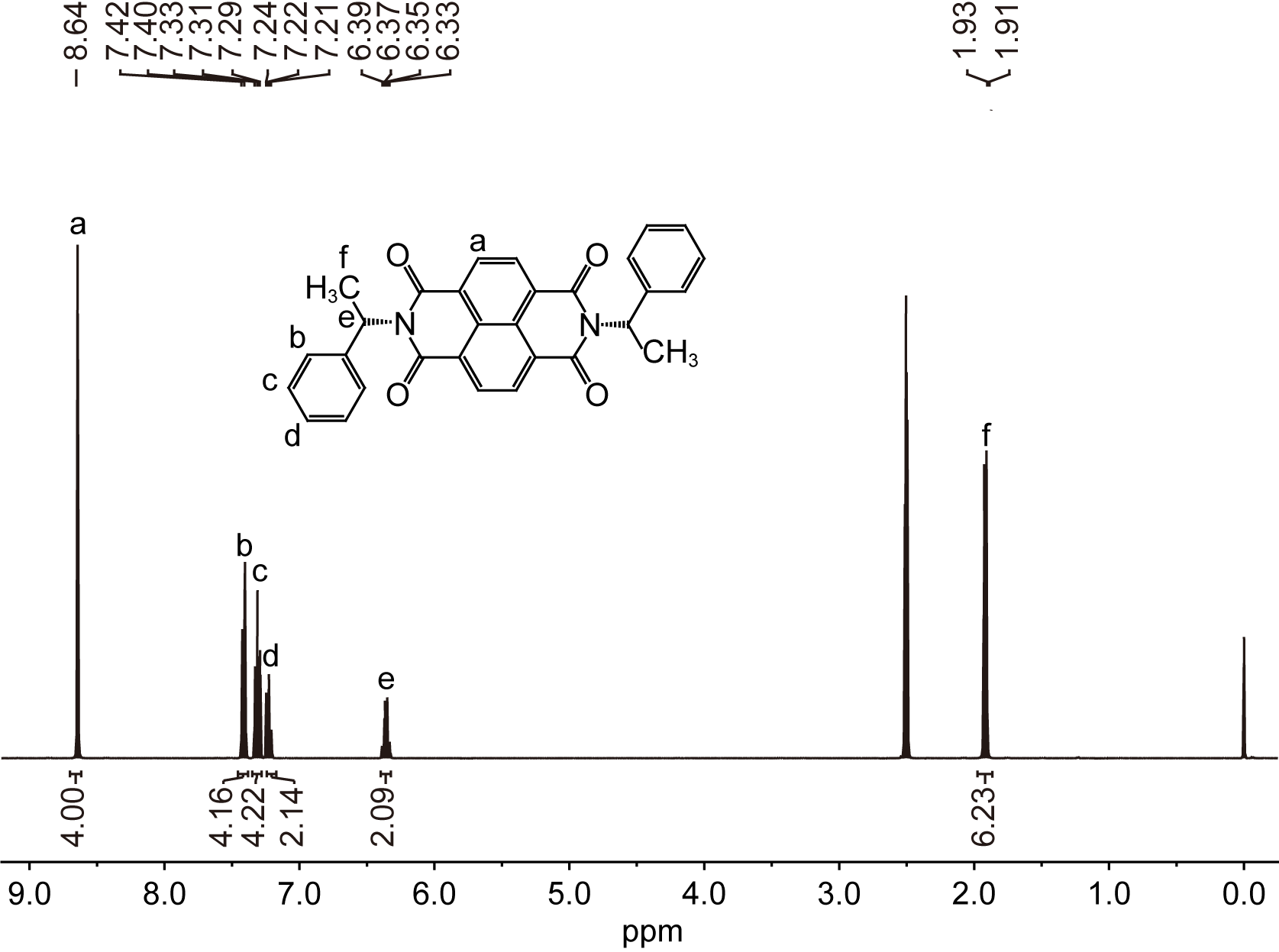


**Figure S7**. ^1^H NMR spectrum of *S*-TM2.


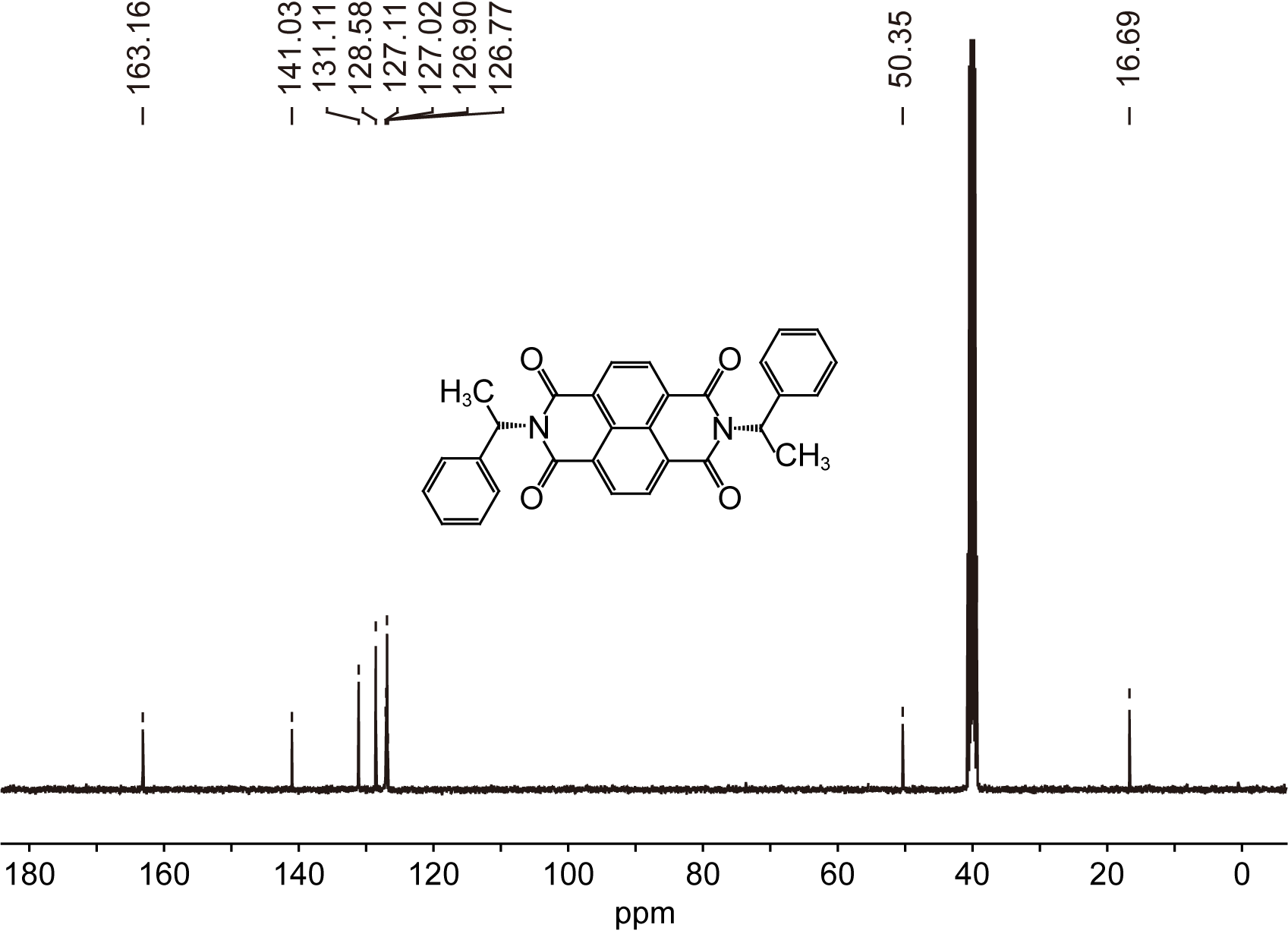


**Figure S8**. ^13^C NMR spectrum of *S*-TM2.


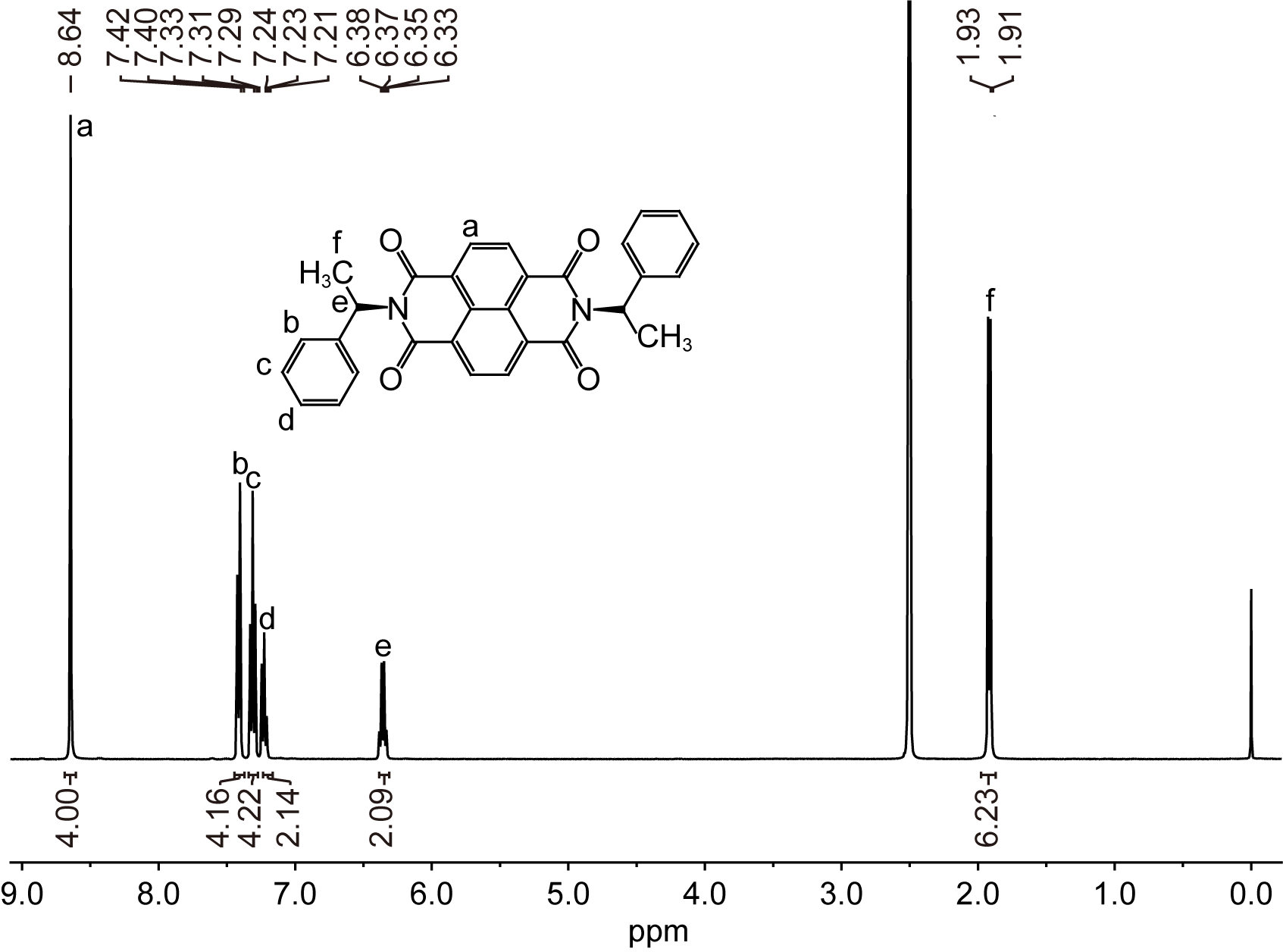


**Figure S9**. ^1^H NMR spectrum of *R*-TM2.


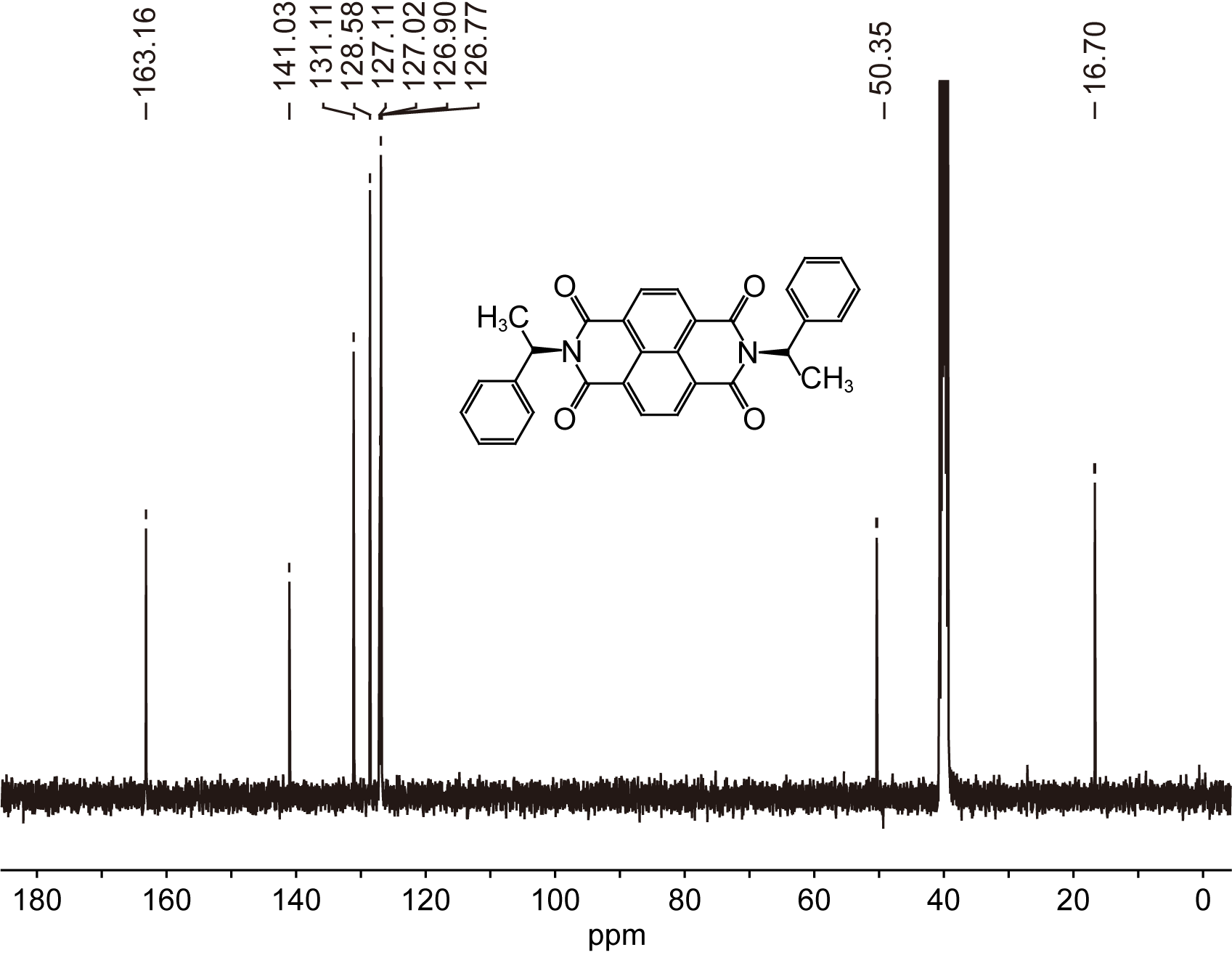


**Figure S10**. ^13^C NMR spectrum of *R*-TM2.

**
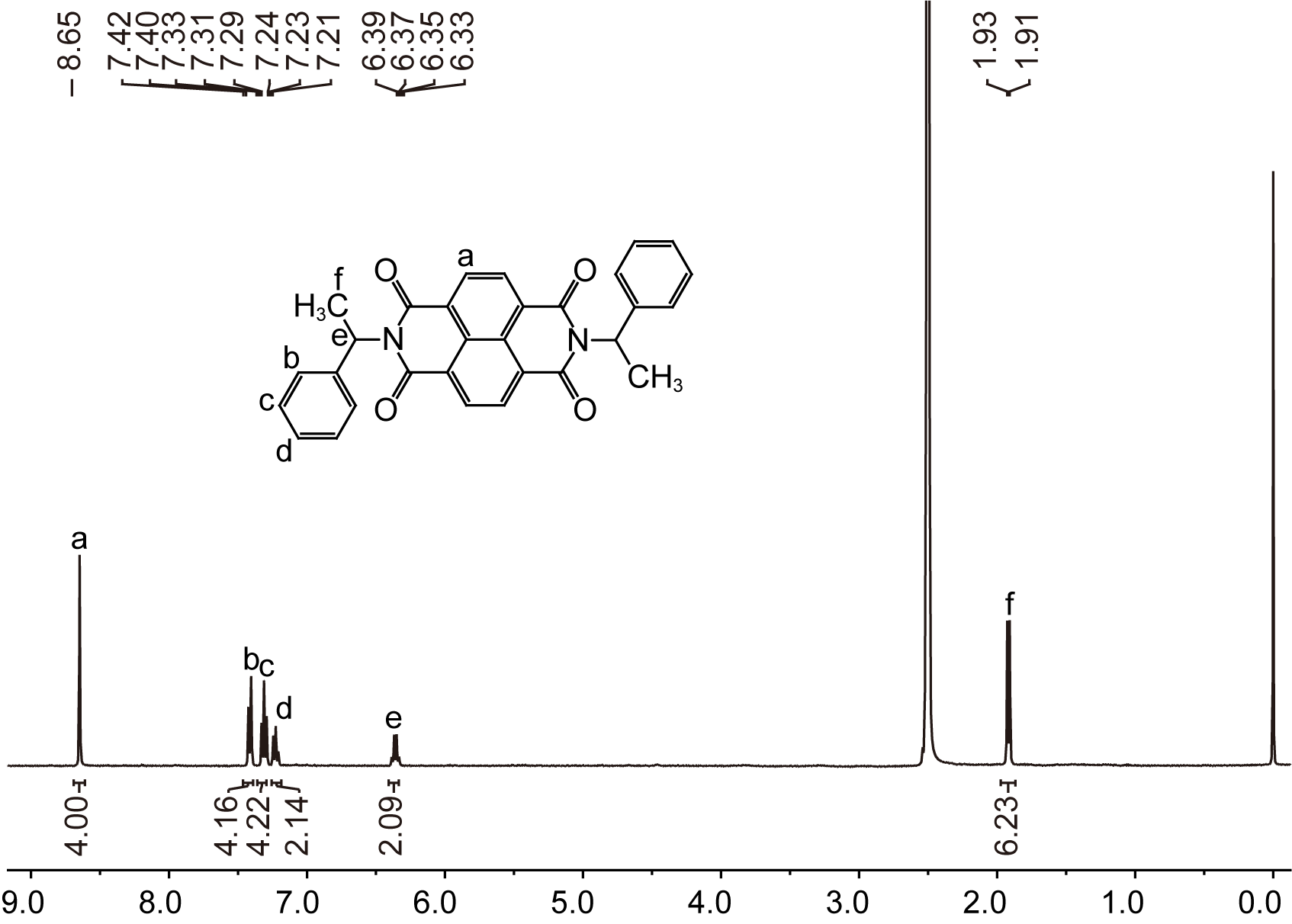
**

**Figure S11**. ^1^H NMR spectrum of *rac*-TM2.


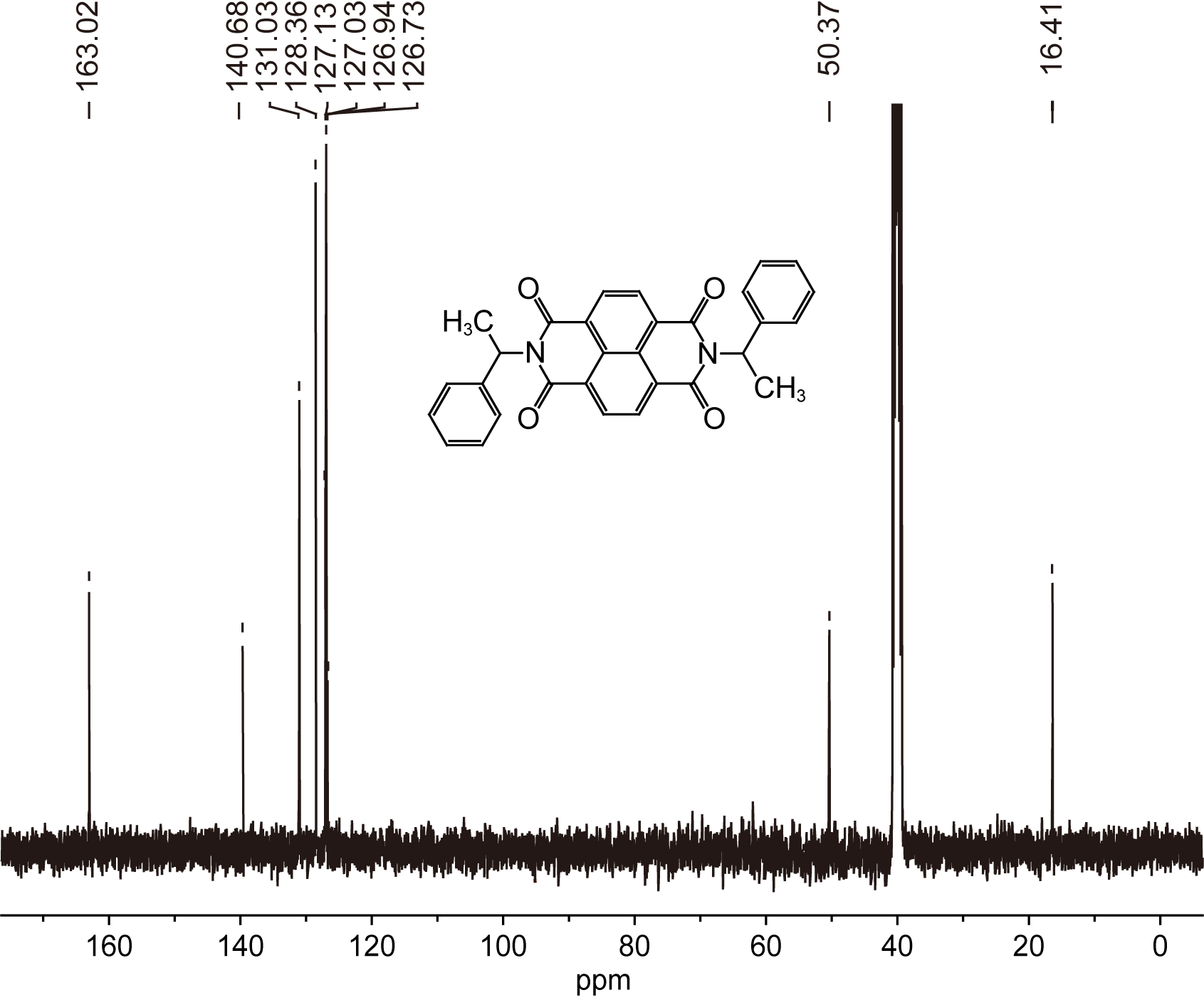


**Figure S12**. ^13^C NMR spectrum of *rac*-TM2.


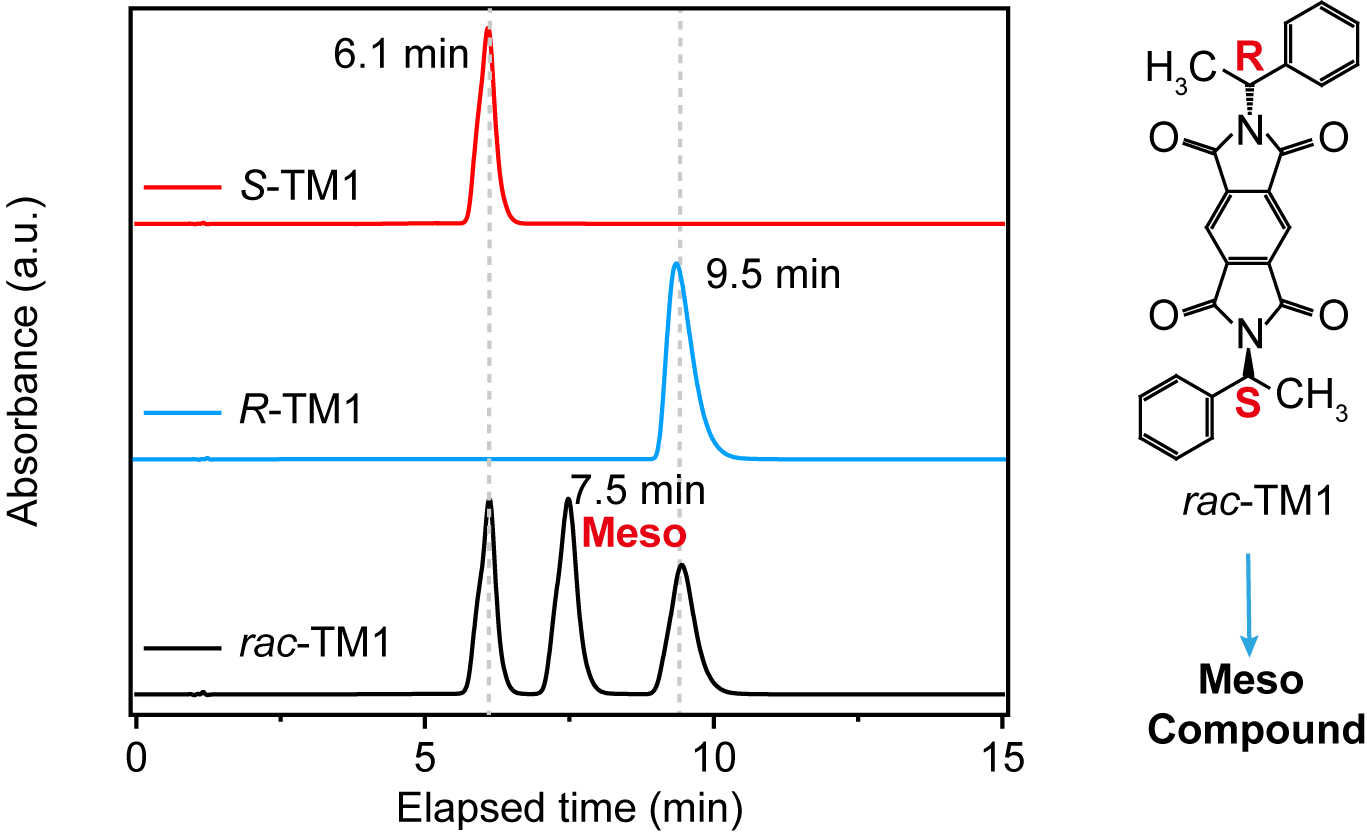


**Figure S13**. HPLC spectra of the TM1 monitored at the corresponding onset absorption bands at 280 nm with DCM-ethanol (50: 50 v/v).


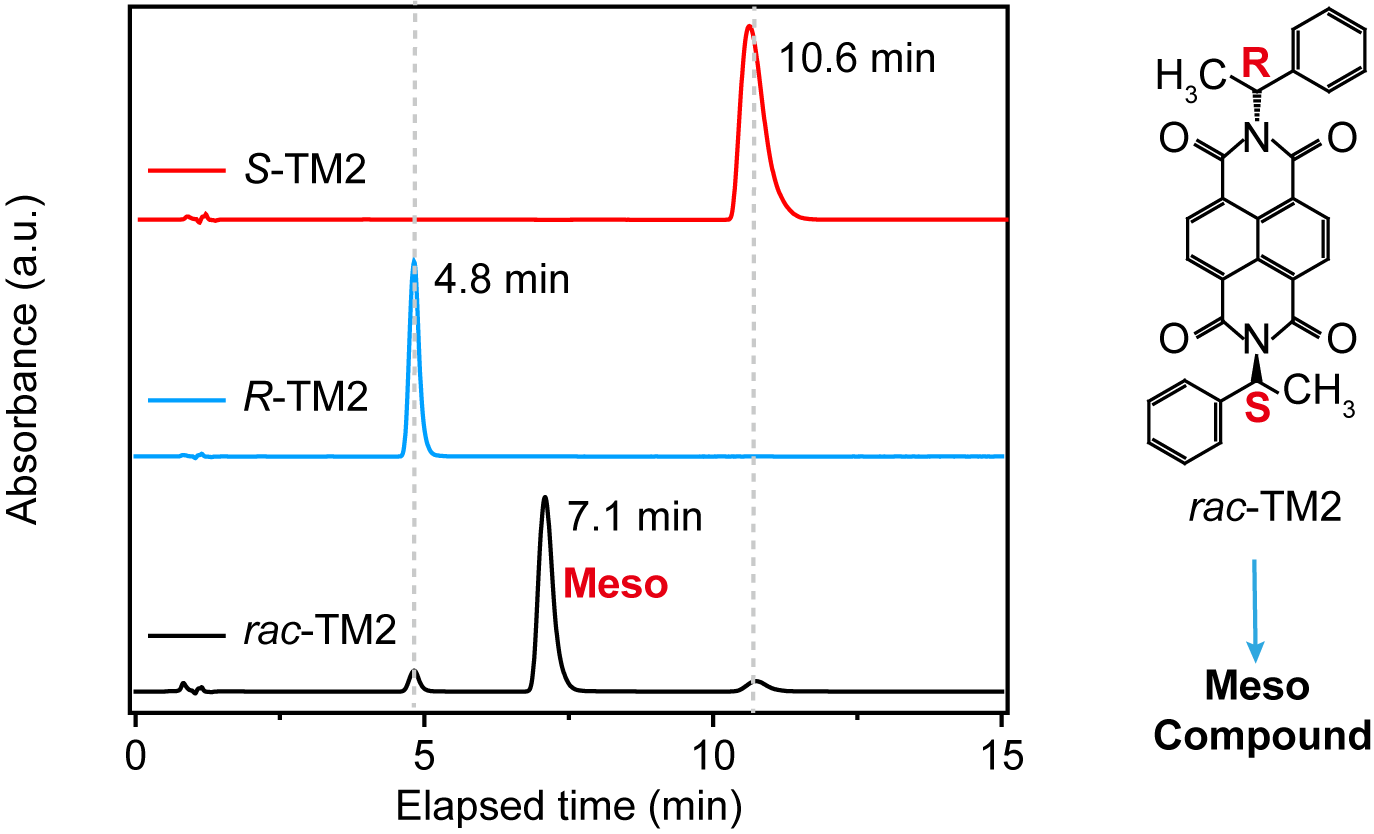


**Figure S14**. HPLC spectra of the TM2 monitored at the corresponding onset absorption bands at 350 nm with DCM-ethanol (50: 50 v/v).

# 2. Single crystal X-ray analysis

Single crystals of *R*/*S*-TM1 and *R*/*S***-**TM2 were grown by slow evaporation of combined DCM and ethanol solutions at room temperature. The crystalline data of the single crystal structures were collected on a Bruker D8 Venture using Cu-Kα radiation at 297 K. The Lorentz and polarization effect diffraction data of the samples was integrated by SAINT program and the absorption of the integrated data was corrected by SADABS program. The direct method of SHELXT-2018 was used to solve the structure, and the OLEX2 crystallography software package was used to refine the anisotropy of all non-hydrogen atoms through the full matrix least square method. Crystal structures were analyzed by Mercury 4.0 software. The crystallographic data were summarized in **Table S1**.

**Table S1.** Crystallographic data of *S*-/*R*-TM1 and *S*-/*R*-TM2 crystals at 297 K.

| Compound | *S*-TM1 | *R*-TM1 | *S*-TM2 | *R*-TM2 |
| --- | --- | --- | --- | --- |
| Formula | C_26_H_20_N_2_O_4_ | C_26_H_20_N_2_O_4_ | C_30_H_22_N_2_O_4_ | C_30_H_22_N_2_O_4_ |
| Formula weight (g mol^-1^) | 424.14 | 424.14 | 474.16 | 474.16 |
| Crystal color | colorless | colorless | pink | pink |
| Wavelength (Å) | 1.54178 | 1.54178 | 1.54178 | 1.54178 |
| Crystal system | monoclinic | monoclinic | monoclinic | monoclinic |
| Space group | *P-21* | *P-21* | *P-21* | *P-21* |
| a, (Å) | 5.6548(2) | 5.6586(2) | 9.0219(2) | 9.0223(2) |
| b, (Å) | 16.1302(6) | 16.1330(6) | 12.2345(3) | 12.2381(3) |
| c, (Å) | 11.4659(5) | 11.4607(4) | 10.8804(3) | 10.8803(2) |
| *α*, (deg) | 90 | 90 | 90 | 90 |
| *β*, (deg) | 99.7820(10) | 99.8030(10) | 108.8260(10) | 108.8330(10) |
| *γ*, (deg) | 90 | 90 | 90 | 90 |
| Volume, (Å^3^) | 1030.63(7) | 1030.97(6) | 1136.71(5) | 1137.04(4) |
| *Z* | 2 | 2 | 2 | 2 |
| Density, (g cm^-3^) | 1.368 | 1.367 | 1.386 | 1.386 |
| *μ*, (mm^-1^) | 0.759 | 0.759 | 0.752 | 0.752 |
| F (000) | 444 | 444 | 496 | 496 |
| *h*_max_, *k*_max_, *l*_max_ | 6,19,13 | 6,19,13 | 10,14,13 | 10,14,13 |
| *Theta*_max_ | 68.312 | 68.116 | 68.052 | 68.211 |
| CCDC | 2223674 | 2223673 | 2282076 | 2282087 |


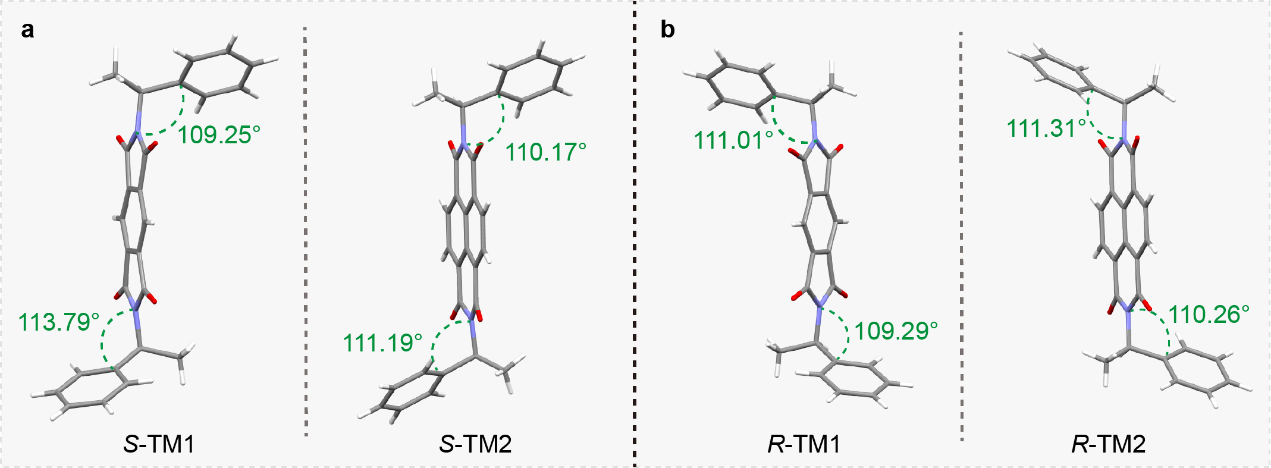


**Figure S15.** The dihedral angles between the phenyl and imide planes of the TM1 and TM2 with the **a.** *S* or **b.** *R* configuration.

# 3. Photophysical investigations

Ultraviolet-visible (UV-Vis) absorption spectra were recorded on a Jasco V-750 spectrophotometer. Steady-state photoluminescence (SSPL) spectra were recorded on an Edinburgh FLS 980 fluorescence spectrophotometer. The delayed PL spectra in dilute tetrahydrofuran were obtained using an Edinburgh FLS 980 fluorescence spectrophotometer at 77 K in a dewar vessel with a 10 ms delay time using a microsecond (μs) flash lamp. The microsecond flash lamp produces short, typically a few μs, and high irradiance optical pulses for decay measurements in the range from microseconds to seconds. The delayed PL spectra and ultralong lifetimes were also measured using an Edinburgh FLS 980 fluorescence spectrophotometer. For fluorescence lifetime measurements, a picosecond pulsed light-emitting diode (EPLED-295, wavelength: 300 nm; pulse width: 833.7 ps) was used. The multiexponential luminescence lifetimes (τ) were obtained by fitting the decay curve using the following equation

$I(t)=\sum_{i} B_{i}e^{-\frac{t}{\tau_{i}}}$ (1)

where *B*_i_ and *τ*_i_ represent the amplitudes and lifetimes of the individual components for multi-exponential decay profiles, respectively.

The average lifetime was calculated by the function of

(2)

where *φ*_i_ is the amplitude fraction.

To get the intensity-averaged lifetime (*τ*_int_), the *φ_i_*^int^ is defined by the function of

(3)

*τ*_int_ is achieved by the function of

(4)

To get the amplitude averaged lifetime (*τ*_amp_) which was used for the analyses of SACET process, the *φ*_i_^amp^ is defined by the function of:

(5)

*τ*_amp_ is achieved by the function of:

(6)

The absolute PLQYs were measured using an integrating sphere, and the wavelength-dependent sensitivity of the detector has been calibrated automatically by Edinburgh Instruments during PLQY measurement. The absolute PLQYs is, by definition, the ratio of the number of photons emitted to the number of photons absorbed, as descripted in the following equation:

$\Phi=\frac{N_{E\text{m}}}{N_{A\text{bs}}}=\frac{E_{B}-E_{A}}{S_{A}-S_{B}}$ (7)

Where *N*_Em_ and *N*_Abs_ are the number of emitted photons and absorbed photons respectively; *S*_A_ and *S*_B_ refer to the integral areas of the scans of excitation scatter regions of the supplied blanking plug made from poly tetra fluoroethylene (PTFE) and the measured sample, respectively. *E*_B_ and *E*_A_ represent the integral areas of emission regions of the measured sample and the supplied blanking plug, respectively.

The energy transfer efficiency was calculated by the following equation:

$\Phi_{ET}=1-\tau_{\text{amp}}^{\text{H, P}}/\tau_{\text{amp}}^{\text{P}}$ (8)

H: host; P: phosphorescence; where *τ*H, P ampand *τ*P amp are the phosphorescence amplitude averaged lifetime of TM1 with and without of TM2.

Excitation-PL mapping was measured using Hitachi F-4700 under ambient condition. The intrinsic circularly polarized luminescence (CPL) spectra were investigated using a JASCO CPL-300 spectrometer. The scan speed was set as 500 nm/min with 0.1 nm resolution and a respond time of 1.0 s. The circular dichroism (CD) spectra were measured on a JASCO J-810 circular dichroism spectrometer with ‘Low’ sensitivity. The scan speed was set as 200 nm/min with 1 nm resolution and a respond time of 1.0 s. Transient absorption measurement were performed by Edinburgh LP 900 transient absorption spectrophotometer equipped with a SLI-10 laser.


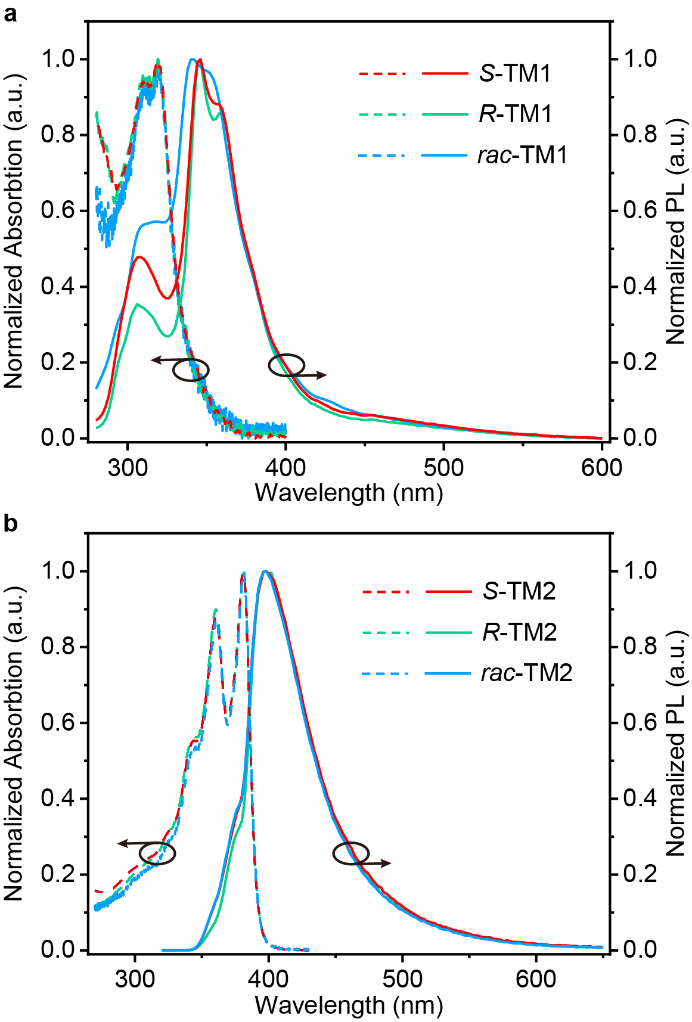


**Figure S16.** Absorption and steady state photoluminescence (SSPL) spectra of (**a**) TM1 and (**b**) TM2 in tetrahydrofuran (THF, ~10^-5^ mol L^-1^) under ambient conditions.


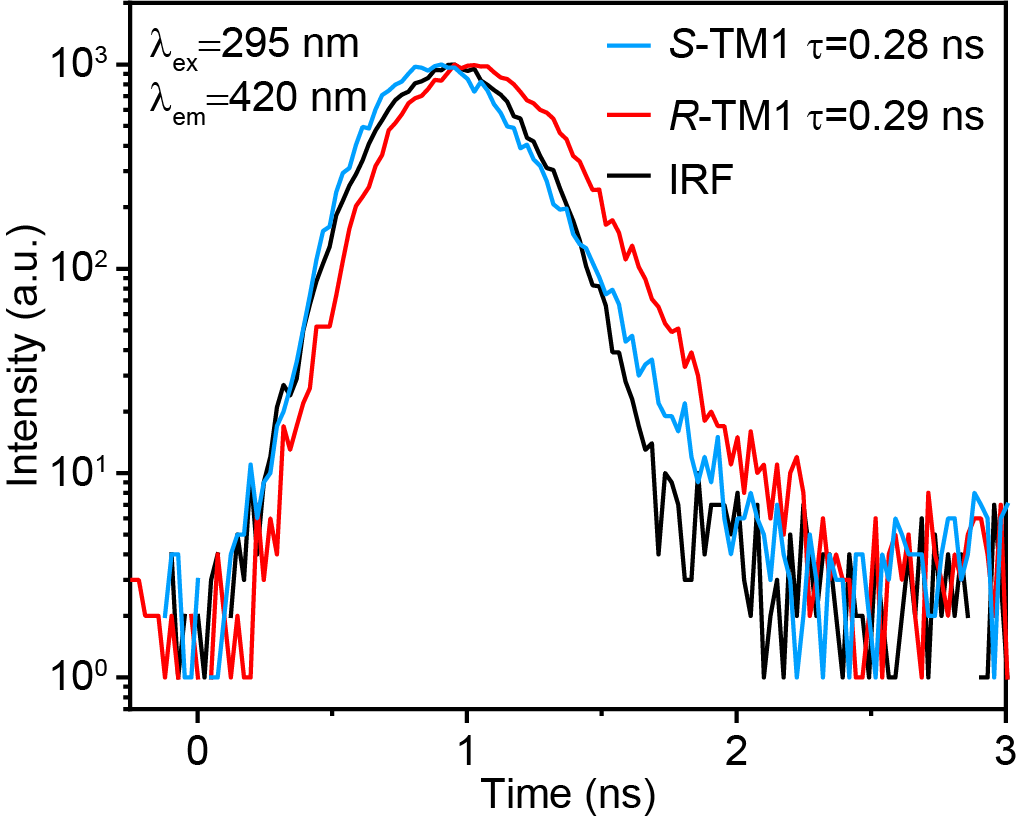


**Figure S17.** Fluorescence lifetime decay profiles of *S*-TM1 and *R*-TM2 crystals excited by 295 nm UV light under ambient conditions. IRF=instrument response function (black line).


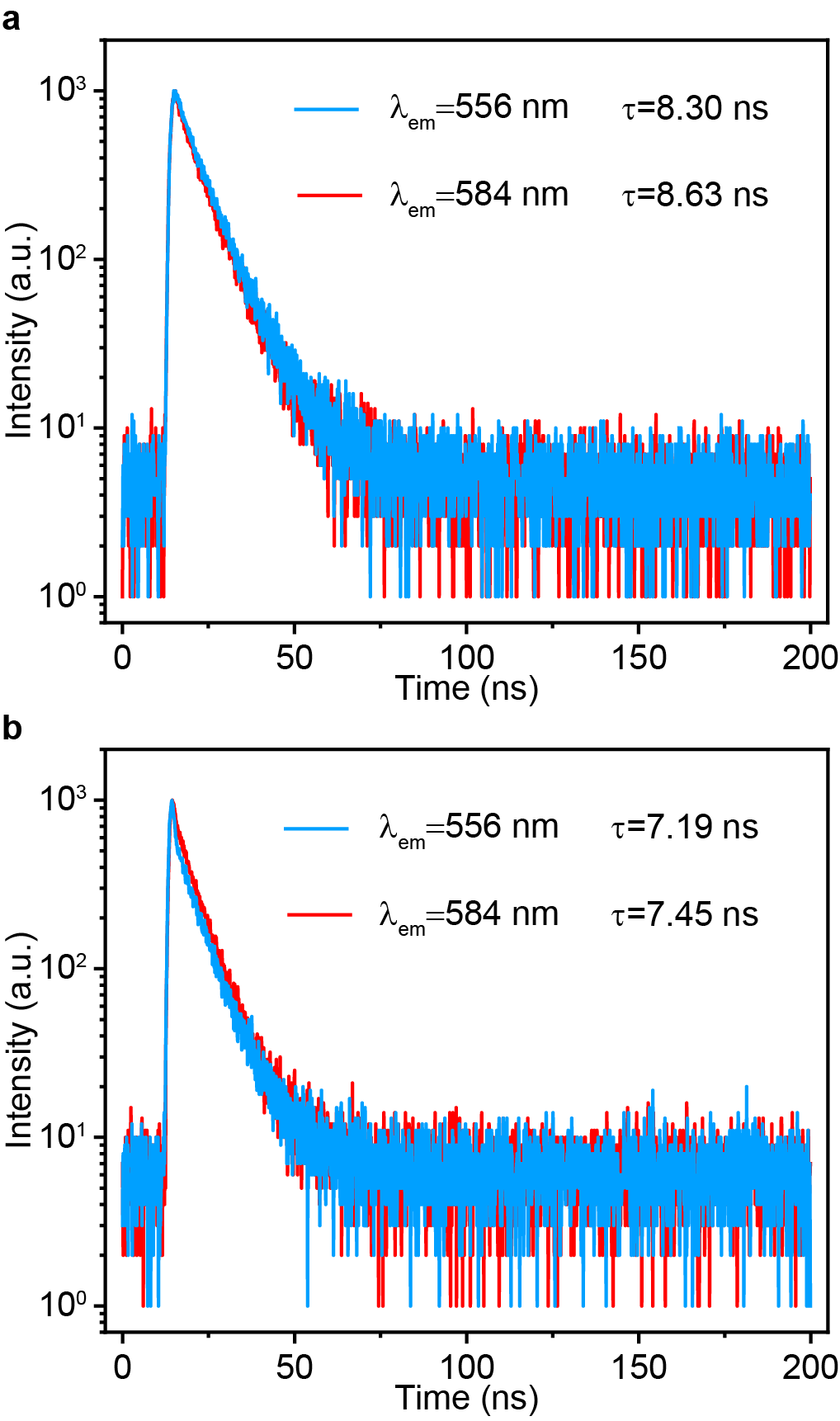


**Figure S18.** Fluorescence lifetime decay profiles of (**a**) *S*-TM2 and (**b**) *R*-TM2 crystals excited by 295 nm UV light under ambient conditions.


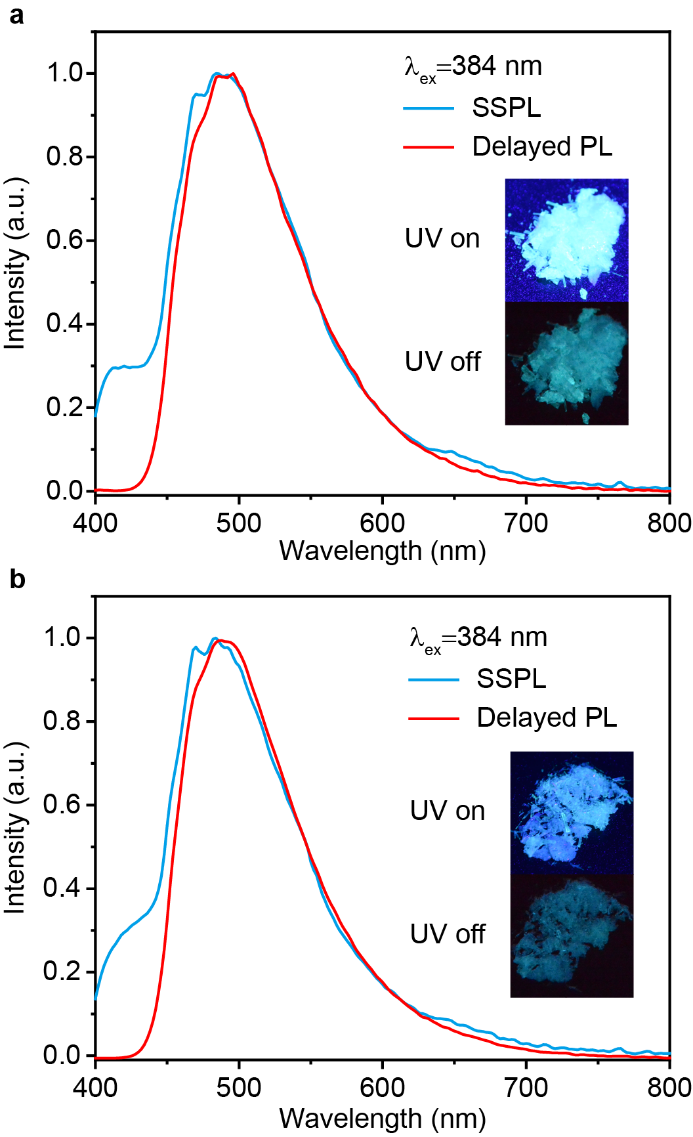


**Figure S19.** SSPL and delayed PL spectra of (**a**) *S*-TM1 and (**b**) *R*-TM1 crystals under ambient conditions.


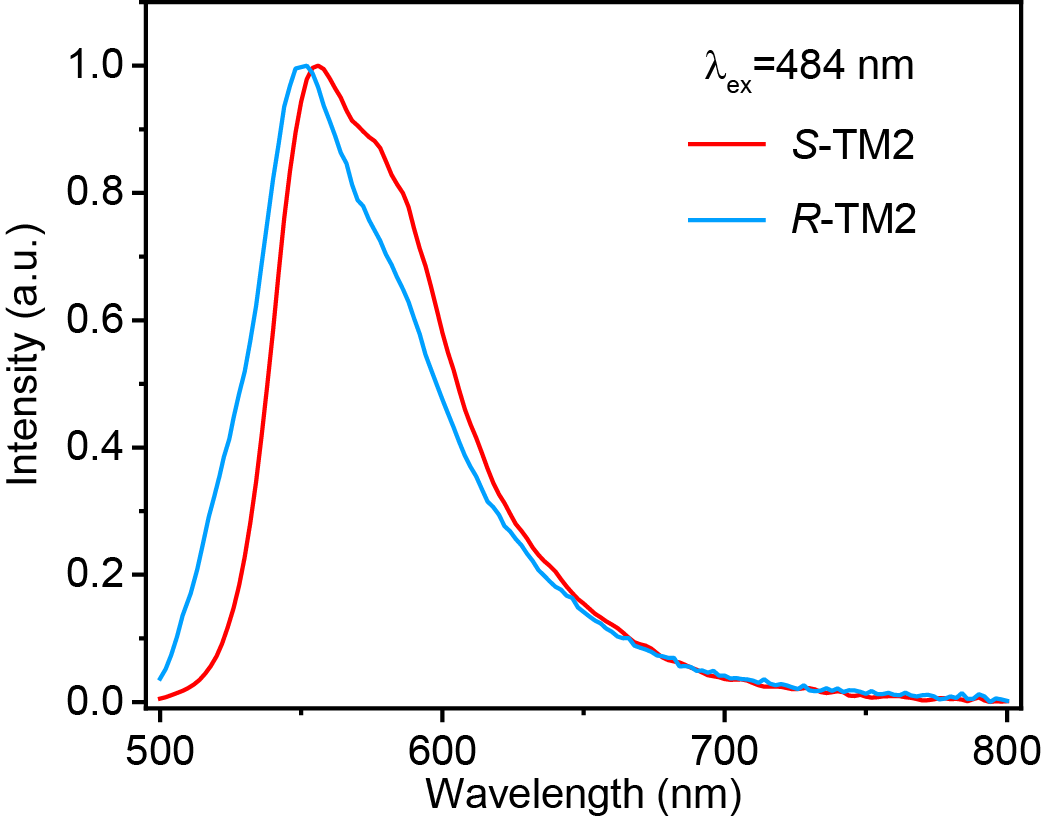


**Figure S20**. SSPL spectra of *S*-TM2 and *R*-TM2 crystals excited by 484 nm blue light under ambient conditions.


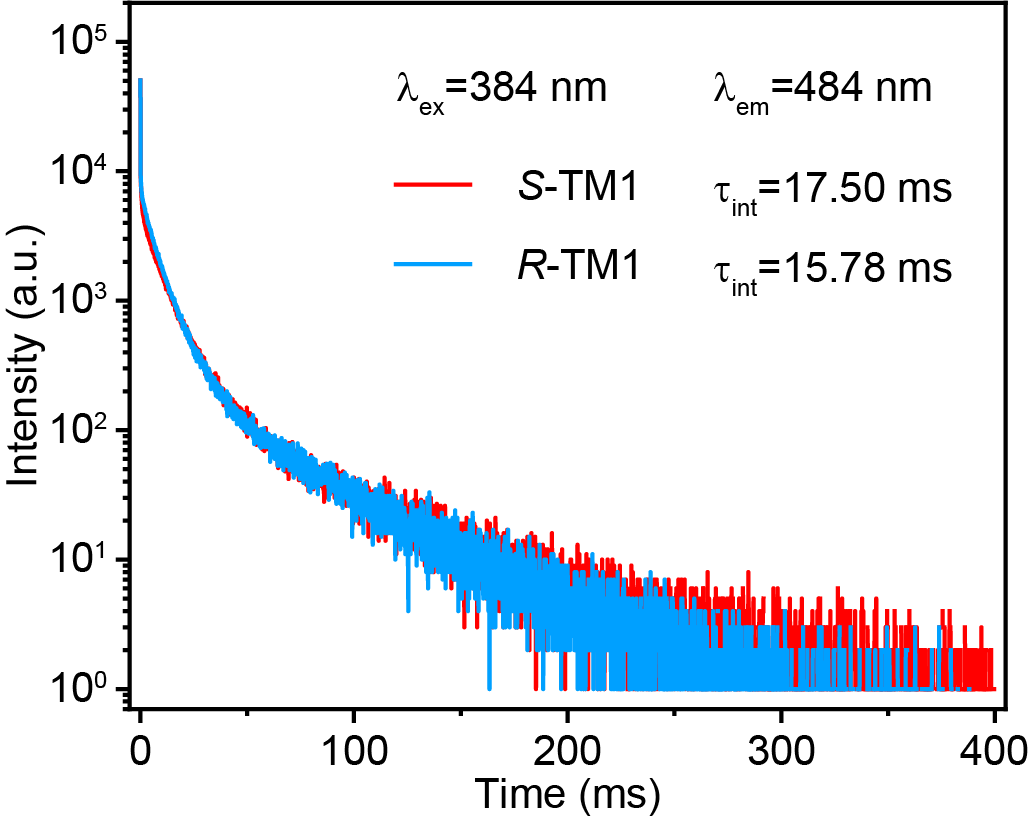


**Figure S21.** Phosphorescence lifetime decay profiles of emission at 484 nm of *S*-TM1 and *R*-TM1 crystals excited by 384 nm UV light under ambient conditions.


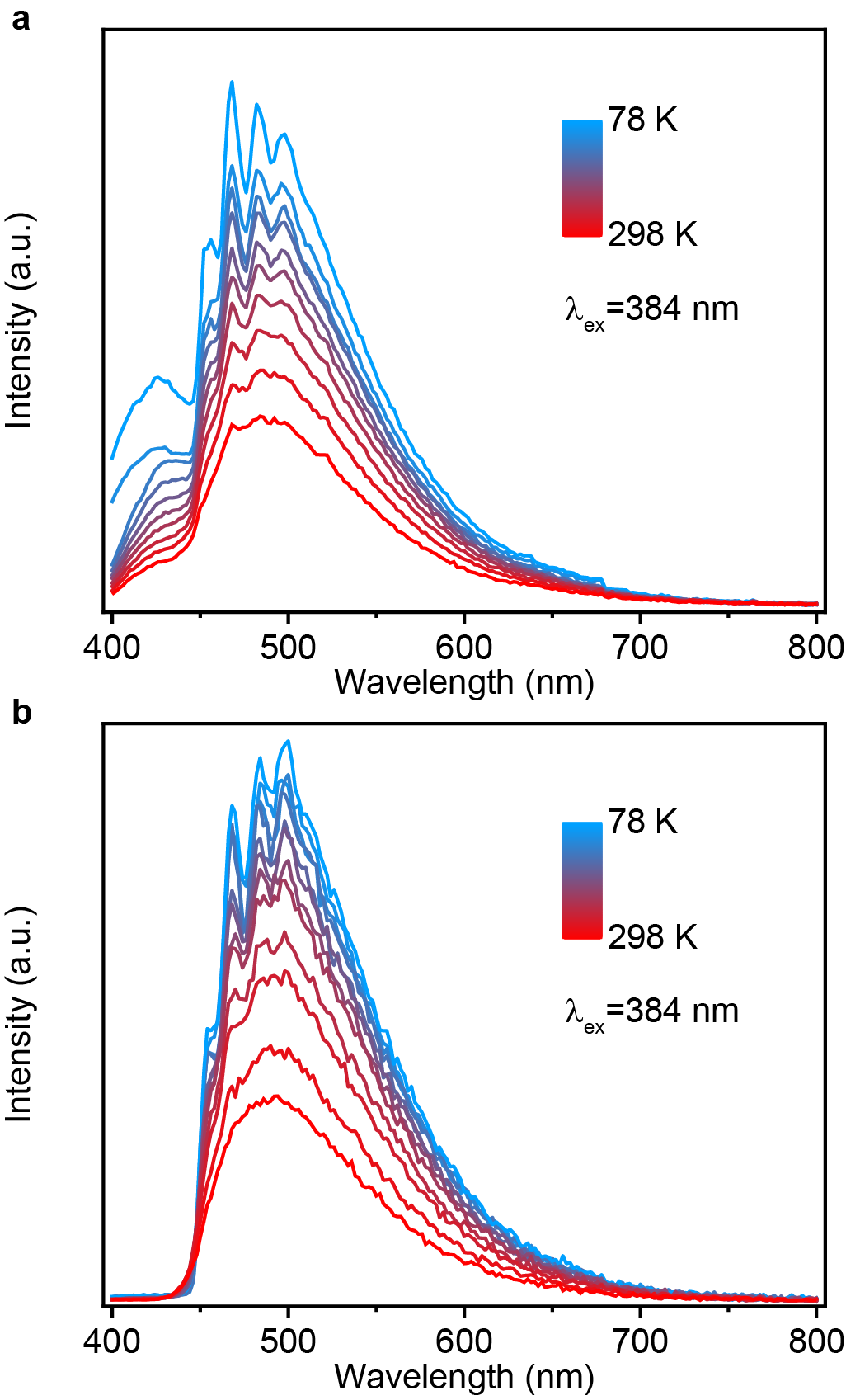


**Figure S22.** Temperature dependent (**a**) SSPL and (**b**) delayed PL spectra of *S*-TM1 crystal excited by 384 nm UV light.


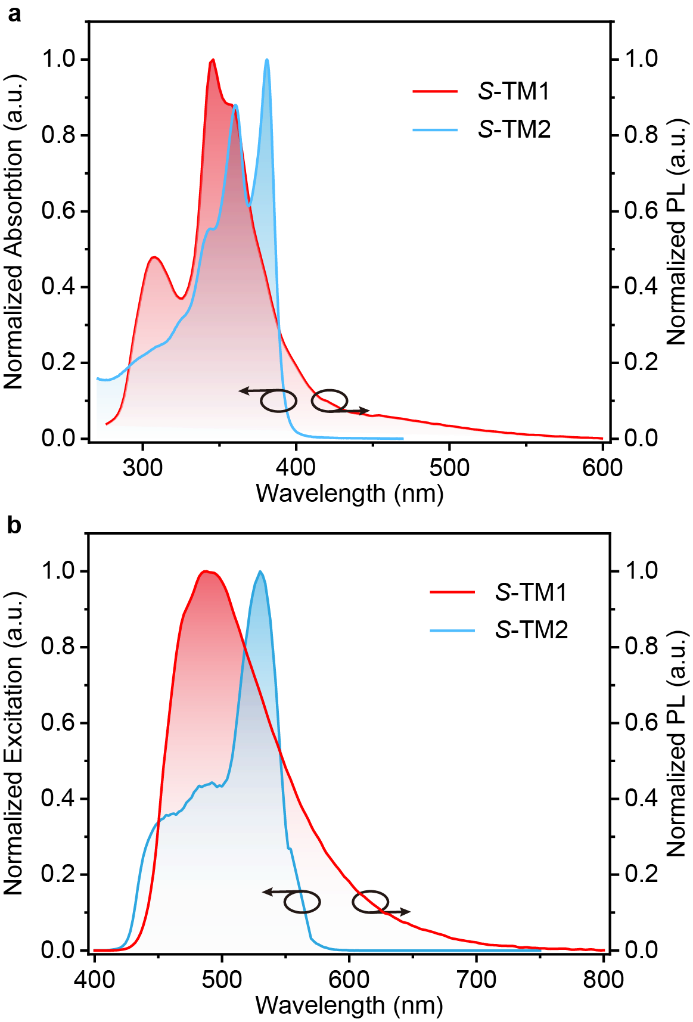


**Figure S23.** SSPL spectrum of *S*-TM1 and UV-visible absorption spectrum of *S*-TM2 in THF (~10^-5^ mol L^-1^) (**a**); delayed PL spectrum of *S*-TM1 and excitation spectrum of *S*-TM2 in crystal (**b**) under ambient conditions.


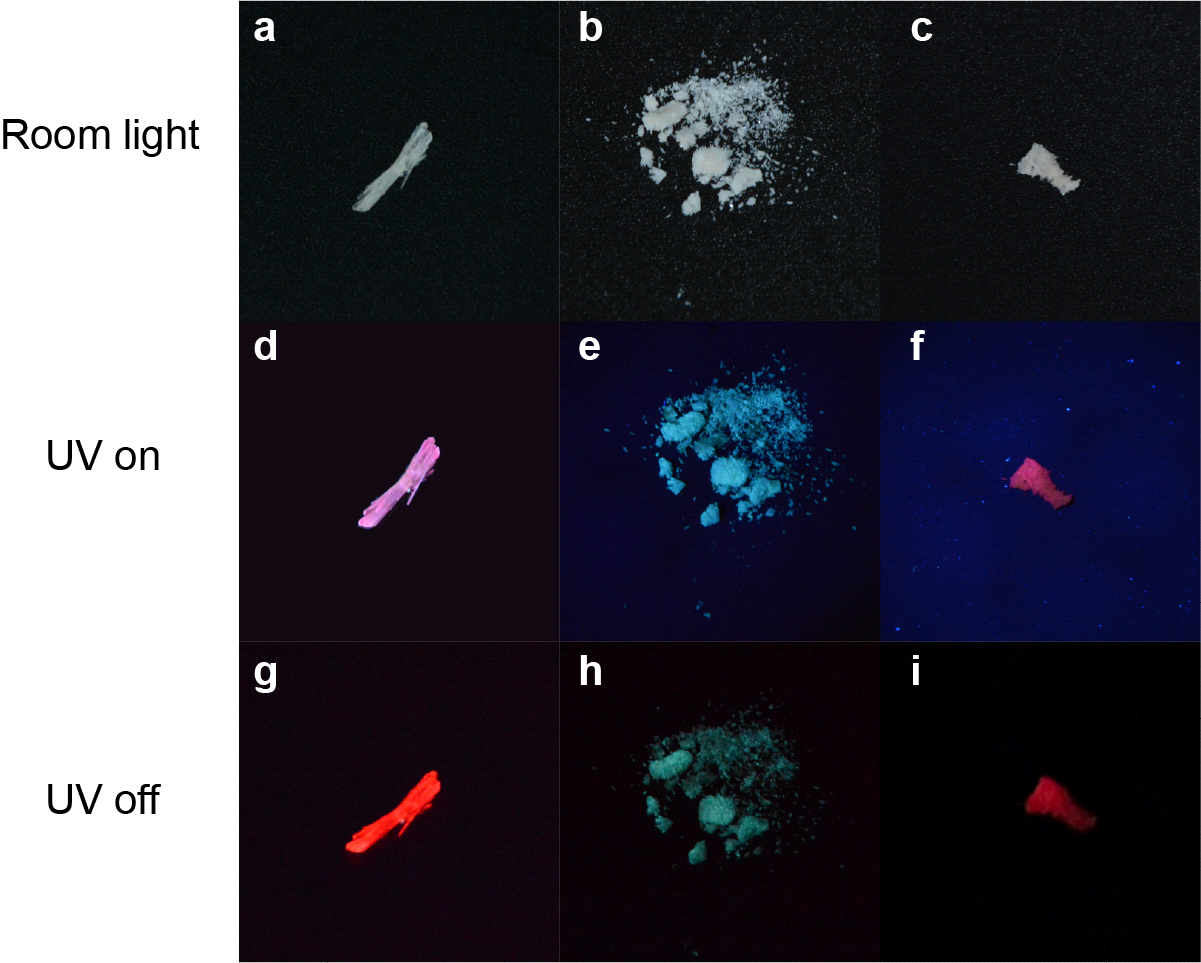


**Figure S24.** Photographs of *S*-TM1/*S*-TM2 (left), *S*-TM1/*R*-TM2 (middle) and *S*-TM1/*rac*-TM2 (right) doped crystals with a weight ratio of 25/1 under room light (**a**, **b**, **c**), under 365 nm UV light excitation (**d**, **e**, **f**) and after removal of 365 nm UV (**g**, **h**, **i**).


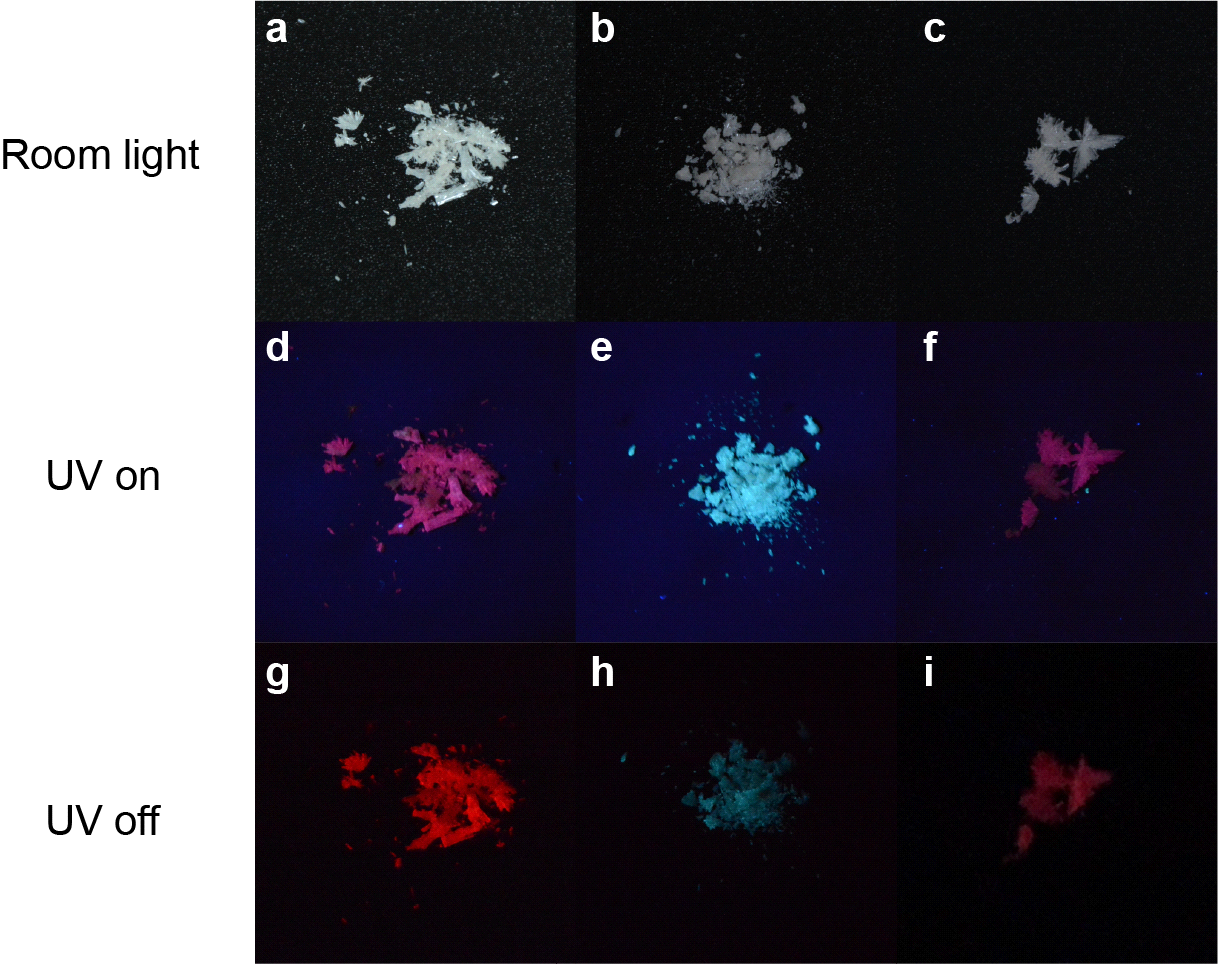


**Figure S25.** Photographs of *R*-TM1/*R*-TM2 (left), *R*-TM1/*S*-TM2 (middle) and *R*-TM1/*rac*-TM2 (right) doped crystals with a weight ratio of 25/1 under room light (**a**, **b**, **c**), under 365 nm UV light excitation (**d**, **e**, **f**) and after removal of 365 nm UV (**g**, **h**, **i**).


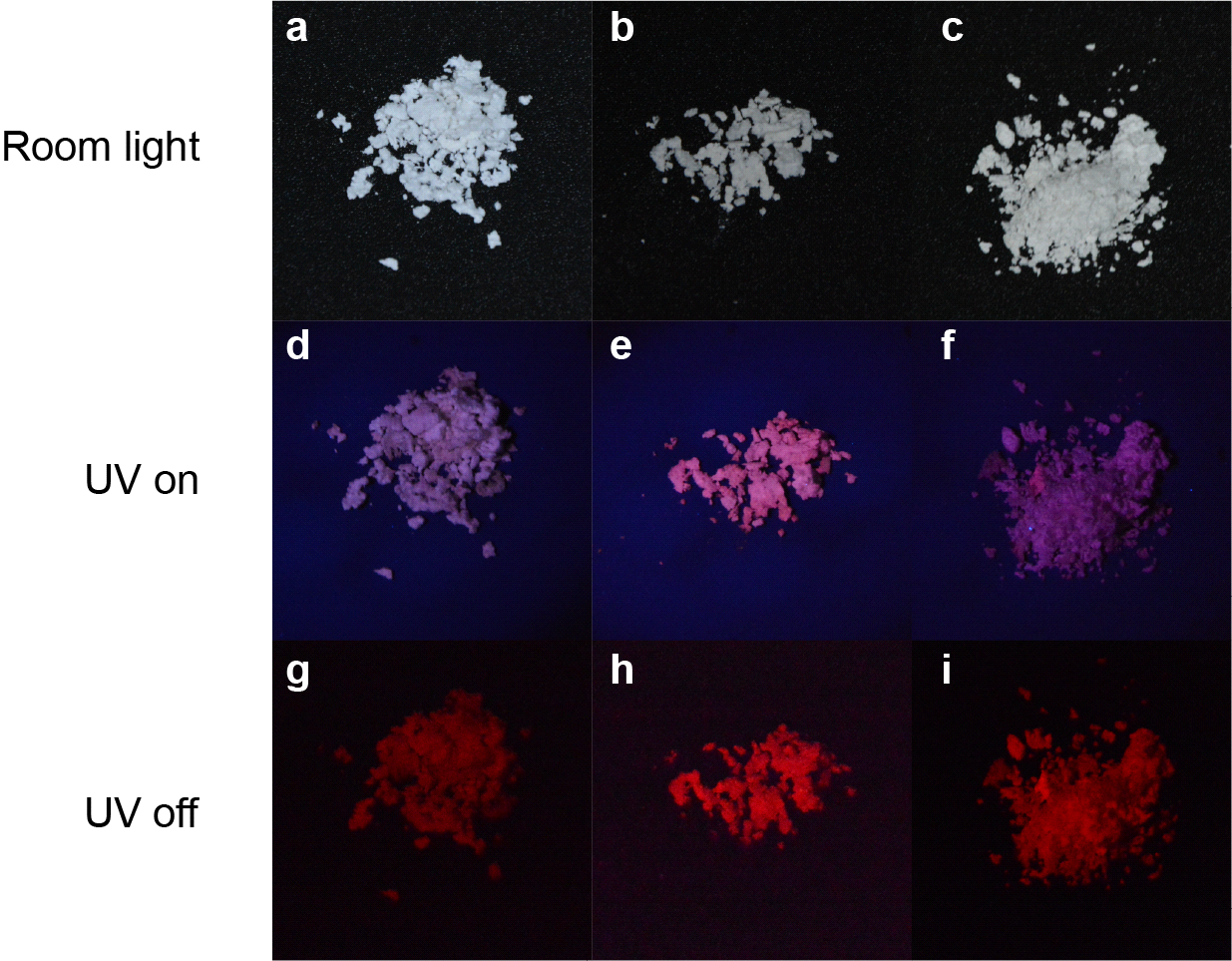


**Figure S26.** Photographs of *rac-*TM1/*S-*TM2 (left), *rac-*TM1/*R-*TM2 (middle) and *rac-*TM1/*rac-*TM2 (right) doped crystals with a weight ratio of 25/1 under room light (**a**, **b**, **c**), under 365 nm UV light excitation (**d**, **e**, **f**) and after removal of 365 nm UV (**g**, **h**, **i**).


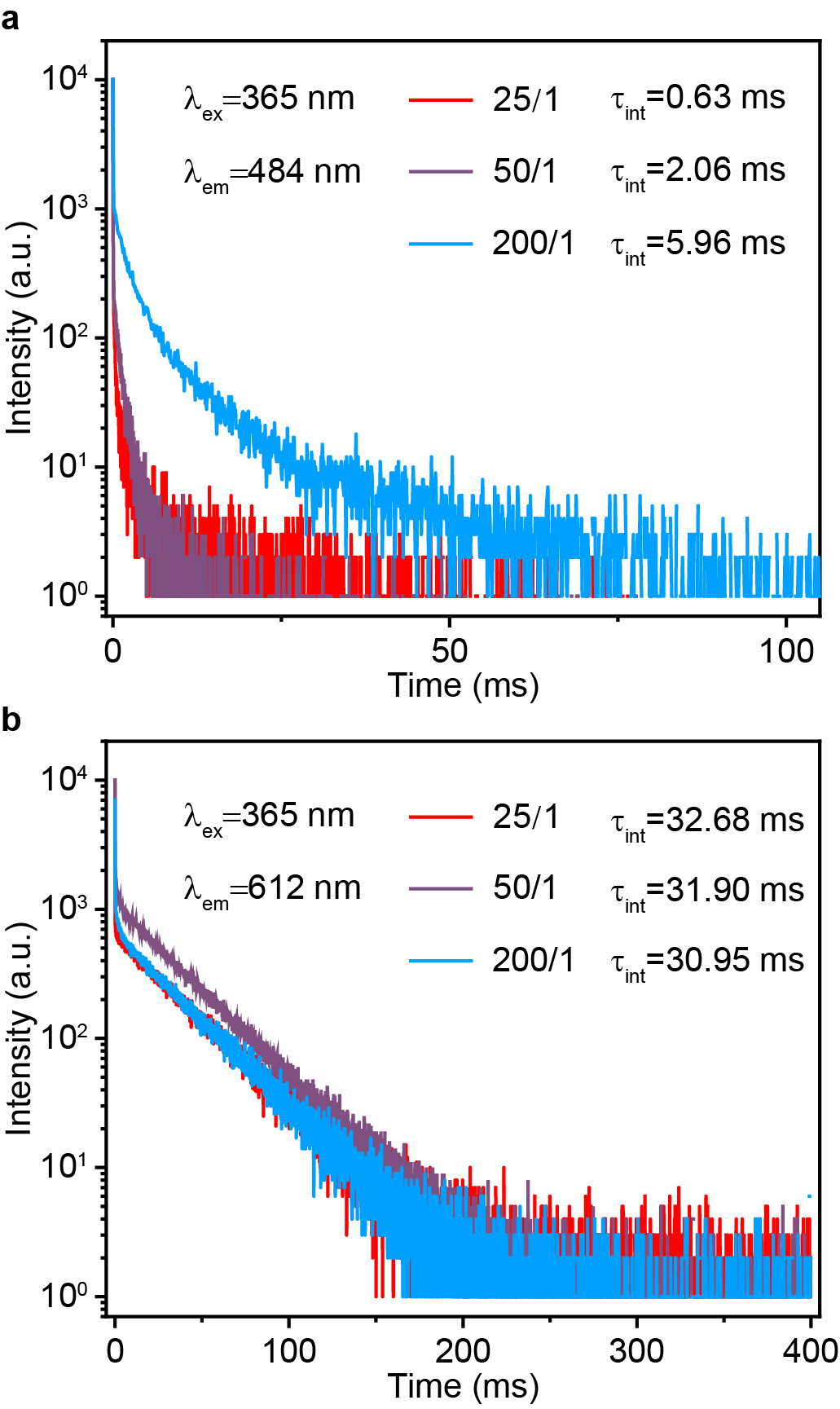


**Figure S27.** Phosphorescence lifetime decay profiles of *S*-TM1/*S*-TM2 doped crystals with different weight ratios by monitoring the emission bands at (**a**) 484 and (**b**) 612 nm under ambient conditions.


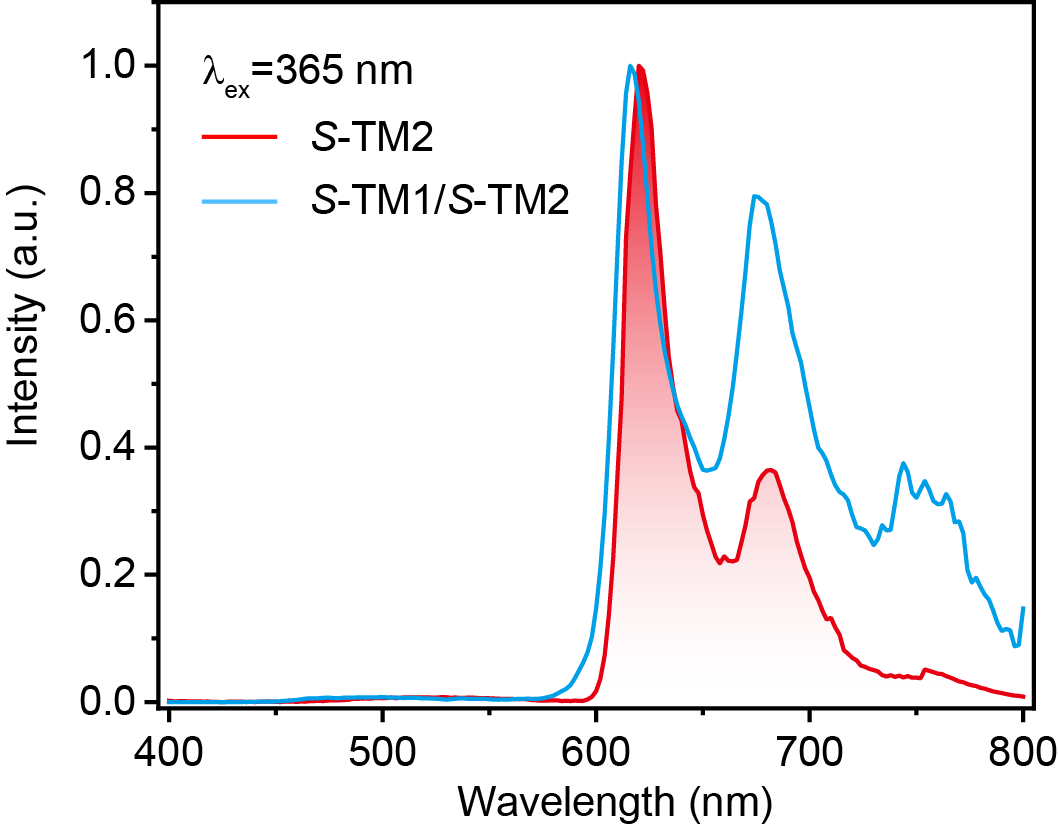


**Figure S28.** Delayed PL spectra of *S*-TM2 in 1,6-diiodohexane under cryogenic temperature (77 K) and *S*-TM1/*S*-TM2 doped crystals with a weight ratio of 25/1 under ambient conditions.


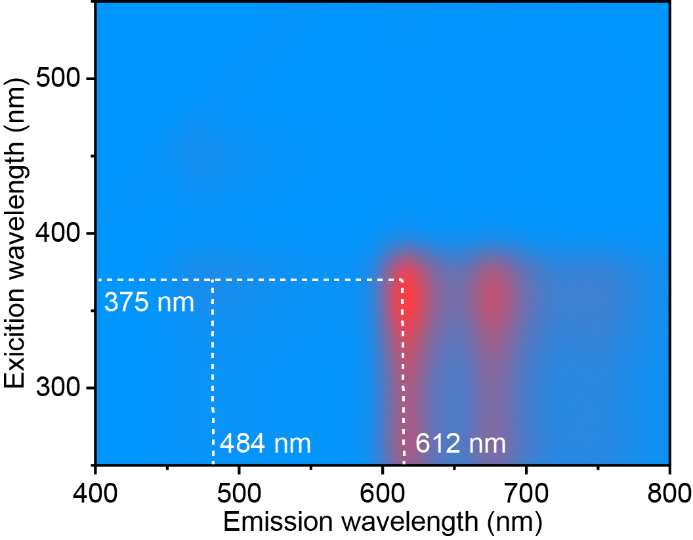


**Figure S29.** Excitation-delayed PL emission mapping of *S*-TM1/*S*-TM2 crystals with a delayed time of 25 ms under ambient conditions.


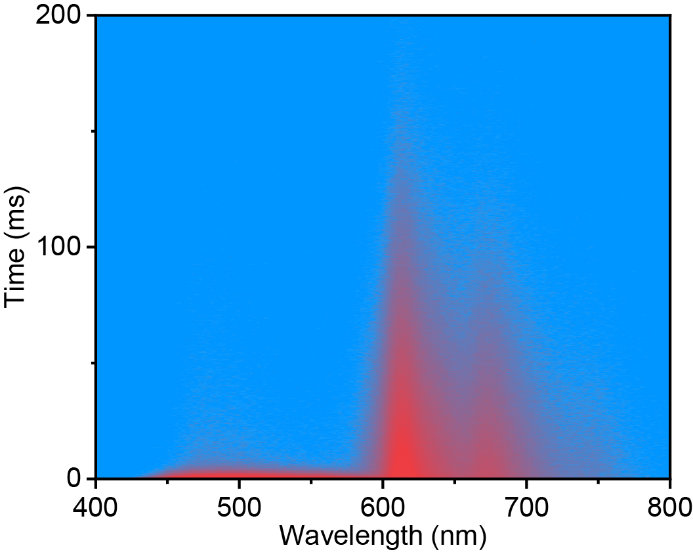


**Figure S30.** Transient emission decay images of *S*-TM1/*S*-TM2 crystals with a doping ratio of 25/1 excited by 365 nm UV light under ambient conditions.


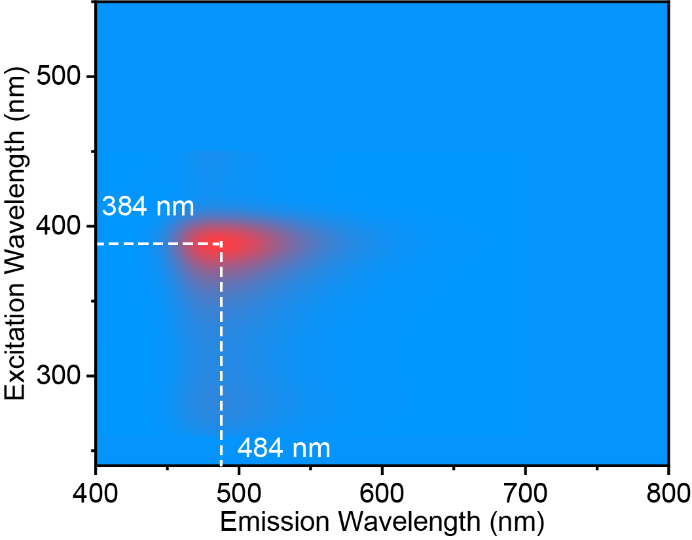


**Figure S31.** Excitation-delayed PL emission mapping of *R*-TM1 crystal with a delayed time of 25 ms under ambient conditions.


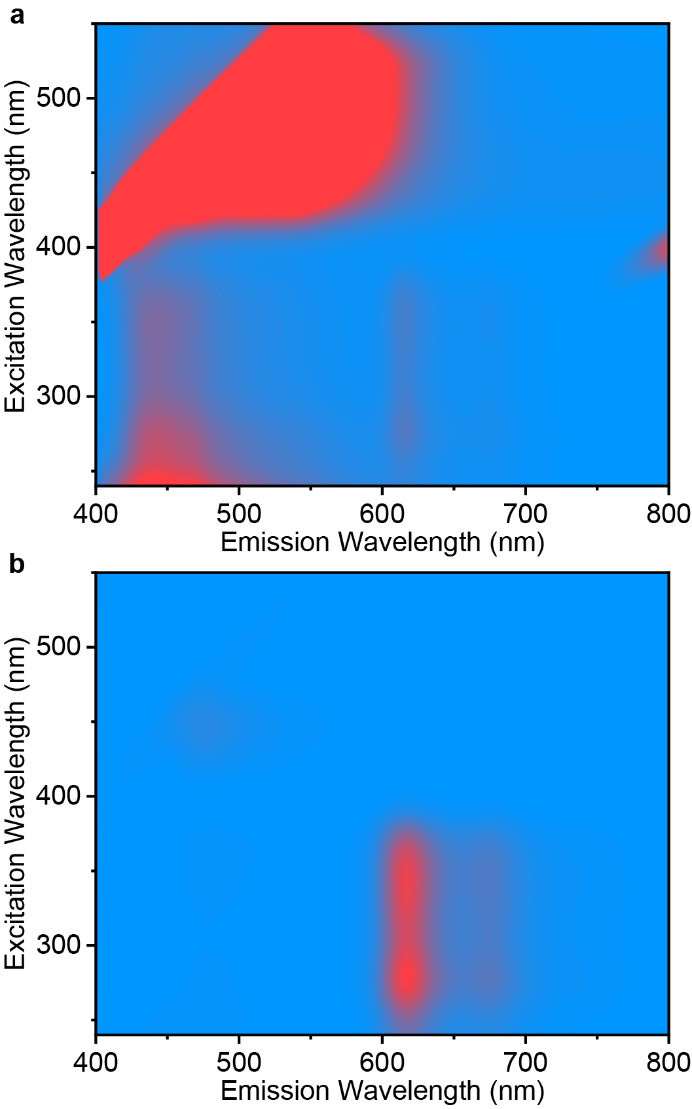


**Figure S32.** (**a**) Excitation-SSPL emission mapping and (**b**) excitation-delayed PL emission mapping of *R*-TM1/*R*-TM2 crystals with a delayed time of 25 ms under ambient conditions.


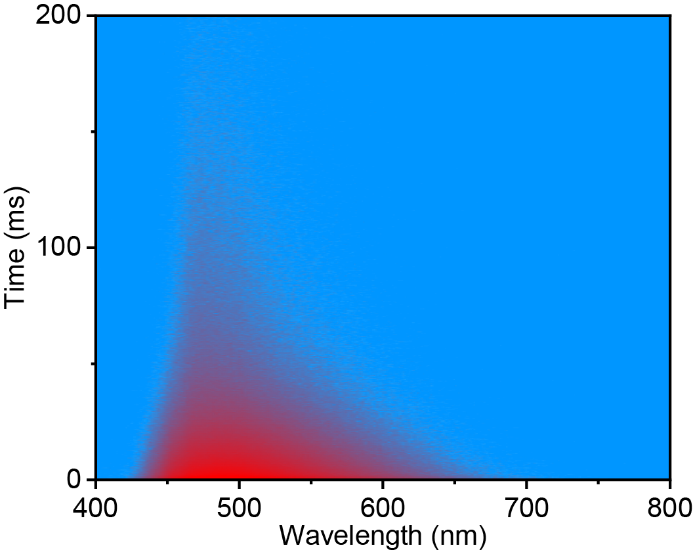


**Figure S33.** Transient emission decay images of *R*-TM1 crystal excited by 384 nm UV light under ambient conditions.


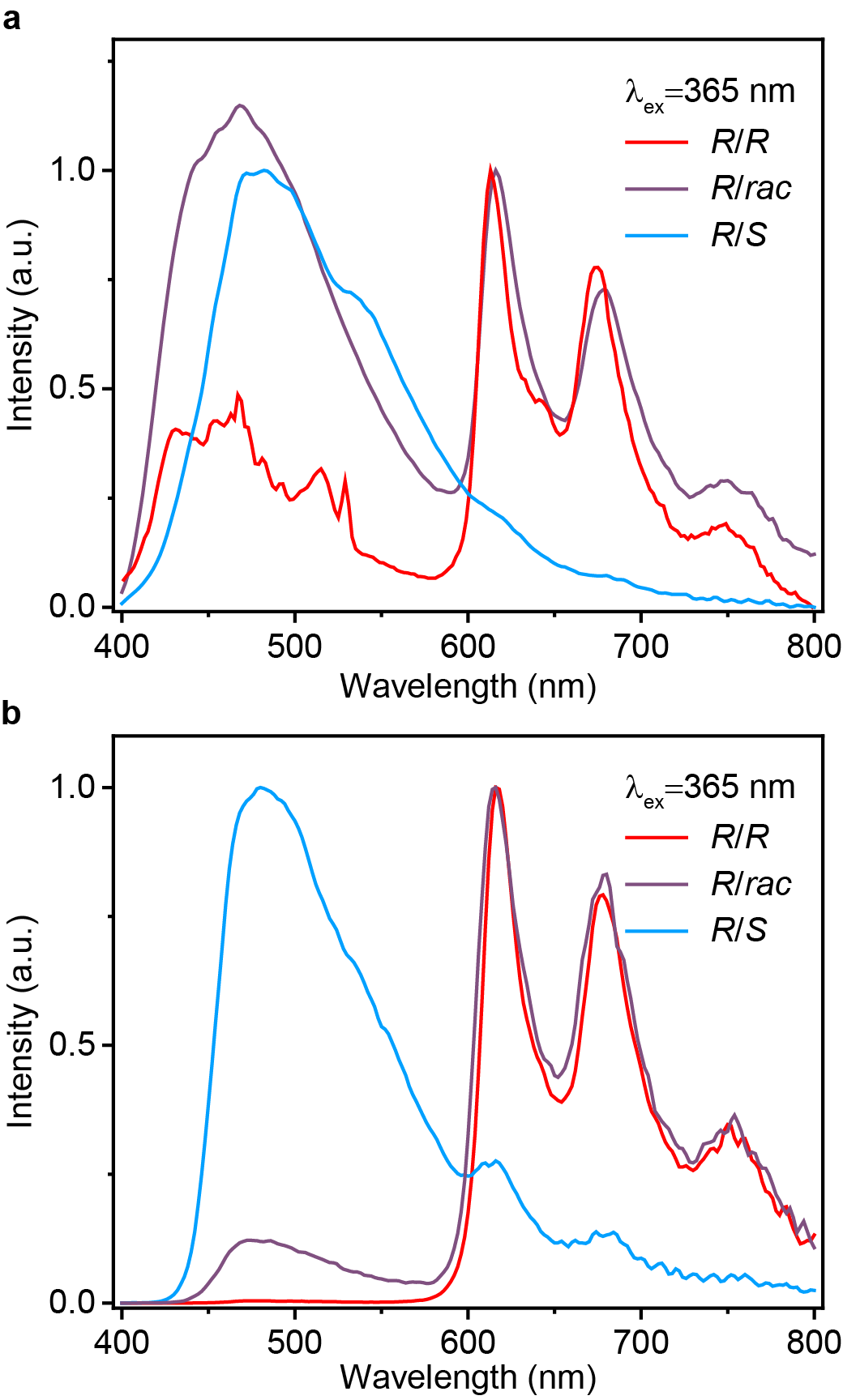


**Figure S34.** (**a**) SSPL and (**b**) delayed PL spectra of *R***-**TM1/*R***-**TM2 (*R*/*R*), *R*-TM1/*rac***-**TM2 (*R*/*rac*) and *R*-TM1/*S*-TM2 (*R*/*S*) doped crystals with a weight ratio of 25/1 under ambient conditions, respectively.


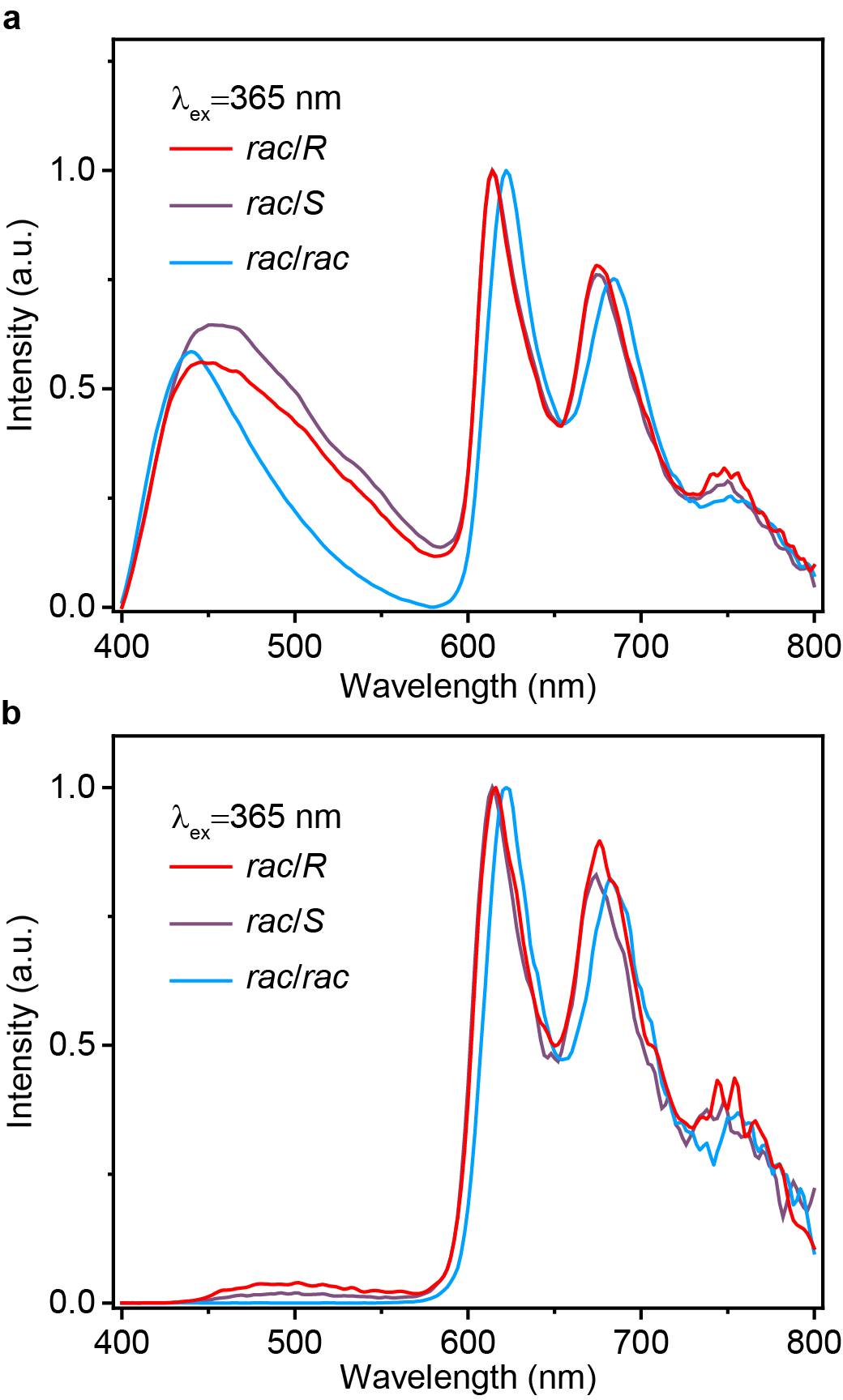


**Figure S35.** (**a**) SSPL and (**b**) delayed PL spectra of *rac*-TM1**/***S*-TM2 (*rac*/*S*), *rac*-TM1**/***R***-**TM2 (*rac*/*R*) and *rac*-TM1**/***rac*-TM2 (*rac*/*rac*) doped crystals with a weight ratio of 25/1 under ambient conditions, respectively.


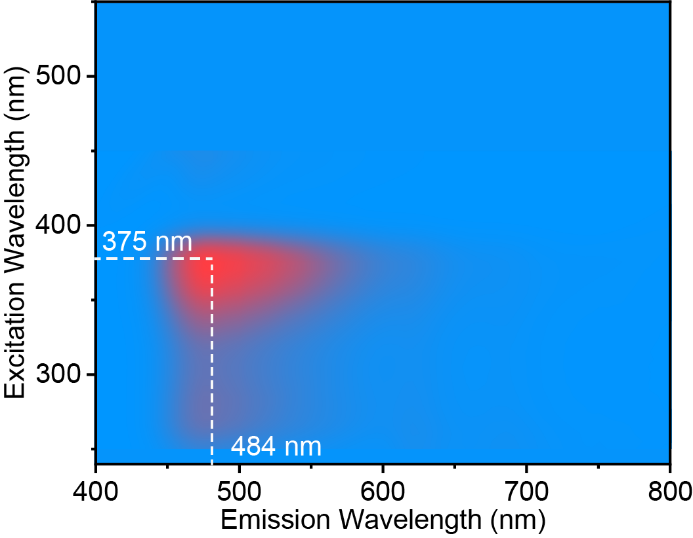


**Figure S36.** Excitation-delayed PL emission mapping of *S*-TM1/*R*-TM2 doped crystals with a weight ratio of 25/1 with a delayed time of 25 ms under ambient conditions.


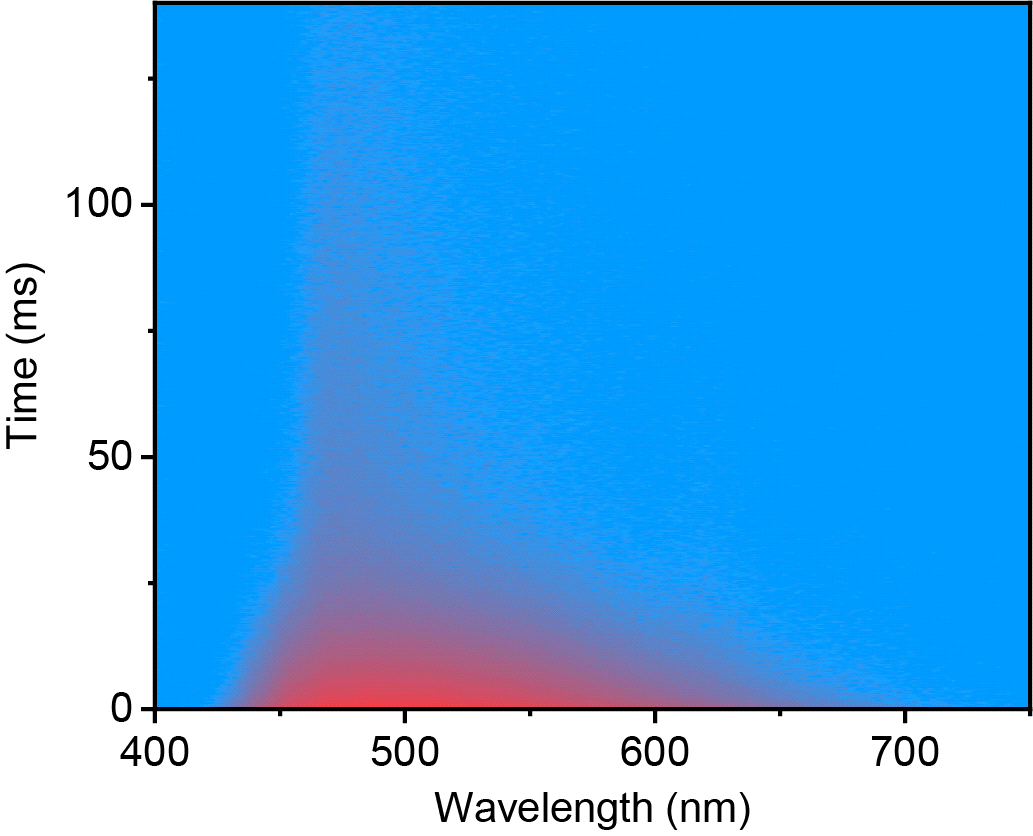


**Figure S37.** Transient emission decay images of *S*-TM1/*R*-TM2 doped crystals with a weight ratio of 25/1 excited by 365 nm UV light under ambient conditions.


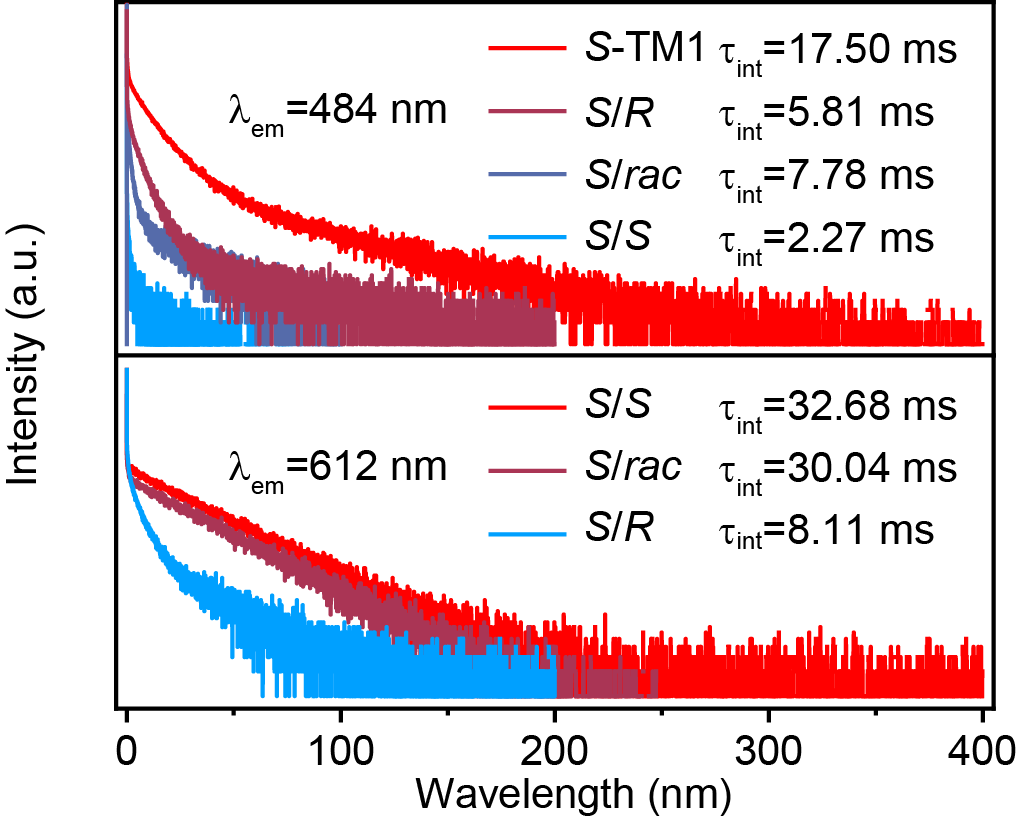


**Figure S38.** Phosphorescence lifetime decay profiles of *S*-TM1/*S***-**TM2 (*S*/*S*), *S*-TM1/*rac*-TM2 (*S*/*rac*) and *S*-TM1/*R*-TM2 (*S*/*R*) doped crystals with weight ratios of 25/1 by monitoring the emission bands at 484 and 612 nm under ambient conditions.


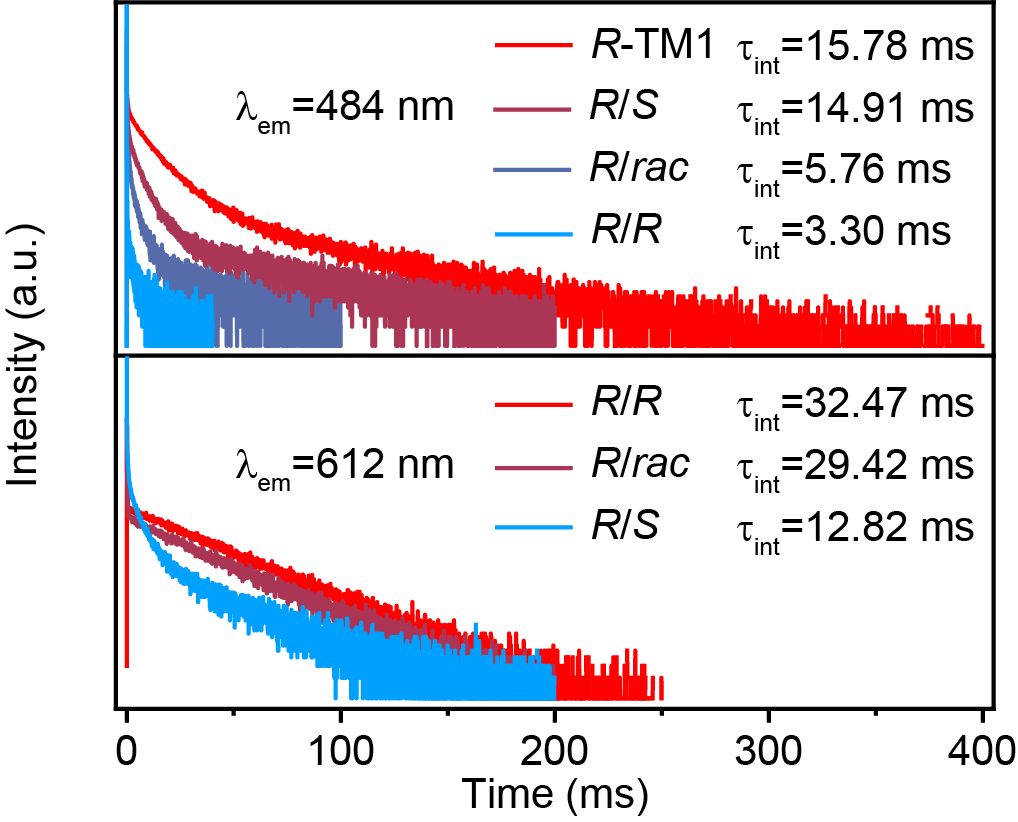


**Figure S39.** Phosphorescence lifetime decay profiles of *R*-TM1/*R*-TM2 (*R*/*R*), *R*-TM1/*rac*-TM2 (*R*/*rac*) and *R*-TM1/*S*-TM2 (*R*/*S*) doped crystals with weight ratios of 25/1 by monitoring the emission bands at 484 and 612 nm under ambient conditions.


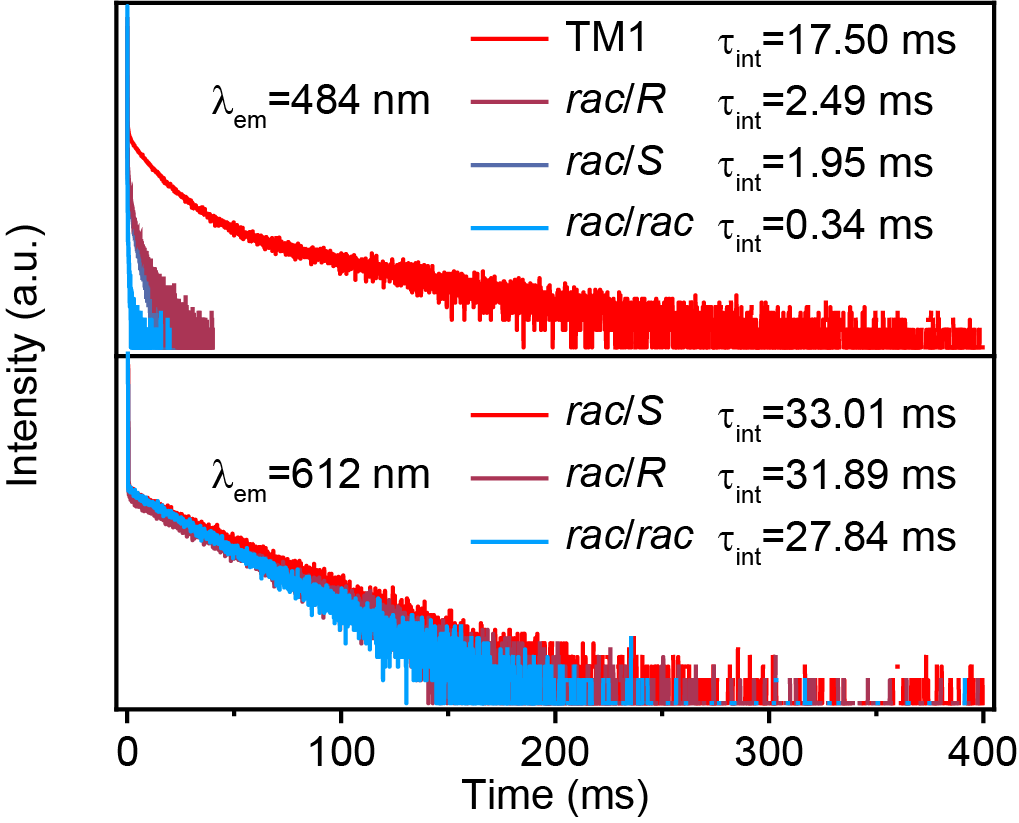


**Figure S40.** Phosphorescence lifetime decay profiles of *rac*-TM1/*R*-TM2 (*rac*/*R*), *rac*-TM1/*S*-TM2 (*rac*/*S*) and *rac*-TM1/*rac*-TM2 (*rac*/*rac*) doped crystals with weight ratios of 25/1 by monitoring the emission bands at 484 and 612 nm under ambient conditions.


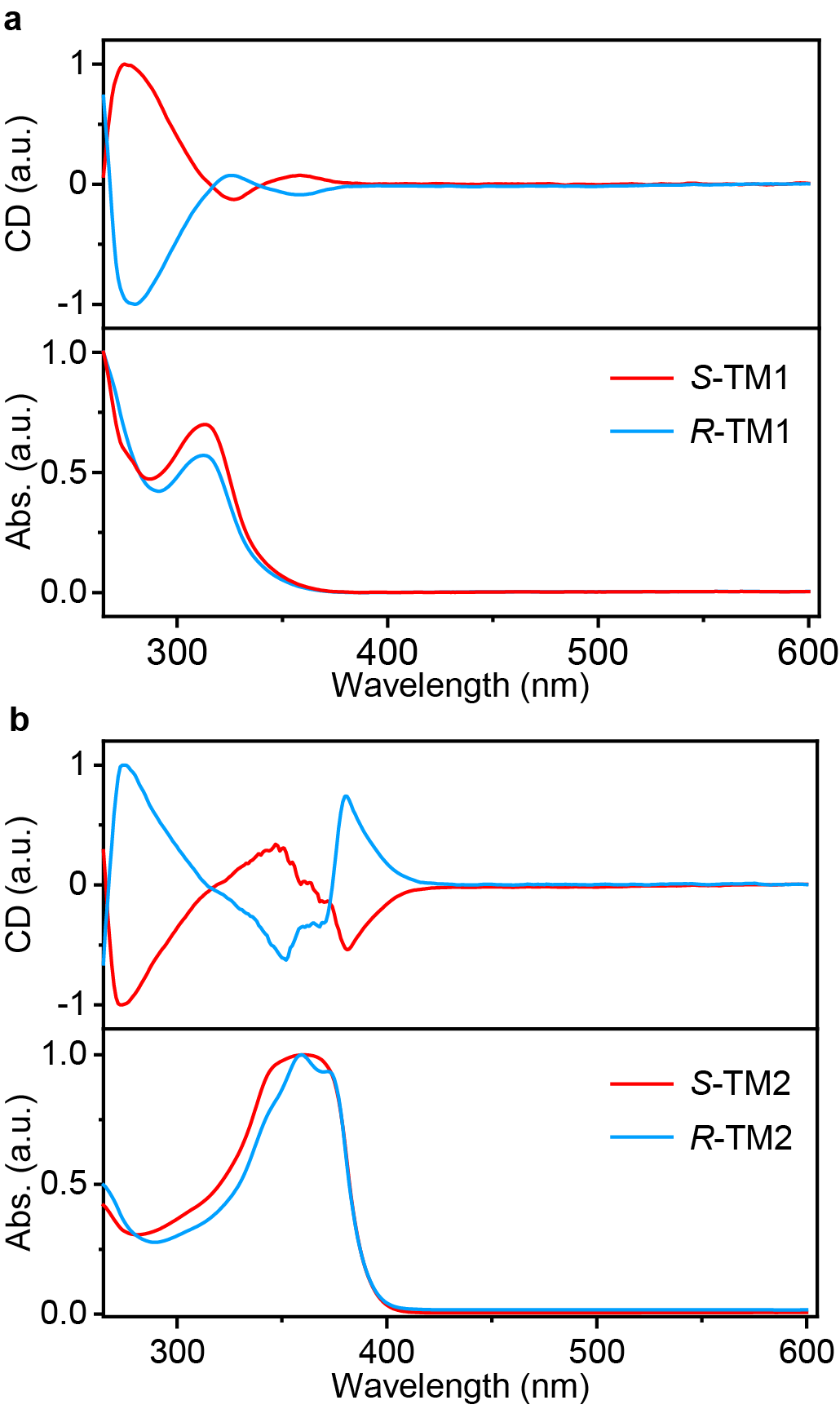


**Figure S41.** Circular dichroism (CD) spectra of (**a**) TM1 and (**b**) TM2 in THF (~10^-3^ mol L^-1^) under ambient conditions.


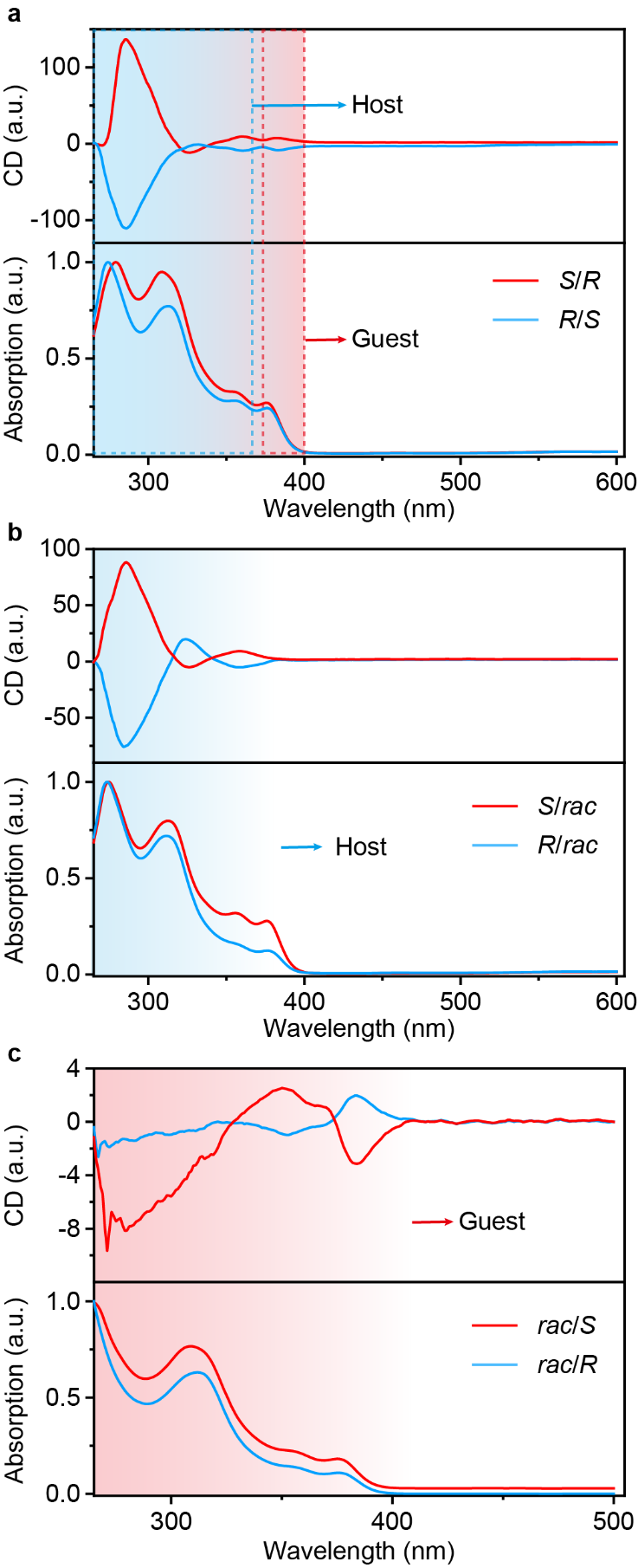


**Figure S42.** CD spectra of (**a**) *S*-TM1/*R*-TM2 (*S*/*R*) and *R*-TM1/*S*-TM2 (*R*/*S*), (**b**) *S*-TM1/*rac*-TM2 (*S*/*rac*) and *R*-TM1/*rac*-TM2 (*R*/*rac*), (**c**) *rac*-TM1/*S*-TM2 (*rac*/*S*) and *rac*-TM1/*R*-TM2 (*rac*/*R*) with a weight ratio of 25/1 in THF (~10^-3^ mol L^-1^) under ambient conditions.


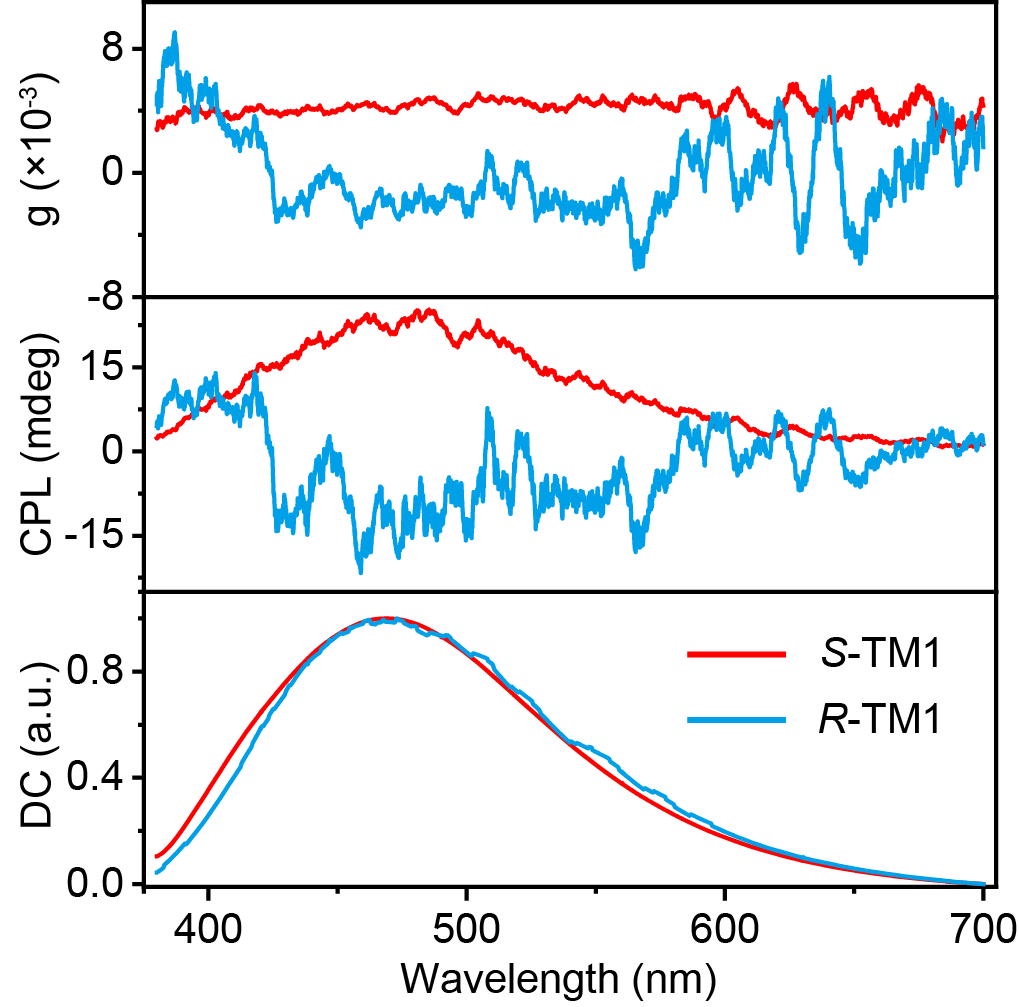


**Figure S43.** Circularly polarized luminescence (CPL) spectra of *S*-TM1 and *R*-TM1 crystals excited by 320 nm UV light under ambient conditions.


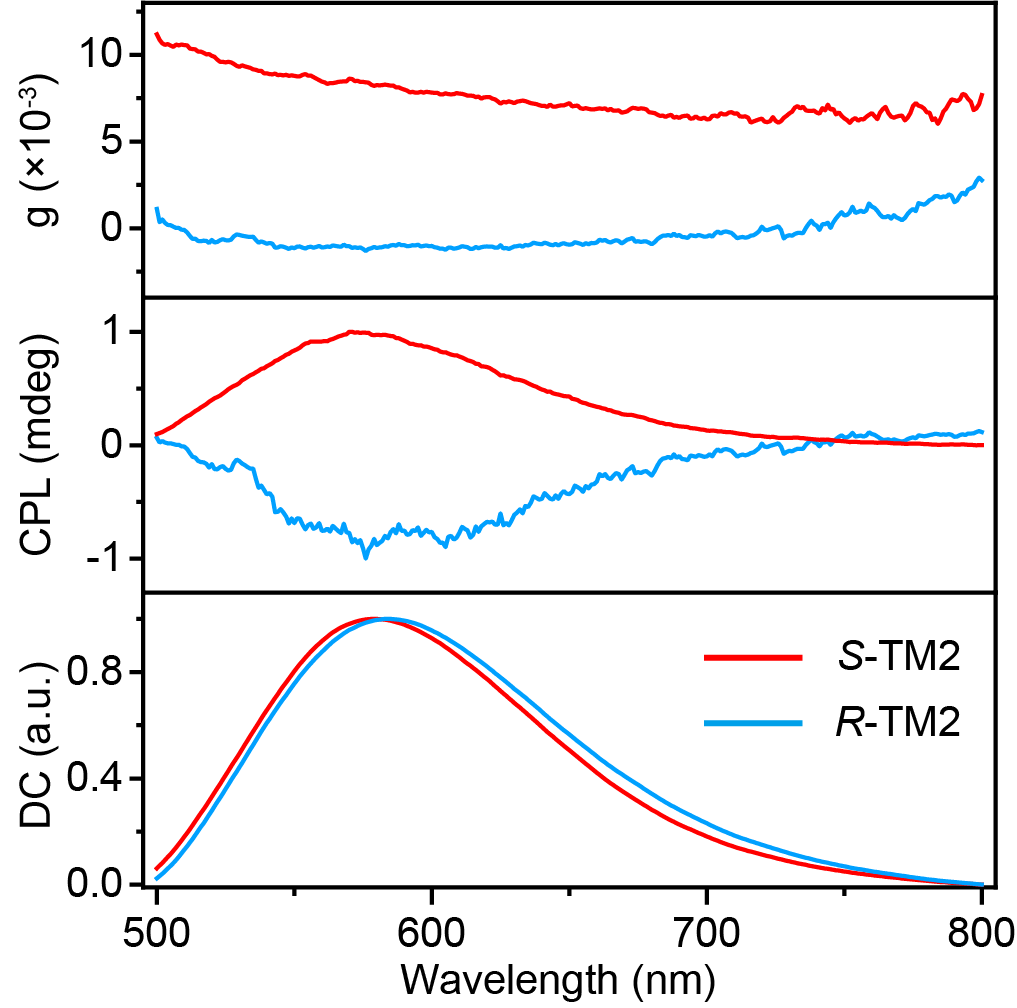


**Figure S44.** CPL spectra of *S*-TM2 and *R*-TM2 crystals excited by 450 nm UV light under ambient conditions.


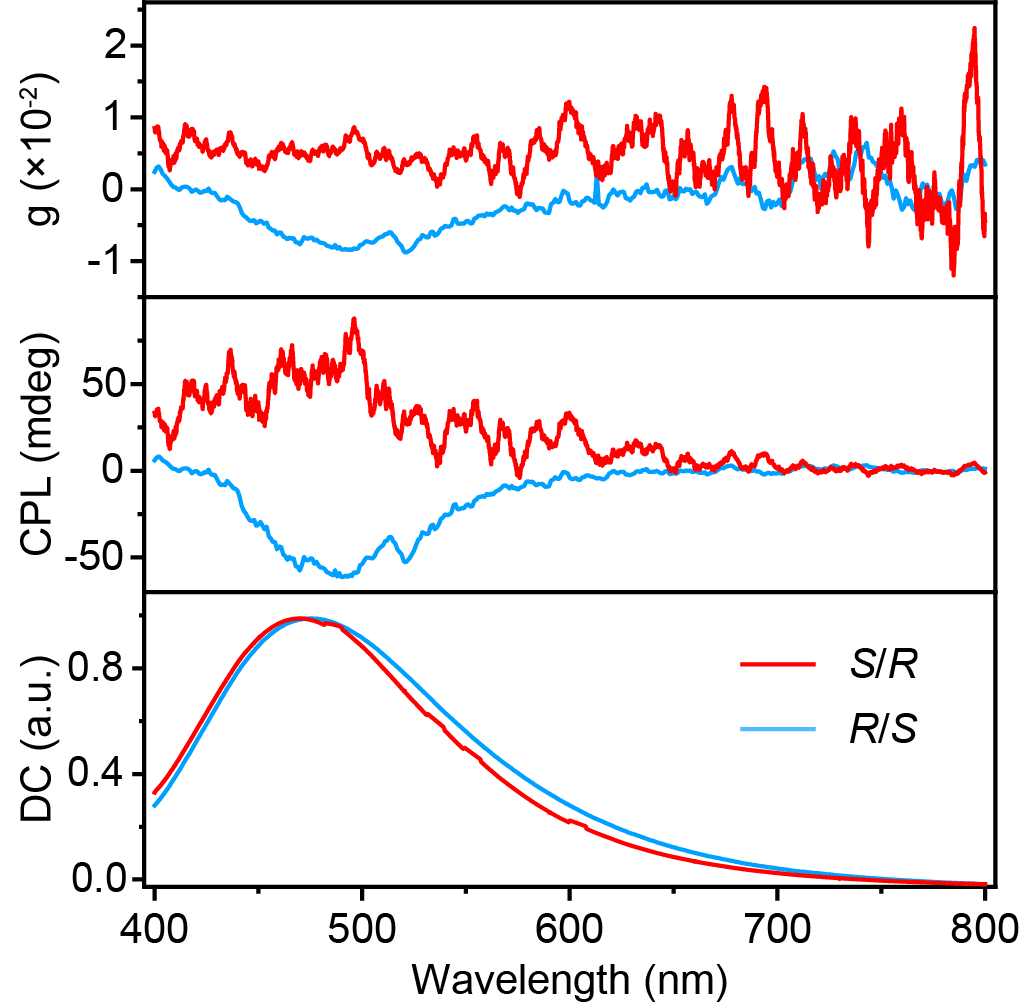


**Figure S45.** CPL spectra of *S*-TM1/*R*-TM2 (*S*/*R*) and *R*-TM1/*S*-TM2 (*R*/*S*) doped crystals with a weight ratio of 25/1 excited by 350 nm UV light under ambient conditions.


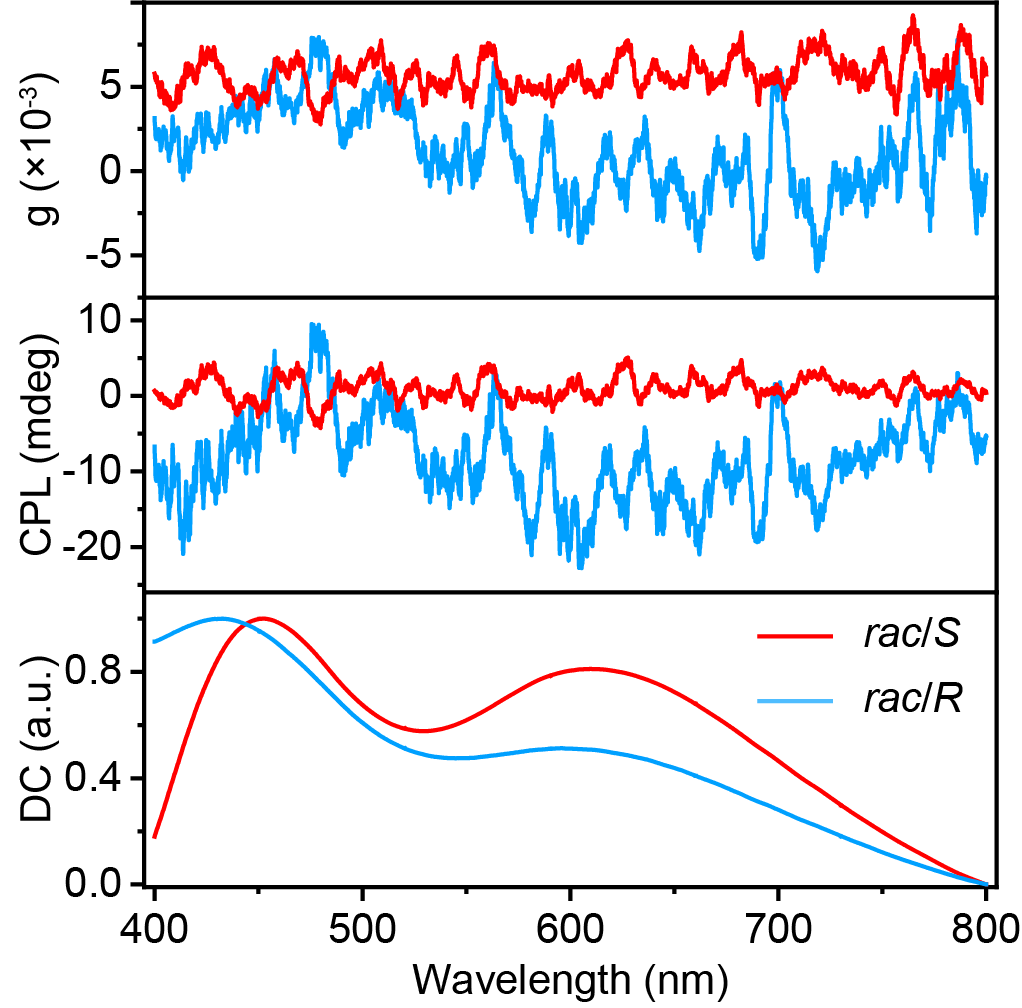


**Figure S46.** CPL spectra of *rac*-TM1/*S*-TM2 (*rac*/*S*) and *rac*-TM1/*R*-TM2 (*rac*/*R*) doped crystals with a weight ratio of 25/1 excited by 350 nm UV light under ambient conditions.


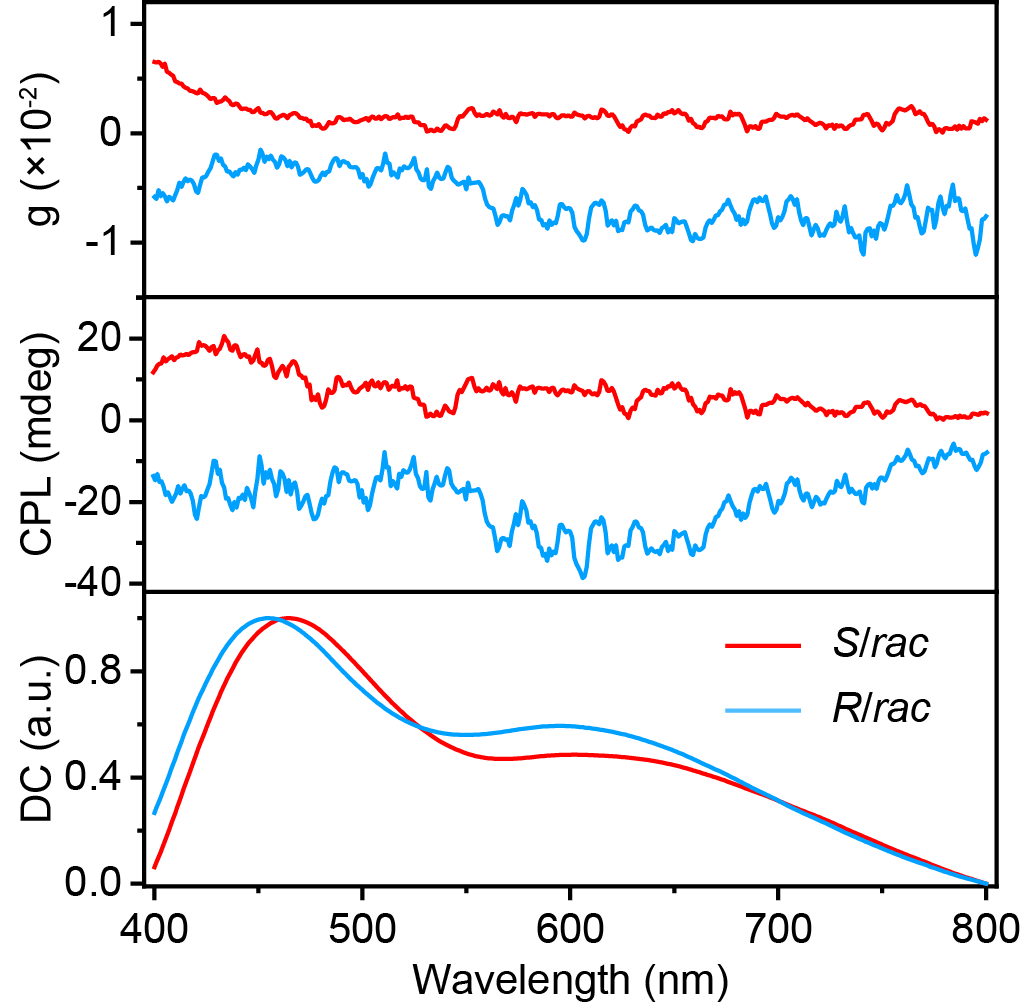


**Figure S47.** CPL spectra of *S*-TM2/*rac*-TM1 (*S/rac*) and *R*-TM1/*rac*-TM2 (*R*/*rac*) doped crystals with a weight ratio of 25/1 excited by 350 nm UV light under ambient conditions.


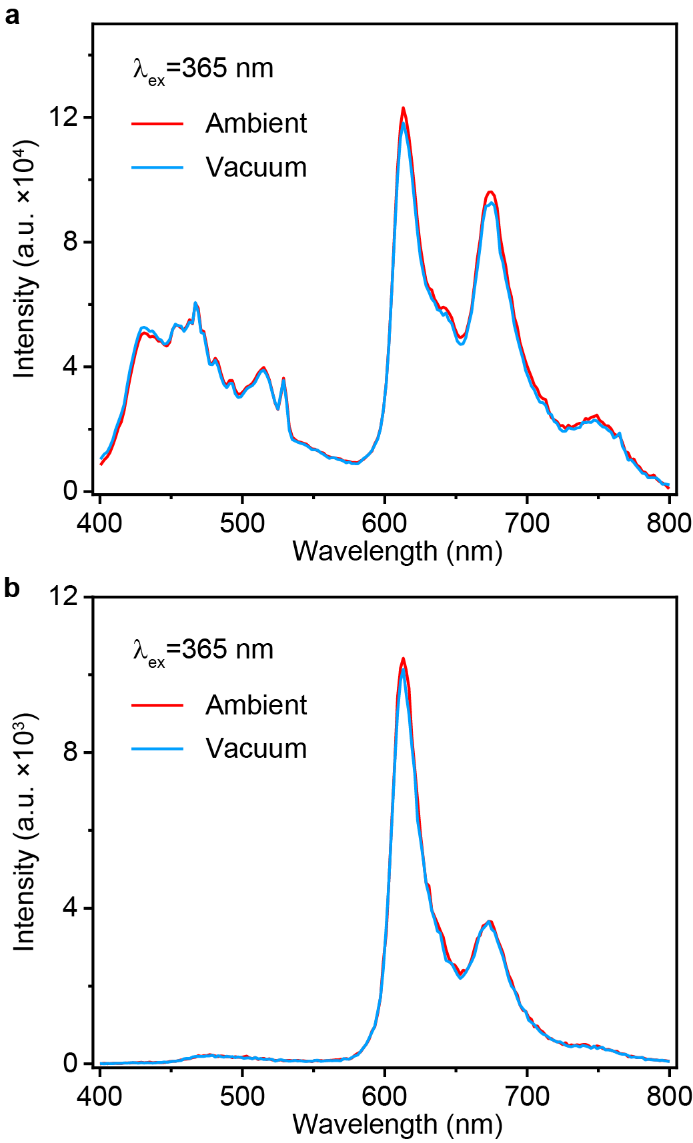


**Figure S48.** (**a**) SSPL and (**b**) delayed PL spectra of *S*-TM1/*S*-TM2 doped crystals with a weight ratio of 25/1 under ambient and vacuum conditions.


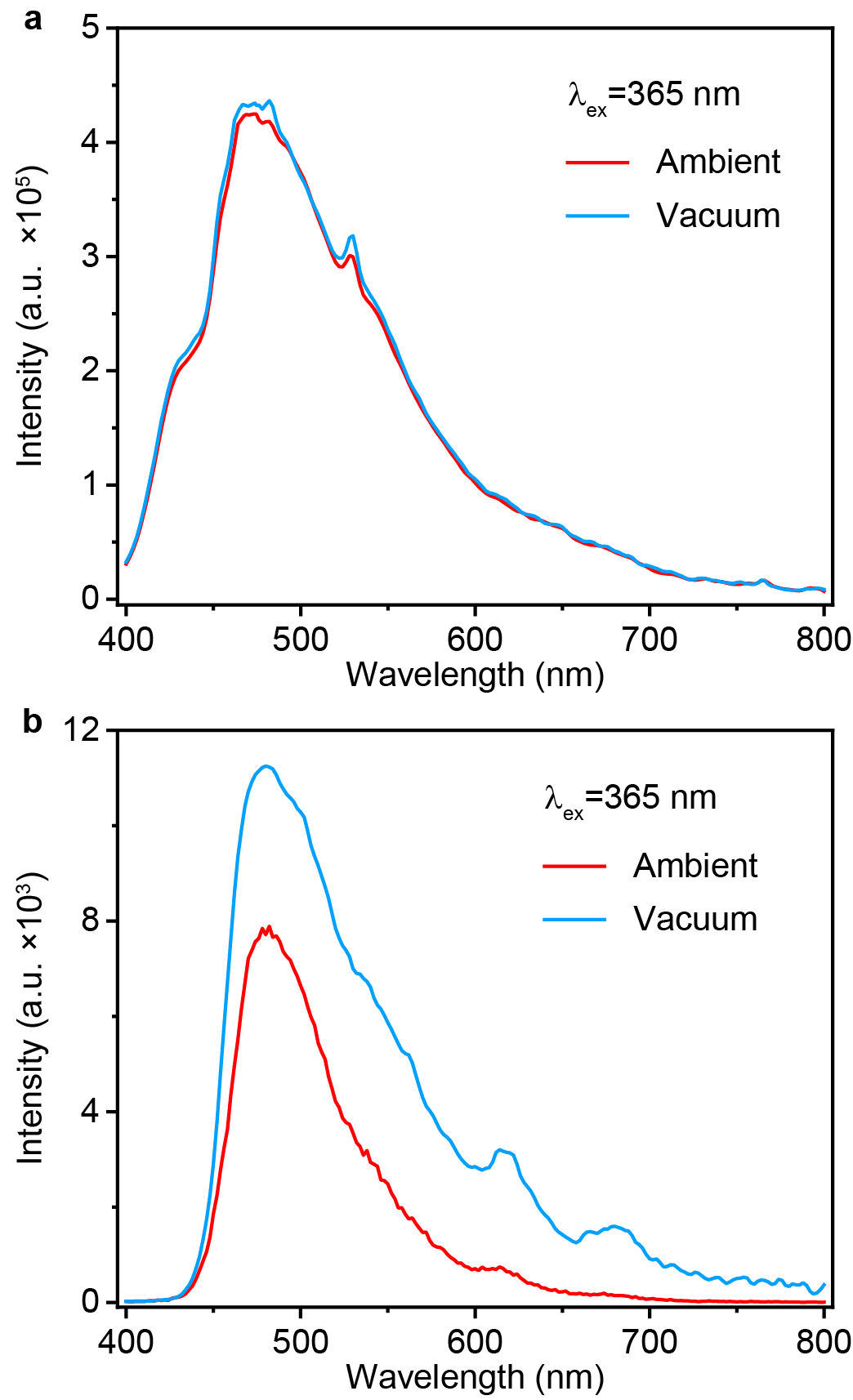


**Figure S49.** (**a**) SSPL and (**b**) delayed PL spectra of *S*-TM1/*R*-TM2 doped crystals with a weight ratio of 25/1 under ambient and vacuum conditions.


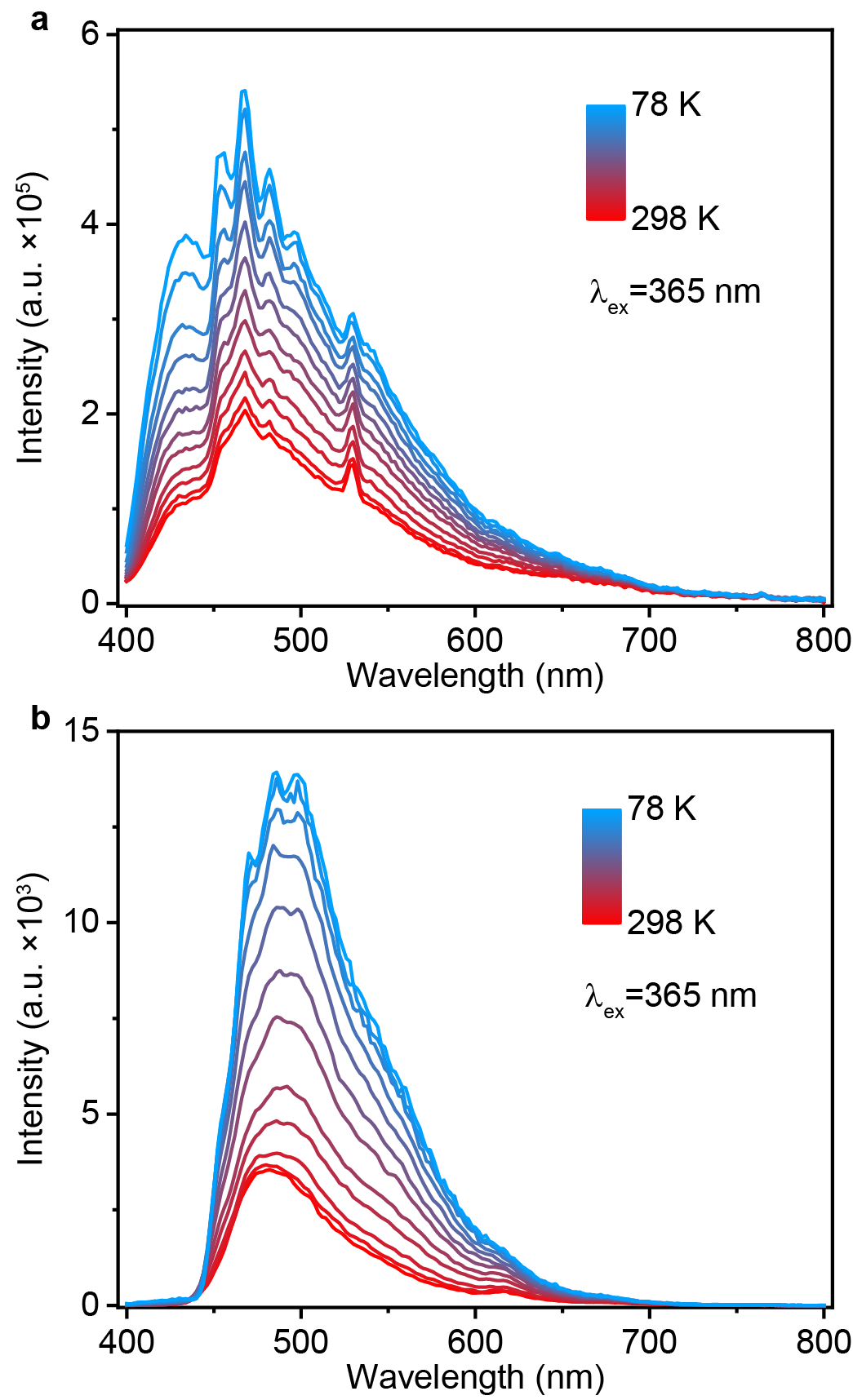


**Figure S50.** Temperature dependent (**a**) SSPL and (**b**) delayed PL spectra of *S*-TM1/*R*-TM2 crystals.


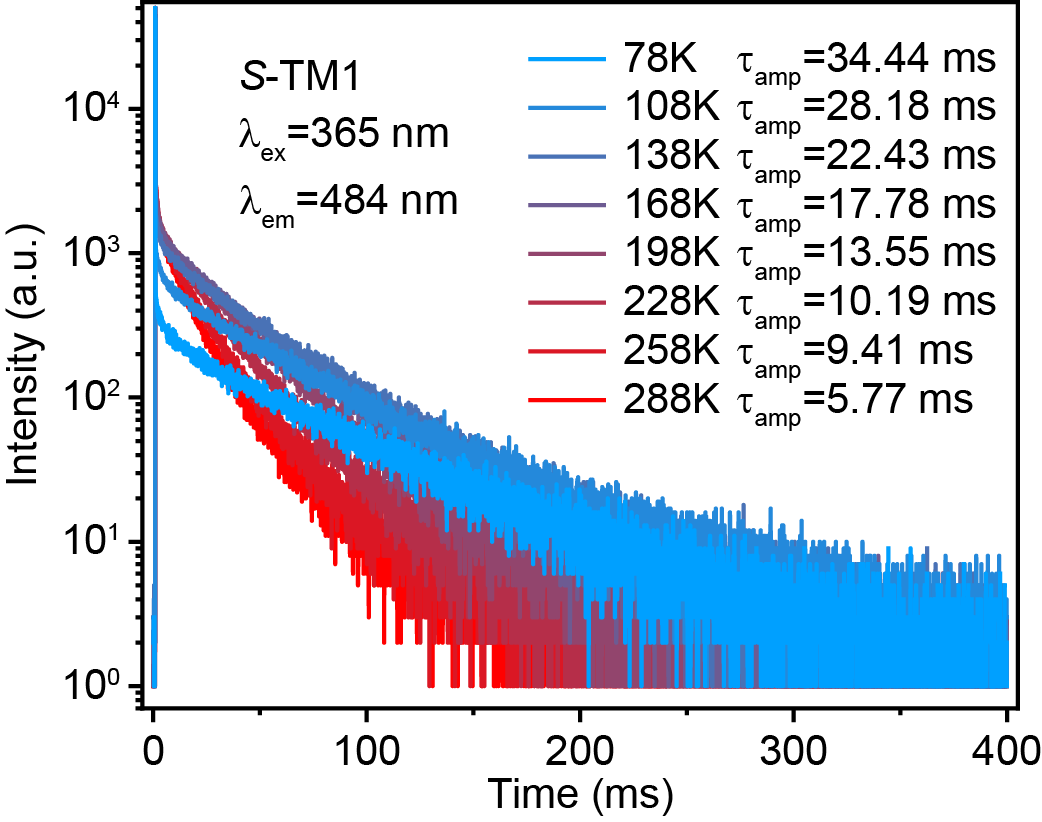


**Figure S51.** Temperature dependent lifetimes of *S*-TM1 crystal.


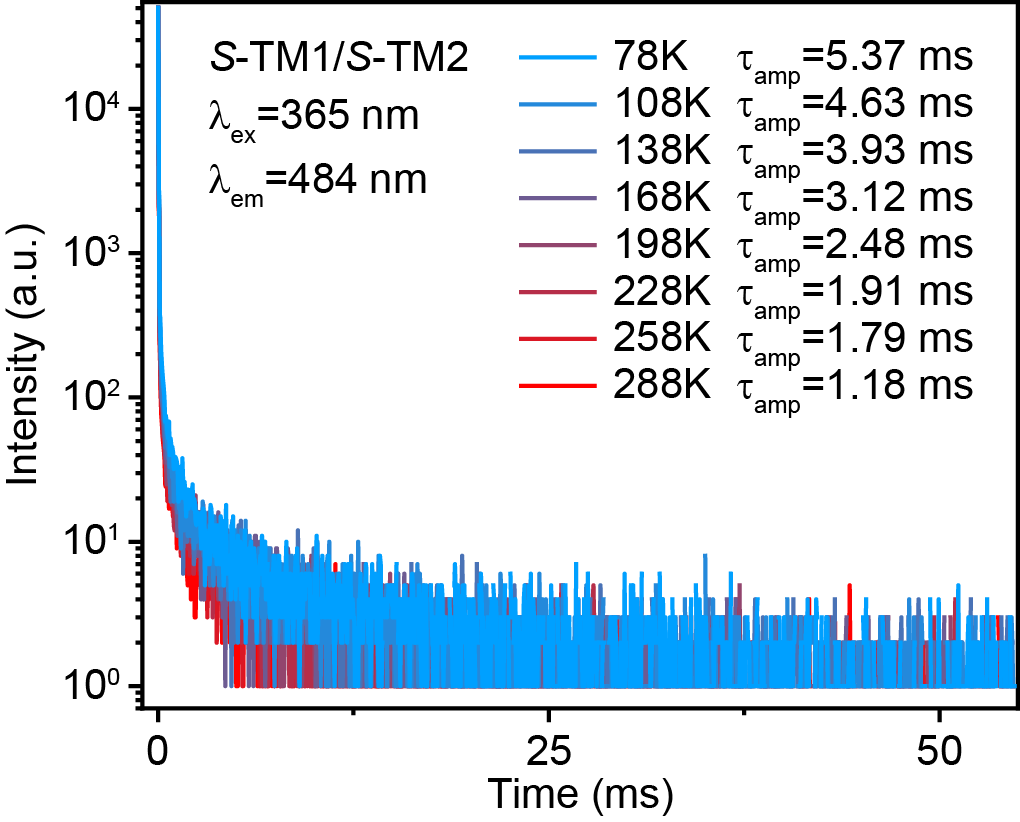


**Figure S52.** Temperature dependent lifetimes of *S*-TM1/*S*-TM2 (25/1) crystals.


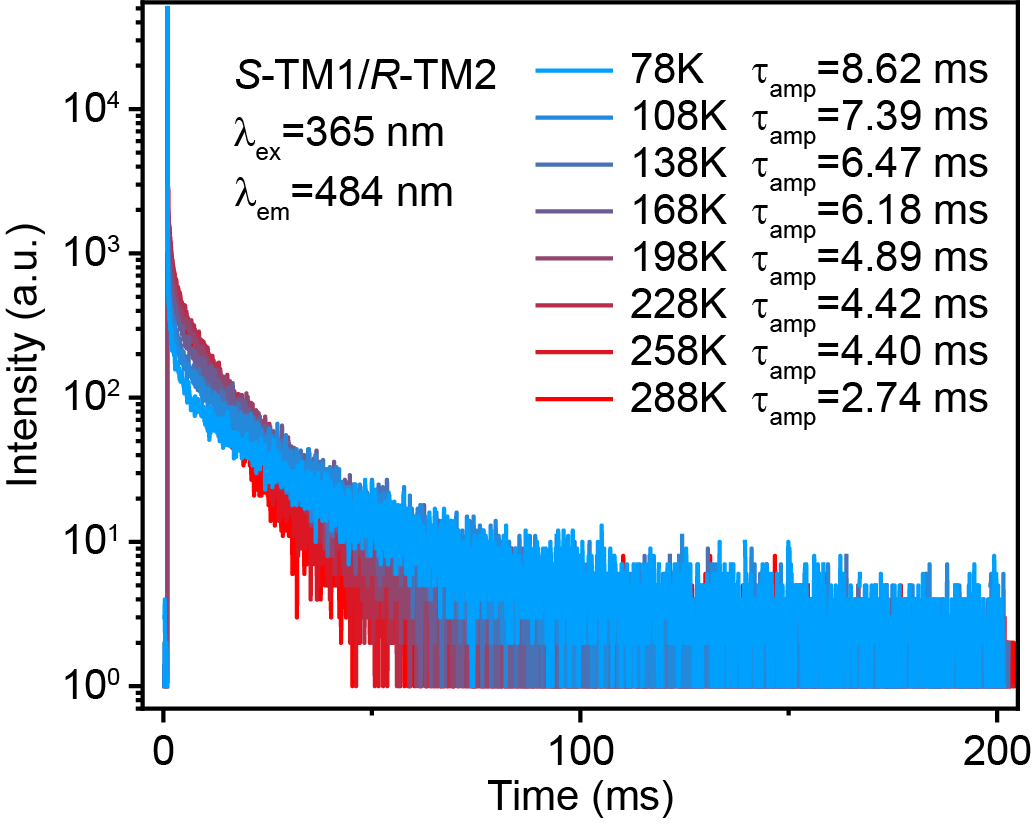


**Figure S53.** Temperature dependent lifetimes of *S*-TM1/*R*-TM2 (25/1) crystals.


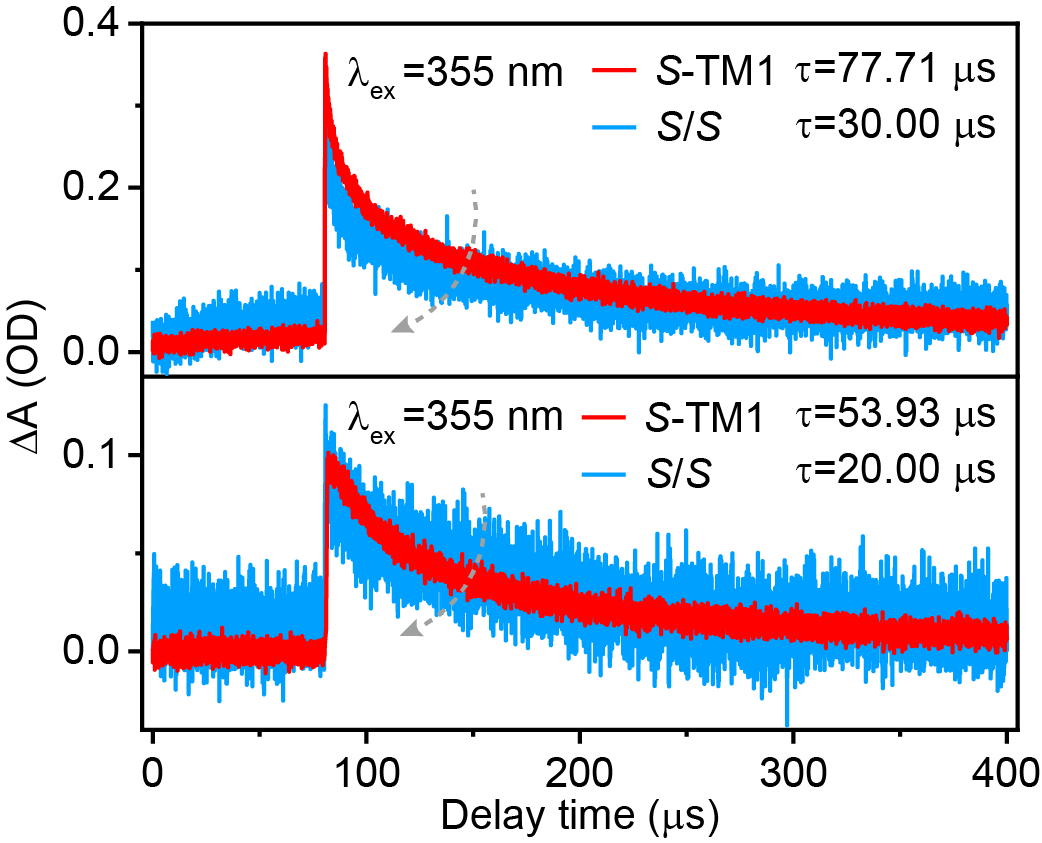


**Figure S54.** Transient absorption lifetimes of *S*-TM1 and *S*-TM1/*S*-TM2 (25/1) at 526 and 567 nm.


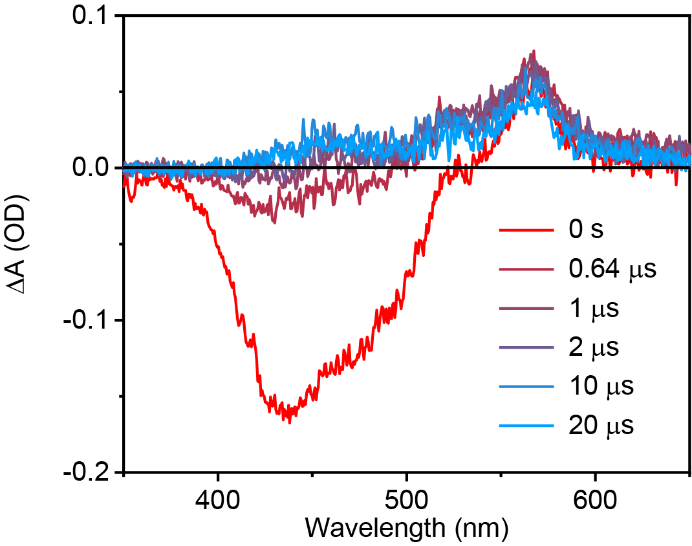


**Figure S55.** Transient absorption (TA) spectra of *S*-TM1/*R*-TM2 doping crystals (25/1) at the delay time range of 0-20 μs.


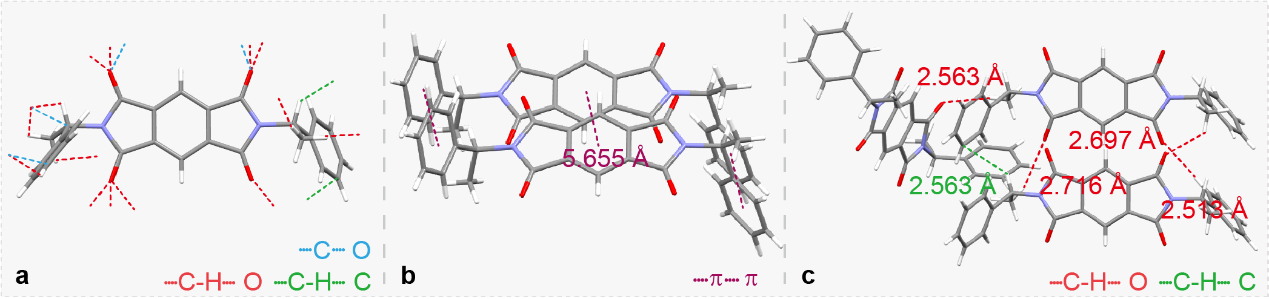


**Figure S56.** Intermolecular interactions of monomer (**a**) and selected dimers (**b**, **c**) in *S*-TM1.


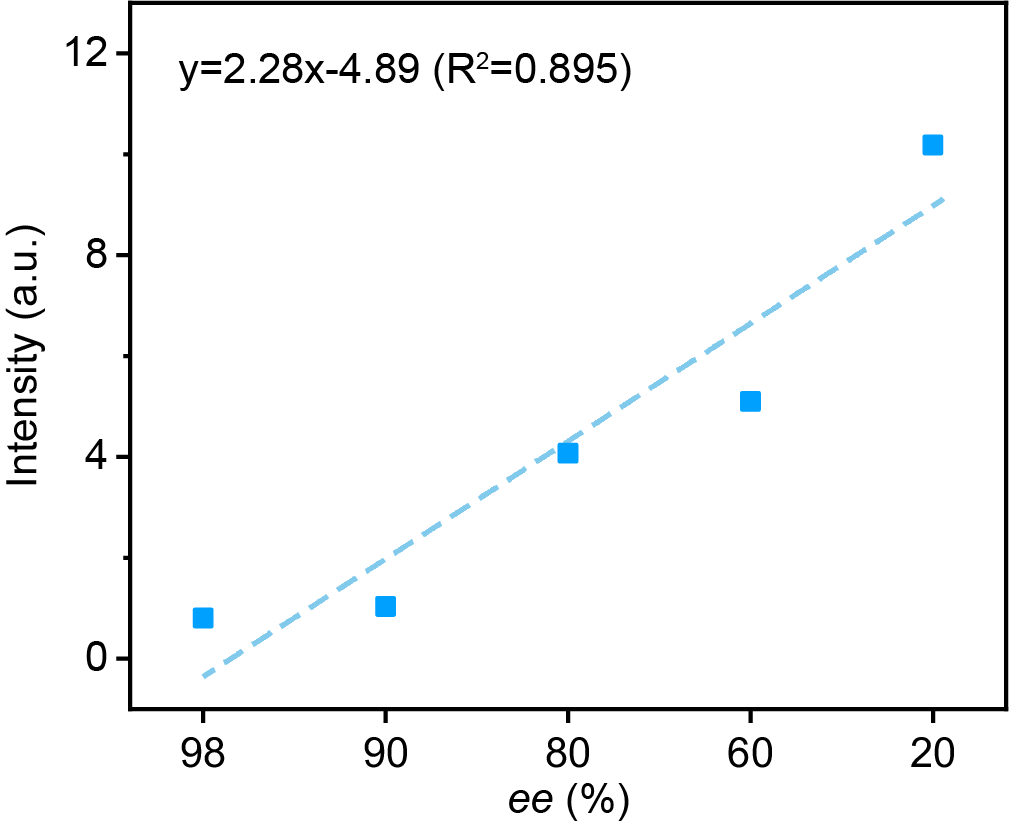


**Figure S57.** Relationship of SSPL relative intensity of emission bands at 612 nm of *S*-TM1/*R*-TM2 doped crystals with a weight ratio of 25/1, which has different dope percent of *S*-TM2 under ambient conditions.

# 4. Theoretical calculations

Density functional theory (DFT) and time-dependent DFT (TD-DFT) simulations were performed with Gaussian 09 package. All the computational models were built from the single-crystal structures without further geometry optimization. The excitation energy of the n-th singlet state (S_n_) and the *n*-th triplet state (T_n_) states were calculated at TD-DFT method of B3LYP/6-31G (d, p) level based on the monomer and selected aggregates extracted from the single-crystal. Frontier molecular orbital distributions were calculated based on the monomer and selected aggregates extracted from the single-crystal at B3LYP/6-31G. Electron density differences (EDD) upon the S_0_→S_n_ and S_0_→T_n_ transitions based on the single crystal structures were carried out using Multiwfn by subtracting the electron density of grounded-state (S_0_) from that of interested singlet or triplet excited states.

**Table S2.** Singlet and triplet excited states transition configurations of *R*-TM1 revealed by TD-DFT calculations.

| Excited state | *n*-th | Energy (eV) | Transition configuration (%) |
| --- | --- | --- | --- |
| S_n_ | 1 | 3.1345 | H→L (96.1) |
| T_n_ | 1 | 2.8753 | H-5→L (33.4); H→L (33.1); H-10→L (10.2); H-1→L (9.8) |
|  | 2 | 3.0296 | H-1→L (37.3); H-4→L (32.0); H→L (18.8) |
|  | 3 | 3.1512 | H-4→L (26.0); H-2→L (16.5); H→L (16.0); H-6→L (11.8) |
|  | 4 | 3.1666 | H-1→L(28.3); H-4→L(18.6); H-6→L (17.2) |
|  | 5 | 3.2607 | H-6→L (21.0); H→L (18.8); H-10→L (18.3); H-2→L (10.6) |
|  | 6 | 3.3143 | H-2→L (57.6); H-6→L (13.8); H-3→L (6.7) |
|  | 7 | 3.3676 | H-3→L (70.5); H-6→L (15.5); H-4→L (2.6) |
|  | 8 | 3.5138 | H-7→L (30.0); H-5→L (22.0); H-10→L (17.1); H-10→L (11.5) |
|  | 9 | 3.5562 | H-7→L (41.1); H -5→L (13.3); H-10→L (10.7); H-3→L (6.6); H-7→L+2(5.5) |
|  | 10 | 3.6847 | H-8→L (88.8); H-10→L (4.2); H-9→L (2.1) |
|  | 11 | 3.8136 | H-2→L+5(31.0); H-1→L+4(28.0); H-9→L (10.5) |
|  | 12 | 3.8222 | H→L+3(41.6); H-3→L+6(32.0); H-3→L+3(5.8); H→L+6(5.1); H→L+4(5.0) |
|  | 13 | 3.8272 | H-9→L (50.2); H-10→L (7.5); H-1→L (6.9); H-2→L+5(6.5) |
|  | 14 | 3.8769 | H-11→L (65.9); H-9→L+2(7.2); H-4→L+1(6.7) |
|  | 15 | 4.1429 | H→L+1(47.1); H-5→L+1(22.3); H-8→L+1(11.8); H-1→L+1(8.0) |

**Table S3.** Singlet and triplet excited states transition configurations of *S*-TM1 revealed by TD-DFT calculations.

| Excited state | *n*-th | Energy (eV) | Transition configuration (%) |
| --- | --- | --- | --- |
| S_n_ | 1 | 3.1358 | H→L (96.0) |
| T_n_ | 1 | 2.8776 | H-5→L (33.2); H→L (33.0); H-10→L (10.2); H-1→L (9.7) |
|  | 2 | 3.0318 | H-1→L (35.1); H-4→L (33.2); H→L (20.0) |
|  | 3 | 3.1568 | H-4→L (18.8); H→L (18.1); H-2→L (16.7); H-6→L (16.1); H-5→L (14.7) |
|  | 4 | 3.1696 | H-1→L (32.2); H-4→L (25.4); H-6→L (12.7); H-3→L (5.4) |
|  | 5 | 3.2667 | H-6→L (21.0); H-10→L (18.7); H→L (16.8); H-2→L (11.8) |
|  | 6 | 3.3204 | H-2→L (55.2); H-6→L (13.0); H-3→L (8.3); H-1→L (6.4); H-4→L (6.1) |
|  | 7 | 3.3665 | H-3→L (68.7); H-6→L (16.0); H-2→L (2.8) |
|  | 8 | 3.5247 | H-4→L (30.9); H-5→L (21.2); H-10→L (17.2); H-2→L (11.7) |
|  | 9 | 3.5580 | H-7→L (40.0); H-5→L (14.1); H-10→L (11.0); H-3→L (7.6) |
|  | 10 | 3.6830 | H-8→L (88.9); H-10→L (4.1); H-9→L (2.1) |
|  | 11 | 3.8250 | H-9→L (30.6); H-2→L+5(19.9); H-1→L+4(15.5); H-1→L+5(4.3) |
|  | 12 | 3.8350 | H→L+3(25.5); H-3→L+6(8.8); H-9→L (8.2); H-2→L+5(8.5) |
|  | 13 | 3.8352 | H-9→L (19.7); H→L+3(14.0); H-3→L+6(13.3); H-2→L+5(10.4) |
|  | 14 | 3.8801 | H-11→L (66.4); H-9→L+2(7.2); H-4→L+1(6.8); H-4→L+1(4.8) |
|  | 15 | 4.1424 | H→L+1(37.5); H-8→L+1(27.0); H-5→L+1(17.4); H-1→L+1(5.2) |


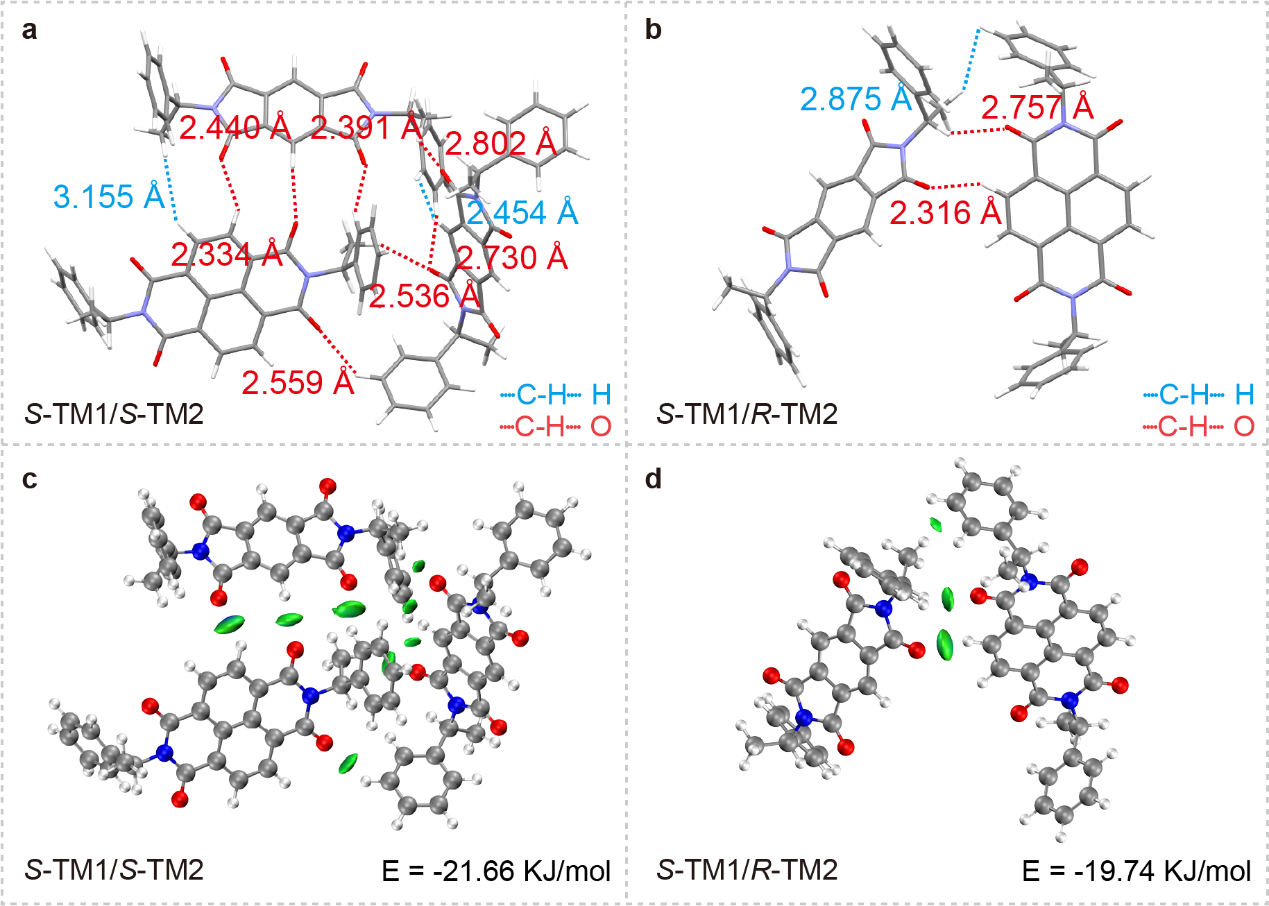


**Figure S58.** Intermolecular force (**a, b**) and binding energy (**c, d**) in the *S*-TM1/*R*-TM2 and *S*-TM1/*S*-TM2 doping crystals.


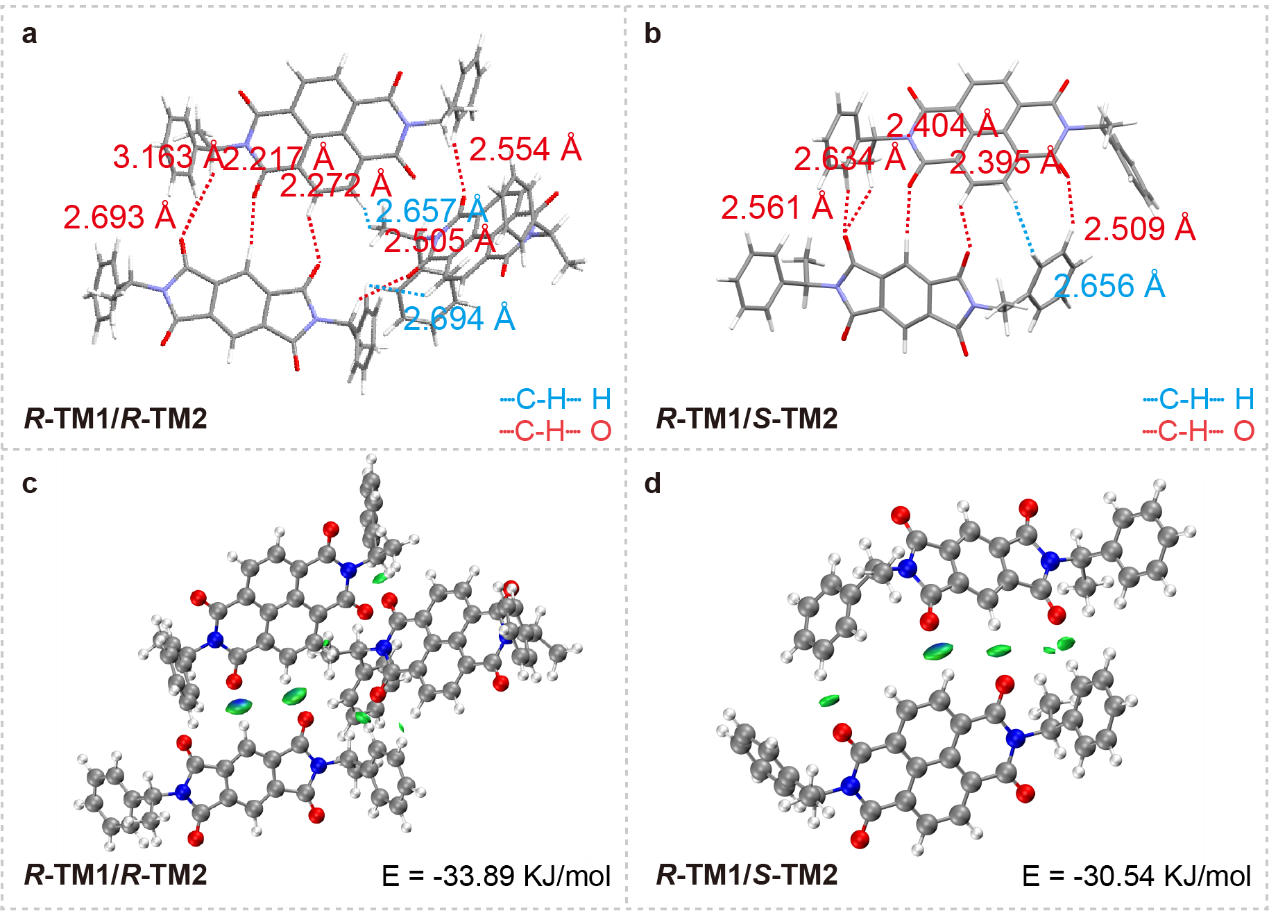


**Figure S59.** Intermolecular force (**a**, **b**) and binding energy (**c**, **d**) in the *R*-TM1/*R*-TM2 and *R*-TM1/*S*-TM2 doping crystals.


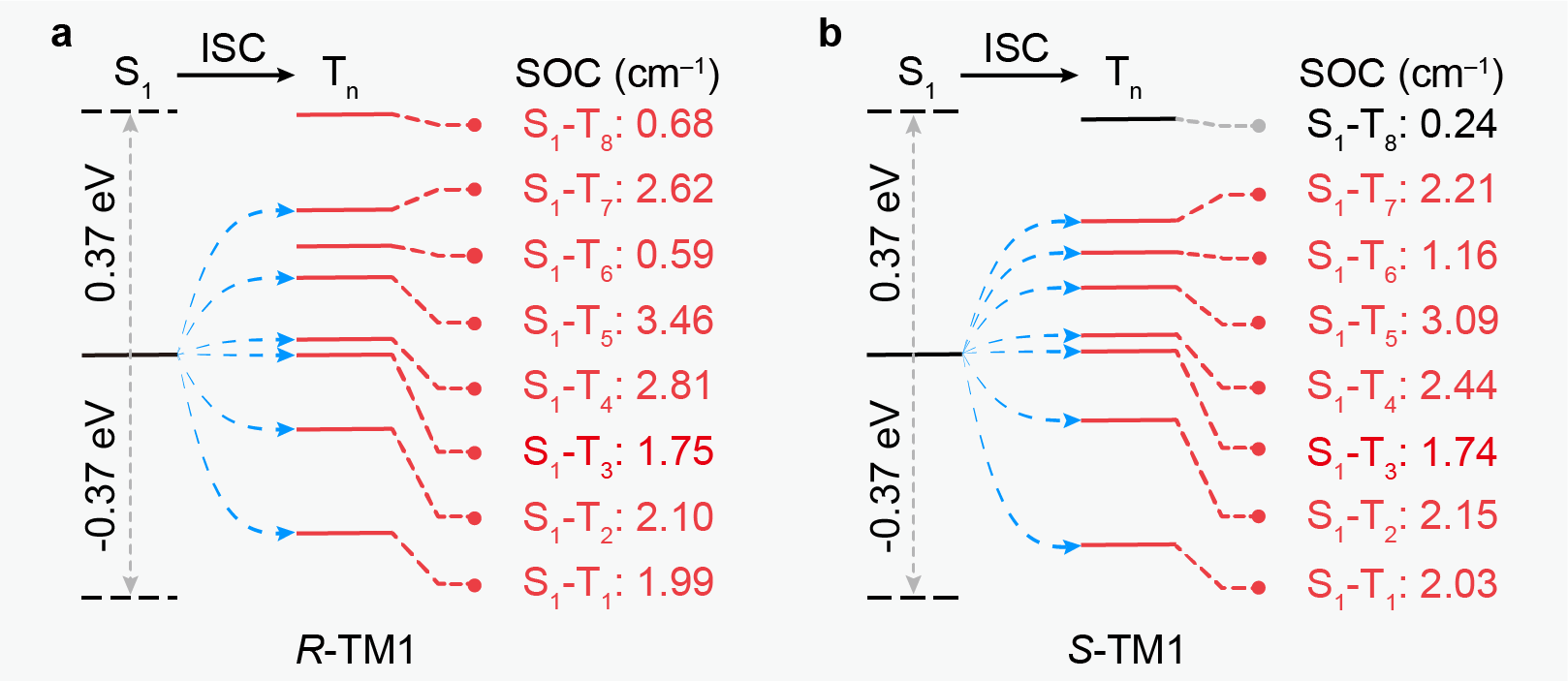


**Figure S60.** Theoretically calculated S_1_ and T_n_ energies and the corresponding SOC constants of S_1_→T_n_ of (**a**) *R*-TM1 and (**b**) *S*-TM1.

**Table S4.** Photophysical properties of *S*-/*R*-TM1, *S*-/*R*-TM2 and doped crystals under ambient conditions.

| Compound | *λ*_ex_  (nm) | *λ*_em_  (nm) | τflu. amp  (ns) | τphos. int  (ms) | τphos. amp  (ms) | PLQY  (%) | *Φ*_ET_  (%) | *g*_lum_ |
| --- | --- | --- | --- | --- | --- | --- | --- | --- |
| *S*-TM1 | 384 | 484 | 0.28 | 17.50 | 5.77 | 1.86 | - | 4.08×10^-3^ |
| *R*-TM1 | 384 | 484 | 0.29 | 15.78 | 5.63 | 1.69 | - | -1.88×10^-3^ |
| *S*-TM2 | 484 | 556 | 8.30 | - | - | - | - | 8.40×10^-3^ |
|  |  | 584 | 8.63 | - | - | - |  |  |
| *R*-TM2 | 484 | 552 | 7.19 | - | - | - | - | -1.19×10^-3^ |
|  |  | 584 | 7.45 | - | - | - |  |  |
| *S*-TM1/*S*-TM2 | 365 | 484 | - | 2.27 | 1.18 | 1.78 | 79.55 | 4.47×10^-2^ |
|  |  | 612 | - | 32.68 | 32.68 |  |  |  |
| *S*-TM1/*R*-TM2 | 365 | 484 | - | 5.81 | 2.70 | 0.91 | 53.21 | 4.08×10^-3^ |
|  |  | 612 | - | 8.11 | 2.04 |  |  |  |
| *S*-TM1/*rac*-TM2 | 365 | 484 | - | 7.78 | 1.57 | 0.84 | 72.79 | 2.10×10^-3^ |
|  |  | 612 | - | 30.04 | 30.04 |  |  |  |
| *R*-TM1/*R*-TM2 | 365 | 484 | - | 3.30 | 1.80 | 0.75 | 71.94 | -2.38×10^-2^ |
|  |  | 612 | - | 32.47 | 32.47 |  |  |  |
| *R*-TM1/*S*-TM2 | 365 | 484 | - | 14.91 | 3.47 | 0.64 | 38.37 | -6.61×10^-3^ |
|  |  | 612 | - | 12.82 | 2.61 |  |  |  |
| *R*-TM1/*rac*-TM2 | 365 | 484 | - | 5.76 | 1.14 | 0.90 | 79.75 | -8.57×10^-3^ |
|  |  | 612 | - | 29.42 | 29.42 |  |  |  |
| *rac*-TM1/*S*-TM2 | 365 | 484 | - | 1.95 | 1.26 | 0.70 | 77.62 | 2.52×10^-3^ |
|  |  | 612 | - | 33.01 | 33.01 |  |  |  |
| *rac*-TM1/*R*-TM2 | 365 | 484 | - | 2.49 | 1.32 | 0.50 | 76.55 | -6.53×10^-3^ |
|  |  | 612 | - | 31.89 | 31.89 |  |  |  |
| *rac*-TM1/*rac*-TM2 | 365 | 484 | - | 0.34 | 0.22 | 0.64 | 96.13 | - |
|  |  | 612 | - | 27.84 | 27.84 |  |  |  |

**Table S5.** The average lifetime and energy transfer efficiency of *S*-TM1/*S*-TM2 and *S*-TM1/*R*-TM2 with different temperature.

|  | Temperature (K) | 78 | 108 | 138 | 168 | 198 | 228 | 258 | 288 |
| --- | --- | --- | --- | --- | --- | --- | --- | --- | --- |
| *S*-TM1 | τ_amp_ | 34.44 | 28.18 | 22.43 | 17.78 | 13.55 | 10.19 | 9.41 | 5.77 |
| *S*-TM1/*S*-TM2 | τ_amp_ | 5.37 | 4.63 | 3.93 | 3.20 | 2.48 | 1.91 | 1.79 | 1.18 |
|  | *Φ*_ET_ | 84.41 | 83.57 | 82.48 | 82.03 | 81.70 | 81.26 | 80.98 | 79.55 |
| *S*-TM1/*R*-TM2 | τ_amp_ | 8.62 | 7.39 | 6.47 | 6.18 | 4.89 | 4.42 | 4.40 | 2.74 |
|  | *Φ*_ET_ | 74.97 | 73.78 | 71.16 | 65.24 | 63.91 | 56.62 | 53.24 | 53.21 |
